# *AtMYB12* expression in tomato leads to large scale differential modulation in transcriptome and flavonoid content in leaf and fruit tissues

**DOI:** 10.1038/srep12412

**Published:** 2015-07-24

**Authors:** Ashutosh Pandey, Prashant Misra, Dharmendra Choudhary, Reena Yadav, Ridhi Goel, Sweta Bhambhani, Indraneel Sanyal, Ritu Trivedi, Prabodh Kumar Trivedi

**Affiliations:** 1 Council of Scientific and Industrial Research-National Botanical Research Institute (CSIR-NBRI), Rana Pratap Marg, Lucknow; 226 001, INDIA; 2 CSIR-Central Drug Research Institute (CSIR-CDRI), Endocrinology Division, Jankipuram Extension, Sitapur Road, Lucknow; 226021, INDIA

## Abstract

Plants synthesize secondary metabolites, including flavonoids, which play important role during various stresses for their survival. These metabolites are also considered as health-protective components in functional foods. Flavonols, one of the important groups of flavonoids, apart from performing several roles in plants have been recognized as potent phytoceuticals for human health. Tomato fruits are deficient in this group of flavonoids and have been an important target for enhancing the accumulation of flavonols through genetic manipulations. In the present study, *AtMYB12* transcription factor of the *Arabidopsis* has been expressed under constitutive promoter in tomato. Transgenic tomato lines exhibited enhanced accumulation of flavonols and chlorogenic acid (CGA) in leaf and fruit accompanied with elevated expression of phenylpropanoid pathway genes involved in flavonol biosynthesis. In addition, global gene expression analysis in leaf and fruit suggested that AtMYB12 modulates number of molecular processes including aromatic amino acid biosynthesis, phytohormone signaling and stress responses. Besides this, a differential modulation of the genes in fruits and leaves is reported in this study. Taken together, results demonstrate that modulation of primary carbon metabolism and other pathways by AtMYB12 in tomato may lead to sufficient substrate supply for enhanced content of phenolics in general and flavonols in particular.

The flavonoid group of plant secondary metabolites has tremendous chemical diversity, ubiquitous occurrence and diverse roles in plants. These compounds impart coloration to the plant parts^1^,^2^,^3^, provide UV tolerance^1^,^4^,^5^, act as auxin transport inhibitors^6^,^7^,^8^, signal molecules in plant-microbe interaction^9^,^10^, antimicrobials for pathogens and as insect deterrents and toxicants^11^,^12^,^13^,^14^. In addition, several health beneficial properties of flavonoids such as anticancerous^15^,^16^, antioxidant^17^,^18^,^19^ and antiosteroporotic activities^20^,^21^,^22^ have also been reported. Elucidation of the flavonoid biosynthesis has been a thrust area of research in the last two decades and a wealth of knowledge has been generated pertaining to the structural and regulatory genes involved in the biosynthetic pathway^23^,^24^.

Phenylpropanoid pathway provides precursor to the flavonoid pathway in the form of coumaroyl CoA which is condensed with three molecules of malonyl CoA in a reaction catalyzed by the CHS enzyme (Fig. 1). Following CHS, there are a series of enzymes that catalyze diverse downstream reactions in the pathway leading to the biosynthesis of aglycone backbones^25^,^26^,^27^ and the modifying enzymes such as glycosyltransferases, methyltransferases and acyltransferases which add further chemical diversity to the flavonoids^28^. Phenylpropanoid pathway is also responsible for the biosynthesis of other classes of plant phenolics such as chlorogenic acid (CGA)^29^. The tight spatial and temporal regulation of the genes involved in the pathway has been suggested to be responsible for the differential biosynthesis and accumulation of flavonoids^30^,^31^. The transcription factors or regulatory proteins from diverse families regulate the expression of structural genes involved in flavonoid biosynthesis^23^,^32^,^33^,^34^.

In *Arabidopsis*, three functionally redundant MYB transcription factors, *AtMYB11*, *AtMYB12* and *AtMYB111*, regulate flavonol branch of flavonoid pathway by transcriptionally activating a set of genes comprising *CHS*, *CHI*, *F3H*, *FLS*^35^. As flavonols exhibit several health benefits, considerable efforts have been made to enhance their content through heterologous and homologous expression of structural or regulatory genes of the flavonoid pathway^22^,^34^,^36^,^37^. The MYB family transcription factors which activate expression of multiple genes of the flavonoid biosynthetic pathway have been the most useful regulatory factors in enhancing flavonoid biosynthesis^35^,^38^. The transgenic tobacco plants constitutively expressing *AtMYB12* transcription factor accumulated significantly enhanced level of various flavonols with rutin being the predominant one^14^,^29^. Likewise, the expression of *AtMYB12* under the control of a fruit specific promoter resulted in highly elevated flavonol content in entire tomato fruit including the flesh tissue^29^. On the basis of microarray and metabolome analysis, it has also been demonstrated that the *AtMYB12* expression in the tobacco modulates diverse metabolic sectors in favour of the enhanced accumulation of flavonols as well as those associated with stress response and primary metabolism in tobacco^14^. However, the phytochemical, morphological and transcriptome modulation pertaining to the constitutive expression of *AtMYB12* in edible tissue such as tomato has not been carried out. Such studies are important for assessing the impact of heterologous expression of transcription factor over the overall growth and development of plant species with improved nutritional value to human.

In the present study, transgenic tomato lines constitutively expressing *AtMYB12* transcription factor have been developed. Developed tomato transgenic lines have been used for the detailed phytochemical analysis and global changes in gene expression in both leaf and fruit tissue of the tomato. Our results demonstrate that the constitutive expression of *AtMYB12* in tomato leads to a large scale modulation in transcriptome encompassing genes involved in diverse aspects of plant biology apart from those involved in phenylpropanoid biosynthesis in general and the flavonol biosynthesis in particular.

## Results

### Constitutive expression of *AtMYB12* modulates leaf anthocyanin content and fruit color.

**Figure 2:**
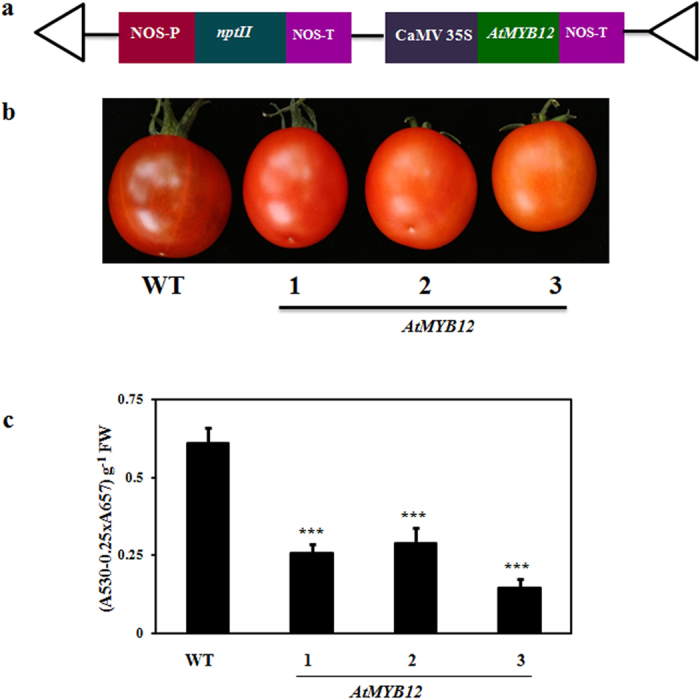
*AtMYB12*-expressing tomato transgenic lines and molecular analysis. (**a**) Schematic representation of T-DNA region of plant expression construct carrying *AtMYB12* in pBI121 vector (pBI121-*AtMYB12*), used for tomato transformation. (**b**) Mature wild type (WT) and transgenic lines (1, 2 and 3) of tomato fruits. (**c**) Total anthocyanin content in leaves of WT and *AtMYB12*-expressing transgenic tomato lines. 1, 2 and 3 represents transgenic lines line 1, line 2 and line 3 respectively. The photograph was taken by AP.

Tomato plants were transformed with the construct carrying AtMYB12 ORF under the control of constitutive promoter CaMV35S (Fig. 2a). Several kanamycin resistant independent tomato transgenic lines were regenerated and grown till maturity. These transgenic lines were morphologically indistinguishable from the WT tomato plants in every respect except for the anthocyanin pigmentation over leaves and petiole as well as peel pigmentation of mature fruits (Fig. 2b). The peel of mature ripe transgenic tomato fruits was slightly orange rather than being red as in case of WT plants. The peel color difference might be due to modulated accumulation of flavonoids. Similar change in color was observed by Luo *et al.*, (2008) and which was in correlation with the expression level of AtMYB12 and the content of flavonol derivatives in the transgenic fruit^29^. The transgenic lines were confirmed for the presence and expression of transgene through genomic DNA PCR and semi-quantitative RT-PCR (Supplementary Fig. 1a and b). No amplicon in genomic DNA PCR or RT-PCR was observed in WT plants whereas amplicon of expected size was observed in transgenic lines expressing *AtMYB12*. These results confirm that selected putative transgenic plants harbor and express *AtMYB12*.

The visible anthocyanin pigmentation over leaves and other aerial parts was drastically reduced in various transgenic lines (Supplementary Fig. 2a). Furthermore, the spectrophotometric analysis also confirmed significant decrease in total anthocyanin content of leaves in *AtMYB12*-expressing transgenic tomato lines (Fig. 2c). Anthocyanin content in leaves of transgenic lines reduced >50% as compared to the WT leaves. Taken together, these results demonstrate that constitutive expression of *AtMYB12* modulates flavonoid biosynthesis in different plant tissues of tomato.

### *AtMYB12* expression leads to enhanced phenolics and flavonoid content in tomato

After preliminary morphological and phytochemical analysis in T0 generation, based on kanamycin selection and transgene expression, out of fifteen, three independent transgenic tomato lines (Line 1, 2 and 3) were used for further analysis. These three independent transgenic lines were selected as the expression of AtMYB12 was higher in these lines in comparison to other lines. The inheritance of transgene in these lines followed the typical mendelian pattern of inheritance as evident by germination of seeds over kanamycin containing MS medium. In tomato, the flavonoid biosynthesis is differentially regulated in leaves and fruits with former containing substantial amount of flavonoids and later with its trace amounts. This is evident from analysis of total phenolics and flavonoid contents in the fruit and leaf of the WT and transgenic plants. Total polyphenolic and flavonoid content in leaf tissue of *AtMYB12*-expressing transgenic plants were higher (upto 1.72 ± 0.25 mg/g FW and 1.18 ± 0.21 mg/g FW, respectively) as compared to the WT tomato leaves (upto 0.25 ± 0.02 mg/g FW and 0.21 ± 0.015 mg/g FW, respectively). Similar to leaf tissue, significant enhancement in polyphenolic and flavonoid content was also observed in fruit tissue. This increase in total polyphenol and flavonoid content were 7- and 6-fold in transgenic lines as compared to WT respectively (Supplementary Fig. 2b and c).

### Modulated content of flavonoids in tomato transgenic lines

**Figure 3:**
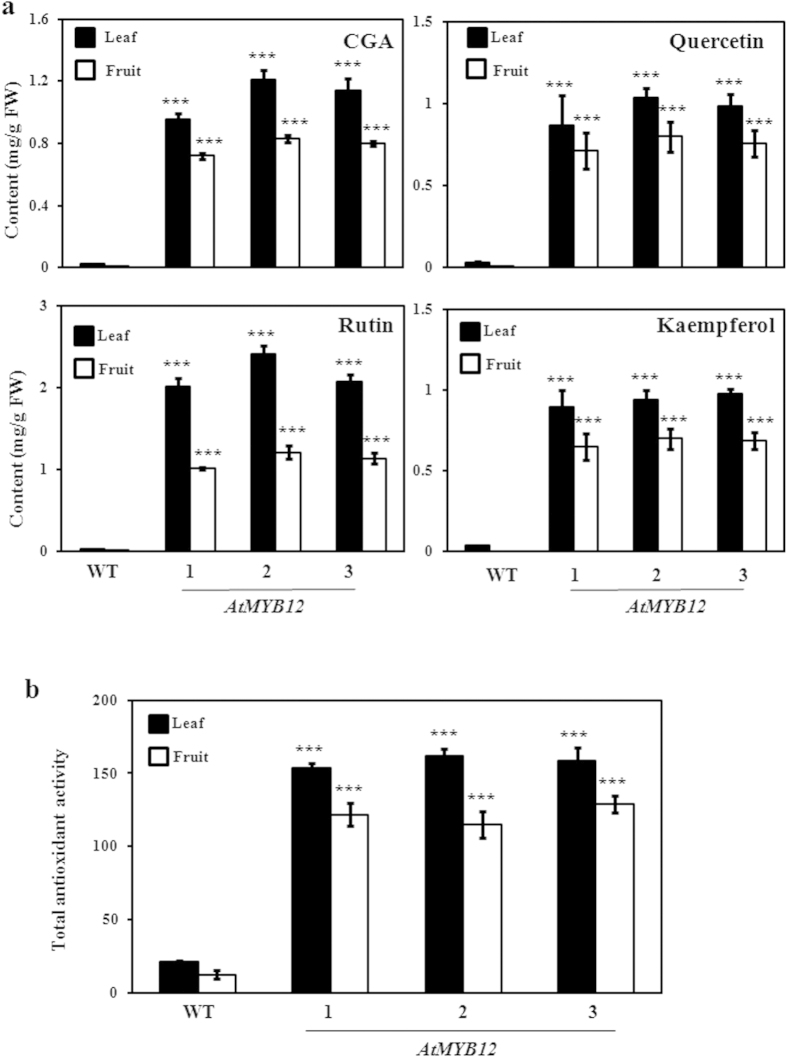
Phytochemical analysis of the methanolic extracts of leaves and fruits of *AtMYB12*-expressing tomato transgenic lines. (**a**) CGA and rutin were quantified by separating non-hydrolyzed methanolic extracts of WT and transgenic lines using HPLC. Quantification of quercetin and kaempferol were carried out using acid-hydrolyzed methanolic extract. (**b**) Measurement of total antioxidant activity using methanolic extracts. Graph has been plotted by Trolox equivalent antioxidant capacity (TEAC). The graph shows values ± SD of three leaves from each of the independent transgenic line. 1, 2 and 3 represent transgenic line 1, line 2 and line 3 respectively.

Quantitative phytochemical analysis was carried out for phenolics and flavonoids such as CGA and flavonols (e.g. rutin, quercetin and kaempferol) using HPLC of methanolic extracts from leaf and fruit tissues of WT and transgenic lines. Analysis of flavonoids suggests enhanced level of different flavonoids in leaves of the transgenic lines expressing *AtMYB12*. The CGA content in leaves of transgenic lines (1.25 ± 0.009 mg/g FW) enhanced up to 24- fold of the WT (0.052 ± 0.003 mg/g FW) levels (Fig. 3a). Significant enhanced level of the rutin (up to 2.5 ± 0.36 mg/g FW) which was about 33-fold higher was also observed in the transgenic tomato lines as compared to the WT plants (0.075 ± 0.009 mg/g FW) (Fig. 3a). To quantify specifically various aglycone forms of flavonoids, the methanolic extracts were acid-hydrolyzed and analyzed through HPLC. The analysis suggested presence of quercetin and kaempferol as aglycone forms of flavonols in leaves of tomato with their contents being significantly higher in transgenic plants (up to 1.04 ± 0.06 mg/g FW and 0.98 ± 0.03 mg/g FW, respectively) as compared to WT plants (up to 0.04 ± 0.002 mg/g FW and 0.032 ± 0.004 mg/g FW, respectively) (Fig. 3a). Both these aglycone forms showed 26- and 30- fold enhancement respectively in leaves of transgenic plants in comparison to WT plants, respectively (Fig. 3a).

Similar to leaf tissue, the contents of CGA and rutin enhanced several fold (158- and 48- fold respectively) in fruits of transgenic plants (up to 0.85 ± 0.075 mg/g FW and 1.21 ± 0.062 mg/g FW, respectively) as compared to the fruits of WT plants (up to 0.0054 ± 0.0014 mg/g FW and 0.025 ± 0.0014 mg/g FW, respectively) (Fig. 3a). The analysis of acid-hydrolyzed methanolic extracts suggested presence of quercetin and kaempferol as aglycone forms of flavonols in fruits of tomato with their contents in transgenic plants (up to 0.81 ± 0.094 mg/g FW and 0.71 ± 0.064 mg/g FW, respectively) while quercetin was present in very less amount (upto 0.0023 ± 0.0015 mg/g FW) and kaempferol being undetectable in WT plants (Fig. 3a). Taken together, these results demonstrate that constitutive expression of *AtMYB12* in tomato leads to enhanced CGA and flavonol content in leaves and fruits of tomato. As a reflection of enhanced polyphenol and flavonoid content, the total antioxidant capacity of transgenic tomato fruits in terms of trolox (6-hydroxy-2,5,7,8-tetramethylchroman-2-carboxylic acid; water soluble analogue of vitamin E) equivalents was also enhanced by 6- fold (Fig. 3b). Enhanced flavonol and CGA contents in *AtMYB12*-expressing transgenic tomato plants may be due to modulation in molecular networks responsible for substrate availability to phenylpropanoid pathway as already been shown in case of tobacco^14^.

### Modulation of expression of genes involved in phenylpropanoid pathway

**Figure 4:**
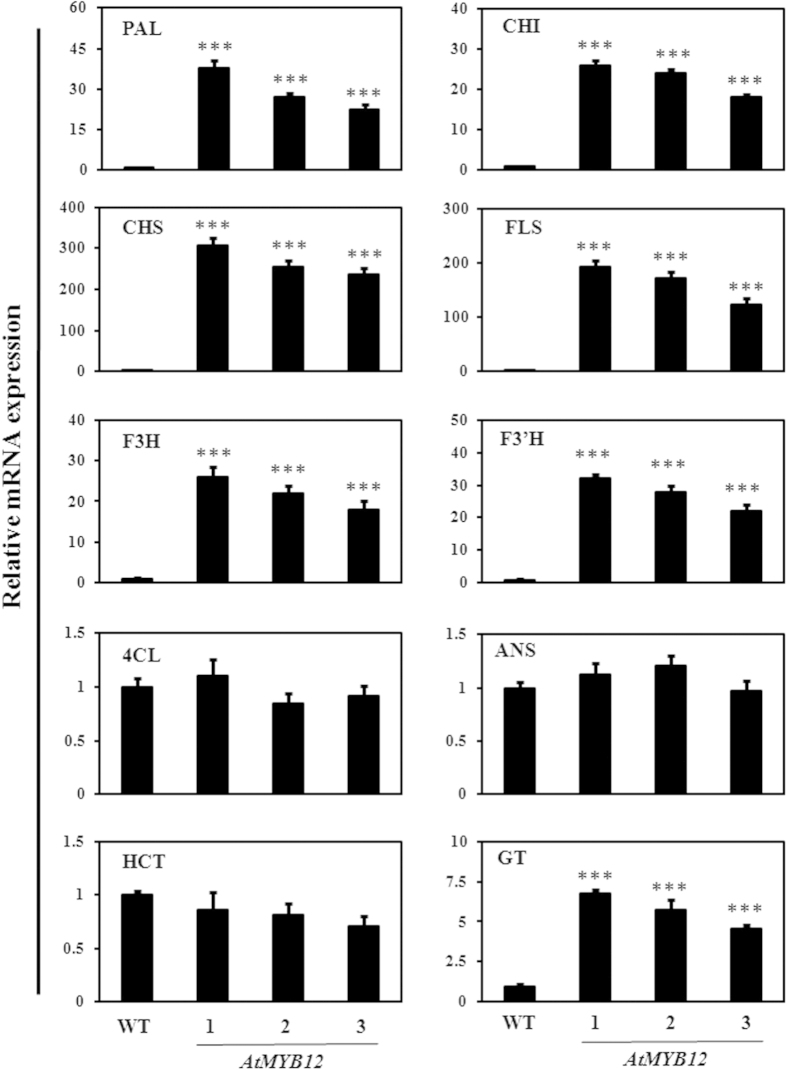
Quantitative expression analyses of the structural genes in fruits of *AtMYB12*-expressing tomato transgenic lines. Expression of structural genes of phenylpropanoid pathway/flavonoid pathway in fruits of the WT and transgenic tomato lines. 1, 2 and 3 represent transgenic line 1, line 2 and line 3 respectively. PAL, Phenylalanine amonia lyase; CHI, Chalcone isomerase; CHS, Chalcone synthase; FLS, Flavonol synthase; F3H, Flavonone-3-hydroxylase; F3′H, flavonoid 3′-hydroxylase; 4CL, 4-coumaroyl CoA ligase; ANS, Anthocyanidine synthase HCT, Hydroxycinnamoyl transferase; GT, Glucosyltransferase.

To study modulation in expression of genes involved in phenylpropanoid pathway, expression profile of different genes in leaf and fruits of WT and transgenic tomato lines were studied through real time PCR. The real time gene expression analysis suggested up-regulation of various genes involved in flavonol biosynthesis such as *PAL*, *4CL*, *CHS*, *CHI* and glycosytransferase (*GT*) in the transgenic tomato lines (Fig. 4 and Supplementary Fig. 3). Maximum enhancement in expression was observed in case of *CHS* (300- and 130- fold enhanced expression in fruit and leaf tissue respectively) and *FLS* (193- and 40- fold enhanced expression in fruit and leaf tissue respectively). The expression of other genes such as *PAL*, *CHI*, *F3H* and *F3′H* was also increased significantly in both leaf and fruit tissue. However out of both the tissues, expression of *HCT, 4CL* and *ANS* was significantly up-regulated only in leaves of the transgenic lines as compared to WT plants. The enhanced expression of glycosyltransferase (7- and 32- fold in leaf and fruit tissue respectively) was observed (Fig. 4 and Supplementary Fig. 3).

### Genome wide modulation in tomato transcriptome by *AtMYB12* expression

In order to study genome-wide modulation by *AtMYB12* in tomato, microarray based comparative gene expression analysis between leaf and fruit tissue of *AtMYB12*-expressing transgenic line and WT tomato plants was carried out. Several differentially regulated genes could be identified in fruits and leaf tissues of *AtMYB12*-expressing transgenic tomato plant in comparison to tissues from WT plants. A combined criterion of 2-fold or greater modulation in expression and P < 0.05 resulted 104 and 41 probe sets as up- and down-regulated, respectively in leaf tissue of transgenic tomato in comparison to WT leaf tissue. These probes sets represented 67 and 20 up- and down-regulated unigenes, respectively (Supplementary Table 1 and 2). Using same criterion, in case of transgenic fruit tissue, 686 and 851 probe sets representing 305 and 419 unigenes were up- and down-regulated, respectively with a representation of down-regulated unigenes, respectively in comparison to fruit from WT plants (Supplementary Table 3 and 4).

**Figure 5:**
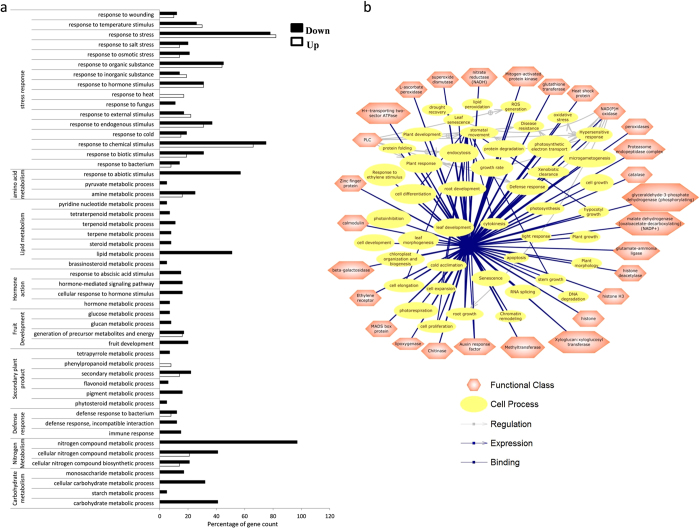
Gene ontology of differentially regulated genes and functional network predicted. (**a**) *Solanum lycopersicum* homologues of the differentally-regulated genes in *AtMYB12*-expressing tomato fruit were identified and grouped with respect to their predicted involvement in different processes using agriGO (level 4). (**b**) *Solanum lycopersicum* homologues of the differentially expressed genes in *AtMYB12*-expressing tomato were identified to construct interactive network using Pathway Studio (Ariadne Genomics, USA).

The analysis suggested differential gene expression in the both tissues with certain genes being commonly up- and down-regulated in both the tissues while others were modulated only in either of the two tissues (Supplementary Fig. 4a and b). The differentially regulated genes in leaves and fruits of transgenic plants were grouped on the basis of their predicted role in different biological processes using agriGO. The analysis suggests that modulation of different biological processes such as secondary metabolism, amino acid metabolism, hormone signaling, lipid and carbohydrate metabolism as well as stress response (Fig. 5a and Supplementary Fig. 5a). Taken together, in comparison to leaf, higher modulation in the fruit transcriptome as well as in various biological processes by *AtMYB12* expression was observed.

### Metabolic networks from transcriptome expression profiles

Analysis of potential metabolic networks from microarray studies is a very useful method for maximizing information^39^. To identify the potential changes in cellular functions, transcriptome interaction networks of all the differentially-regulated data sets from leaf and fruit tissues of transgenic line in comparison to WT plant was analyzed using the software Pathway Studio (Ariadne Genomics, USA). The pathway analysis suggests that *AtMYB12*-expressing transgenic tomato networks related to phenylpropanoid pathway, plant defence, carbohydrate metabolism and amino acid biosynthesis. Our analysis suggests that *AtMYB12*-expression affects secondary metabolism process (Fig. 5b and Supplementary Fig. 5b). Interestingly, in fruit tissue, number of processes related to fruit development and ripening was also affected as analyzed by pathway studio. This can be explained due to differentially regulated genes such as MADS box proteins, expansins and ethylene receptors which are known to regulate fruit development and ripening^40^,^41^,^42^,^43^.

### Validation of modulated expression of genes

**Figure 6:**
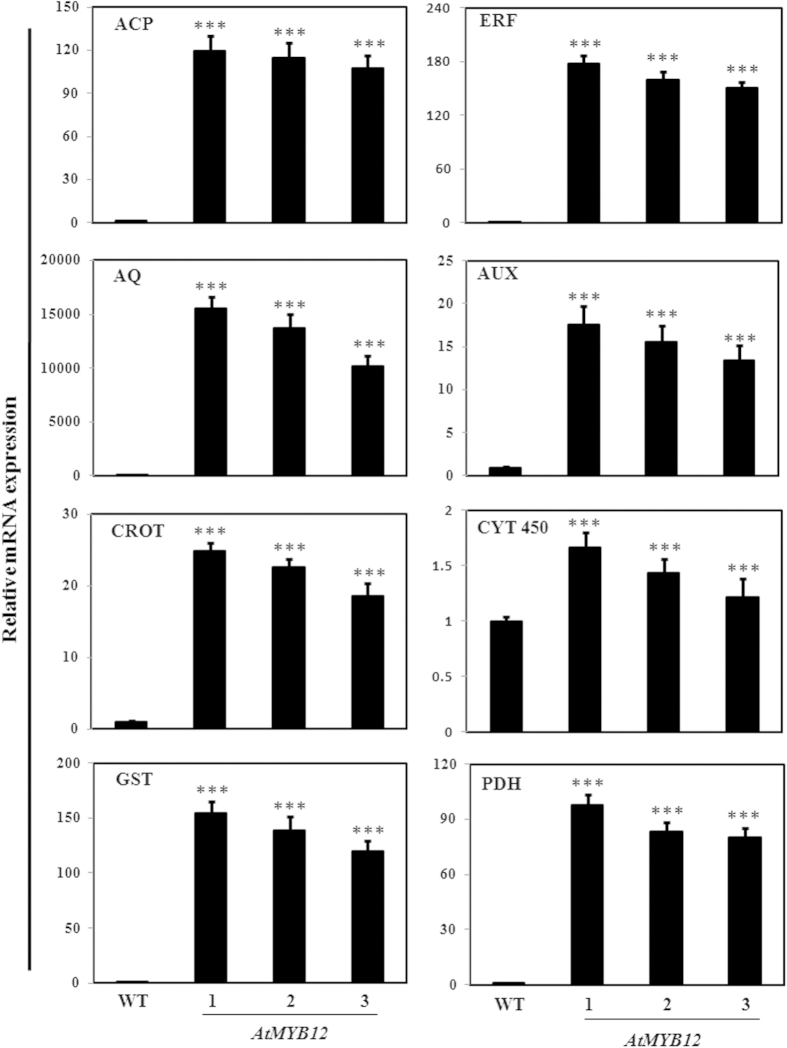
Validation of differential expression of genes involved in different processes in fruits of *AtMYB12*-expressing tomato transgenic lines. 1, 2 and 3 represent transgenic line 1, line 2 and line 3 respectively. ACP-1-aminocyclopropane-1-carboxylate synthase (Solyc08 g081550.2.1 ); AQ, Aquaporin (Solyc12 g044330.1.1); AUX, Auxin efflux carrier family protein (Solyc02 g082450.2.1); CROT, Crotenase (Solyc12 g011160.1.1); CYT450, Cytochrome P450 (Solyc10 g083700.2.1); ERF, Ethylene-responsive transcription factor 4 (Solyc07 g053740.1.1); GST, Glutathione S-transferase (Solyc10 g084400.1.1); PDH, Prephenate dehydratase (Solyc06 g074530.1.1).

Through real time PCR, certain differentially regulated genes putatively involved in various pathways other than flavonoid biosynthesis were validated for their expression profile (Fig. 6 and Supplementary Fig. 6). Analysis suggested that number of genes involved in the aromatic amino acid biosynthesis such as prephenate dehydratase up-regulated in both, leaf and fruit, tissue. 1-aminocyclopropane-1-carboxylic acid synthase (ACC synthase), an enzyme involved in committed and rate-limiting step in ethylene biosynthesis^44^,^45^ also up-regulated in both, leaf and fruit, tissue. The fold expression of ACC synthase is higher in fruit (100- fold) than leaf tissue (2- fold). The up-regulated expression of aquaporins is noticed in fruit tissue while it is not up-regulated significantly in leaf tissue. Aquaporins also known as water channels are integral membrane proteins which conduct only water molecules in and out of the cell and prevents the passage of ions and other solutes^46^. Expression of auxin efflux carrier family protein which regulates auxin transports in the plant cells was also validated for modulated expression due in *AtMYB12* expressing transgenic plants. Modulated expression of members of GST gene family which are differentially regulated in both, leaves and fruit, tissues and involved in the intracellular binding and stabilization of flavonoids^47^,^48^ was also validated through qRT-PCR.

### Significantly modulated pathways in *AtMYB12*-expressing tomato transgenic lines

In general, most of the genes belonging to the phenylpropanoid pathway leading to flavonol biosynthesis were up-regulated in both, leaf and fruit, tissues of transgenic tomato lines as compared to the similar tissues from WT plants (Supplementary Fig. 7a). Apart from phenylpropanoid pathway, certain genes putatively encoding glycosyltransferases (GTs) were found to be differentially regulated in *AtMYB12*- expressing transgenic line (Supplementary Fig. 7b). Out of these, few GTs exhibiting homology to flavonoid specific glycosyltransferases were up-regulated in fruit tissue of transgenic line in comparison to WT plants (Supplementary Fig. 7b). Another interesting observation of microarray analysis was modulation in expression of genes involved in phytohormone biosynthesis and signaling in *AtMYB12*-expressing transgenic plants. However, a differential modulation was noticed for most of such genes in leaves and fruits from transgenic plants with fruits displaying major modulation. These genes included those involved in ethylene biosynthesis and signaling, ABA, auxin and GA signaling (Supplementary Fig. 7c). The pathway analysis (Fig. 5b and supplementary Fig. 5b) also showed modulation in various networks involving these signaling processes.

Certain genes putatively involved in amino acid biosynthesis were found to be differentially regulated in leaf and fruit tissue of *AtMYB12*-expressing transgenic plant as compared to tissues from WT plant (Supplementary Fig. 7d). The most notable among them were those involved in aromatic amino acid biosynthesis including up-regulation of prephanate dehydratase and chorismate mutase and down-regulation of dehydroquinate dehydratase/shikimate dehydrogenase, tryptophan synthase and isochorismate hydratase in comparison to WT plants. However, in leaves, out of these, only prephanate hydratase was up-regulated in case of *AtMYB12*-expressing transgenic plant. Similar modulation in expression of genes associated with aromatic amino acid pathway was reported in tobacco transgenic plants constitutively expressing *AtMYB12* gene^14^. Many genes involved in primary carbon metabolism such as carbohydrate metabolism, organic acid metabolism as well as lipid biosynthesis and degradation were differentially regulated in *AtMYB12*-expressing transgenic lines with major modulation in fruit tissue (Supplementary Fig. 8). Most notable differential expression was observed in ribulose bisphosphate carboxylase, aldolase, lipoxygenase, glyceraldehyde 3-phosphate dehydrogenase and malate dehydrogenase genes involved in primary carbon metabolism. We speculate that the modulation in expression of genes related to amino acid metabolism and other primary pathways might be due to elevated demand of C-source to support enhanced biosynthesis of flavonoids and other phenolics in transgenic tomato.

Many genes putatively involved in various other biological processes such as polyamine metabolism, carotenoid biosynthesis, cell wall biosynthesis and degradation were found to be differentially regulated in *AtMYB12*-expressing transgenic line. In addition, certain genes putatively encoding sugar transporter, ammonium transporter and aquaporins were also differentially regulated. Many genes involved in biotic and abiotic stress responses were observed to be differentially regulated in transgenic tomato lines both in leaf and fruit tissues. A set of genes putatively encoding glutathione-S transferase were also found to be differentially regulated by *AtMYB12* expression in tomato (Supplementary Fig. 7e). These results suggest that AtMYB12 expression modulate many processes apart from phenylpropanoid pathway.

## Discussion

Among different secondary plant products, a large number of flavonoids are health beneficial to mankind due to their antioxidant, anticancerous, antiangiogenic and antiosteoporotic activities. Despite several health benefits, most of the foods, consumed by human are deficient in these compounds due to tight temporal and spatial regulation of the genes committed to their biosynthesis. Of the different groups of secondary plant products, flavonoids are ubiquitous in occurrence; however, certain beneficial flavonoids are restricted to a particular group of plants. The spatial and temporal pattern of flavonoid accumulation in plants is defined by the genes encoding the enzymes of the pathway via regulatory proteins or transcription factors. It has been suggested that development of commonly consumed plant varieties rich in flavonoid content may be beneficial for the human health.

The AtMYB12 transcription factor transcriptionally activates the expression of a set of structural genes involved in flavonol biosynthesis in *Arabidopsis*^34^,^35^. The tomato fruits are deficient in flavonoids owing to the transcriptional regulation of genes involved in flavonoid biosynthesis^36^,^49^,^50^. The fruit specific expression of AtMYB12 in tomato led to the significant increase in content of flavonols and other phenolics in fruits accompanied with enhanced expression of genes involved in flavonol biosynthesis^29^. However, constitutive expression of AtMYB12 and its impact over the morphological and phytochemical attributes of tomato has not been studied so far. In addition, no detailed information is available related to pathways, other than phenylpropanoid pathway, which might be modulated in leaves and fruits of *AtMYB12*-expressing tomato. To develop this information, it was desirable to develop a detailed understanding of transcriptional changes due to AtMYB12 expression in tomato. Therefore, to address aforementioned questions, in the present study, transgenic tomato lines constitutively expressing AtMYB12 gene were developed and analyzed for modulation in phytochemical profile and genome-wide transcriptome in both fruit and leaf tissues. The constitutive expression of *AtMYB12* in tomato, led to enhanced polyphenol and flavonoid contents in both leaf and fruit tissues of tomato (Supplementary Fig. 2b and c). The increase in flavonoid content can be primarily attributed to the massive enhancement in flavonol sub-group of flavonoids. Apart from flavonols, the CGA content was enhanced in fruit and leaf tissues as compared to these tissues in WT plants. Similar results were reported in case of petal pigmentation of *AtMYB12*-expressing transgenic tobacco plants by various workers^14^,^29^. The microarray analysis suggested that the impact of *AtMYB12* expression is not restricted to only genes of flavonoid biosynthesis but also to the genes belonging to other pathways such as amino acid biosynthesis, hormone biosynthesis, signaling and primary carbon metabolism (Supplementary Fig. 7 and 8). Similar large scale transcriptomic changes have been reported in tomato mutants containing high anthocyanin content^51^ and in *AtMYB12*-expressing transgenic tobacco plants^14^. A flavonoid deficient y mutant of tomato, exhibiting down-regulated expression of *SlMYB12*, an ortholog of *AtMYB12* also exhibited similar transcriptional response^52^.

In this study, expression analysis of the genes involved in phenylpropanoid pathway indicated enhanced expression of various structural genes involved in flavonol biosynthesis (Fig. 4 and Supplementary Fig. 3). However, interestingly, in microarray analysis, certain probe sets annotated as putative genes involved in flavonol biosynthesis such as CHS and F3′H were found to be down-regulated in AtMYB12-expressing tomato plants. Also, the expression of various putative structural genes leading to flavonol biosynthesis was not uniformly regulated in the two tissues analyzed. The analysis of recently released tomato genome database suggests the presence of multiple paralogs for various structural genes of phenylpropanoid and flavonoid pathway. The different paralogs might be regulated differentially by tissue specific and other exogenous and endogenous cues. Notably, the tomato gene chip, employed in present study represents information about 9200 transcripts and it seems that information about the certain paralogs of various genes might not be present on available tomato gene chip. Together, these facts explain the discrepancies associated with expression profile of phenylpropanoid pathway in transgenic and WT tomato studied in present work.

Microarray analysis also suggest that among various differentially regulated GST genes in transgenic tomato, one GST (sol genome locus id, Solyc10 g084400.1.1 and Arabidopsis hit AT5G02790.1) is homologous to the lambda classes of Arabidopsis GSTs. This class of GSTs have been reported to utilize flavonol backbone as ligand and suggested to play a role in maintaining the pool of flavonols^53^. While conclusive in planta function of GST proteins in flavonol biosynthesis and accumulation awaits further experimentation, the up-regulation of the gene encoding this class of GST provide further evidence for involvement of GST protein in flavonol accumulation.

The primary C-source for flavonoid pathway is present in the form of phenylalanine and malony CoA. Enhancement in biosynthesis of flavonoid might demand additional supply of these two molecules as substrate flux. The plastid localized shikimic acid pathway is responsible for biosynthesis of chorismate, a central precursor for the biosynthesis of aromatic amino acids including phenylalanine^54^. In addition, the biosynthesis of malonyl Co-A required for flavonoid biosynthesis as well as fatty acid biosynthesis is dependent on availability of acetyl Co-A, another central molecule in primary C metabolism^55^. A set of genes putatively encoding enzymes of phenylanine biosynthesis from chorismate such as chorismate mutase, prephanate dehydratase, arogenate dehydrogenase were found to be up-regulated in transgenic lines and might be involved in providing additional demand of phenylalanine for flavonoid biosynthesis. In addition, few genes putatively encoding enzymes of tryptophan biosynthesis were also down-regulated suggesting preferential biosynthesis of phenylalanine from a common precursor chorismate. However, interestingly certain genes putatively encoding enzymes of shikimic acid pathway were down-regulated in AtMYB12-expressing tomato transgenic lines that suggest complex regulation of chorismate biosynthesis under enhanced demand of phenylalanine.

The shikimic acid pathway is directly linked to the central C metabolism including glycolysis and oxidative pentose phosphate pathway for the substrate flux^54^. Many genes putatively involved in primary carbon metabolism such as those associated with calvin cycle, organic acid and lipid metabolism are modulated in their expression in *AtMYB12*-expressing transgenic tomato lines. These results suggest a major modulation of primary metabolism including carbohydrate and lipid metabolism to allow generation of substrate flux for flavonoid biosynthesis via other intermediate pathways. Such an apparent metabolic reprogramming should involve efficient channeling of substrates among various subcellular compartments. This cannot be ruled out as genes encoding certain putative sugar and ammonium transporters were also differentially regulated in *AtMYB12*-expressing transgenic tomato plants. The future work should involve high-throughput metabolomics and transcriptomics studies to decipher gene to metabolite relations for efficient development of models for substrate channeling towards flavonoid biosynthesis. Our analysis suggest differential expression of a large number of genes in the tomato transgenic plants and leads to open area of research to identify the direct targets of AtMYB12 by using appropriate inducible promoters.

In present work, a major impact of AtMYB12 expression over fruit tissue as compared to leaves of tomato has been reported. The tomato fruits are partially autotrophic in the beginning and acquire a status of a heterotopic entity as they get mature^56^. Therefore, the differential physiological and metabolic status of two tissues should be responsible for differential modulation of expression of genes in the two tissues. Many genes involved in stress response were observed to differentially regulated possibly due to the diversion of substrate flux from vital primary metabolism towards defense related secondary metabolism. In addition, flavonols act as signaling molecules and could be responsible for initiation of signaling cascades culminating into stress related response. Many genes involved in hormone biosynthesis and signaling were found to be differentially regulated in transgenic tomato. The enhanced accumulation of flavonoids at the expense of primary metabolites possibly acts as a signal for modulation of expression of these genes. Also, the modulation of various genes associated with phytohormone signaling could be a mechanism to optimize plant development and metabolism through metabolic reprogramming under the conditions of enhanced biosynthesis of secondary plant products. Recently, the role of phytohormone cross-talk in regulation of plant metabolism and growth via metabolic reprogramming has been established^57^.

In this study, we conclude that AtMYB12 expression leads to a large scale modulation in transcriptome of tomato that might be responsible for major metabolic reprogramming to favour the high level accumulation of flavonols and other phenolics. The present work demonstrates the flexibility of plant metabolism that can allow diversion of substrate flux towards health beneficial secondary metabolism. As most of the metabolic pathways are interconnected and their regulation is complex, the perturbation in one metabolic pathway, as in case of flavonoid biosynthesis in AtMYB12 transgenic tomato, can lead to dramatic changes in genome-wide transcriptome. With the availability of completely sequenced tomato genome, it will be easier to study the intricacies of networking of metabolic pathways and role of flavonoids as potential endogenous signaling molecules.

## Methods

### Plant material and growth condition

*Solanum lycopersicum* var. Pusa early dwarf (tomato) has been used in this study for raising the transgenic plants. Tomato plants were grown in glass house at 22 °C ± 2 °C and 16 h/8 h light-dark photoperiods. Samples were frozen in liquid nitrogen and kept in −80 °C deep freezer until further use. The experiment was performed by using three independent replicates.

### Tomato transformation and selection of transgenic lines

Pant expression construct carrying *AtMYB12* under control of CaMV35S promoter in plasmid pBI121 was developed as previously reported^14^,^22^. Construct carrying *AtMYB12* was transformed in *Agrobacterium* by the method of freeze and thaw^58^ was used for tomato transformation as per standard protocol^59^ with slight modifications. The hardened putative transgenics were shifted to glass house. The presence of transgene was confirmed by PCR analysis using vector specific forward and gene specific reverse primer (Supplementary Table S5). Seeds obtained from the transformants were grown on half strength MS media^60^ supplemented with 200 mg/l kanamycin for selection and surviving seedlings were grown in glass house to get next generation (T1). Out of 15 independent transgenic tomato lines, 3 were selected and used for further analysis. Transgenic lines were grown subsequently to get homozygous lines.

### Phytochemical analysis

Analysis of total anthocyanin, polyphenol, flavonoid and antioxidant activity as well as HPLC analysis was carried out essentially as previously described^61^,^62^,^63^.

### Genome wide expression analysis

Total RNA was extracted from young leaves and red ripe fruits of tomato using the Qiagen RNeasy Plant Maxi kit (Qiagen, MD, USA). Yield and RNA purity were determined spectrophotometrically (NanoDrop, Wilmington, DE, USA) and by agarose gel electrophoresis. The microarray was performed using one-cycle target labelling and control reagents (Affymetrix, USA) with 5 μg RNAand Affymetrix Gene Chip Tomato Genome Arrays. Target preparation, hybridization to arrays, washing, staining, scanning and analysis was carried out according to the manufacturer’s instructions (Affymetrix, USA) and Chakrabarty *et al.*, (2009)^64^. The Affymetrix microarray chips were normalized with GCRMA method for all replicates using R-bioconductor 2.15. The normalized values were used to compute contrast and get the fold change value each for tomato leaf and fruit between WT and transgenic plants expressing *AtMYB12*. The probe set ids which showed >2 fold change in transgenic lines in comparison to WT were considered significant and used for further analysis. The consensus sequences of all the probe set ids present on the tomato chip were downloaded from Affymetrix (http://www.affymetrix.com/support/technical/byproduct.affx?product=tomato) and were annotated with the tomato protein database (http://solgenomics.net/organism/Solanum_lycopersicum/genome, version ITAG 2.3) using BLASTX with e-value <10^−5^ to get corresponding tomato protein ids. The Arabidopsis ids for the corresponding tomato probe set ids were also retrieved from Plexdb online database (http://www.plexdb.org/).

GO annotation was retrieved for the up- and down-regulated genes from agriGo using singular enrichment analysis. The network analysis was done to study the network between cell processes, proteins and the functional classes using Pathway Studio software 9.0 with resnet 4.0 databases.

### Pathway analysis

All the genes identified in the microarray analysis using the combined criterion of 2-fold or greater change and a P value <0.05 in the t-tests were used to generate the cellular-function pathways and interactomes using the software Pathway Studio (Ariadne Genomics, MD, USA). Pathways were generated for the genes modulated by *AtMYB12* transcription factor.

### Gene expression analysis

For Real Time PCR, the reaction was run in triplicates for each samples and the data was analyzed by the mean of triplicates. SYBR green was used as a fluorescent indicator dye in all real time reactions (Applied Biosystems, USA). The amount of cDNA was normalized by using amplification of internal constitutive genes. The real time reaction was set up in triplicates in a final volume of 20 μl as follows, 1 μl of cDNA, SYBR Green Dye (2X) 10 μl, Forward and reverse (5 pmol each) 1 μl and sterile water. The general steps performed during real-time PCR experiment were as follows- step 1 50 ^°^C, 2 min, step 2 95 ^°^C, 10 min, step 3 (95 ^°^C 15 sec 60 ^°^C 1 min) x 40 cycles. List of oligonucleotides used for the real time analysis id provided in Supplementary Table S1. Data from q PCR amplification was analyzed using comparative Ct (2-∆∆ct) method^65^. The Ct values from control and experimental samples were plotted on excel data sheet and ∆Ct were calculated. Finally -∆∆Ct was calculated. Fold change in expression was calculated as (2-∆∆ct).

### Statistical analysis

All experiments were repeated three times with at least three biological and three technical replicates per treatment. Bars represent SD of means. *, **, and *** indicate values that differ significantly from the control at P < 0.05, < 0.01, and P < 0.001, respectively, according to Student’s paired t-test. All data are expressed as mean ± Standard Deviation.

## Additional Information

**How to cite this article**: Pandey, A. *et al.* *AtMYB12* expression in tomato leads to large scale differential modulation in transcriptome and flavonoid content in leaf and fruit tissues. *Sci. Rep.* **5**, 12412; doi: 10.1038/srep12412 (2015).

## Supplementary Material

Supplementary Information

## Figures and Tables

**Figure 1:**
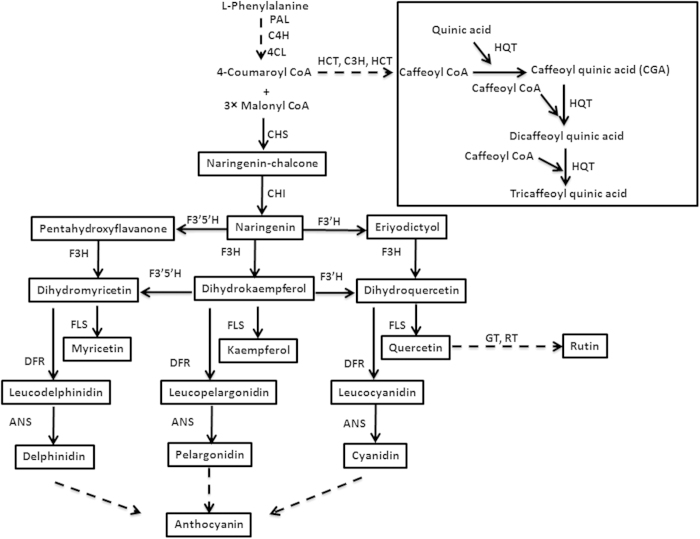
Flavonoid biosynthetic pathway. CHS, chalcone synthase; CHI, chalcone isomerase; C4H cinnamate 4-hydroxylase; 4CL, 4-coumaroyl CoA ligase; DFR, dihydroflavanol 4-reductase; F3H, flavanone 3-hydroxylase; F3′H, flavonoid 3′-hydroxylase; F3′5′H, flavonoid 3′5′ hydroxylase; FLS, flavonol synthase; GT, glucosyltransferase; PAL, phenylalanine ammonia lyase; RT, rhamnosyltransferase.
