# Supplementary material for: AtMYB12 expression in tomato leads to large scale differential modulation in transcriptome and flavonoid content in leaf and fruit tissues
Source: Sci Rep. 2015 Jul 24;5:12412. doi: 10.1038/srep12412 (PMC4513303; doi:10.1038/srep12412)
Supplement: Supplementary Information [file srep12412-s1.pdf]

Supplementary Data

AtMYB12 expression in tomato leads to large scale differential modulation in transcriptome and flavonoid content in leaf and fruit tissues

Ashutosh Pandey[1,$,#], Prashant Misra[1,$,#], Dharmendra Choudhary[2], Reena Yadav[1], Ridhi Goel[1], Sweta Bhambhani[1], Indraneel Sanyal[1], Ritu Trivedi[2,*], Prabodh Kumar Trivedi[1,*]

[1]Council of Scientific and Industrial Research-National Botanical Research Institute (CSIR-NBRI), Rana Pratap Marg, Lucknow-226 001, INDIA

[2]CSIR-Central Drug Research Institute (CSIR-CDRI), Endocrinology Division, Jankipuram Extension, Sitapur Road, Lucknow-226021, INDIA

[$]Present address (AP): National Agri-Food Biotechnology Institute (NABI), Mohali-160071, Punjab, INDIA

[$]Present address (PM): CSIR-Indian Institute of Integrative Medicine (IIIM), Canal Road, Jammu-180001, INDIA

#: Contributed equally to this study

*Authors for correspondence

PKT: prabodht@hotmail.com, prabodht@nbri.res.in

RT: ritu_trivedi@nbri.res.in

Running title: *AtMYB12* differentially modulates flavonoid biosynthesis in tomato fruit and leaf

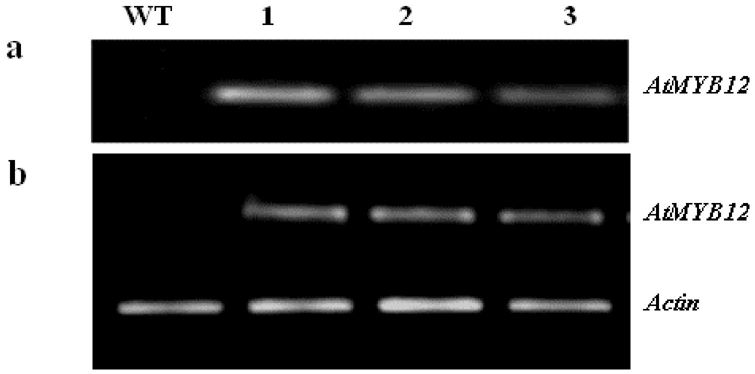

Supplementary Figure 1. Molecular characterization of transgenic lines. (a) Confirmation of the transgene by PCR amplification of genomic DNA with CaMV35S forward and gene specific reverse primers. (b) Expression analysis of transgene through semiquantitative RT-PCR using RNA from leaves.

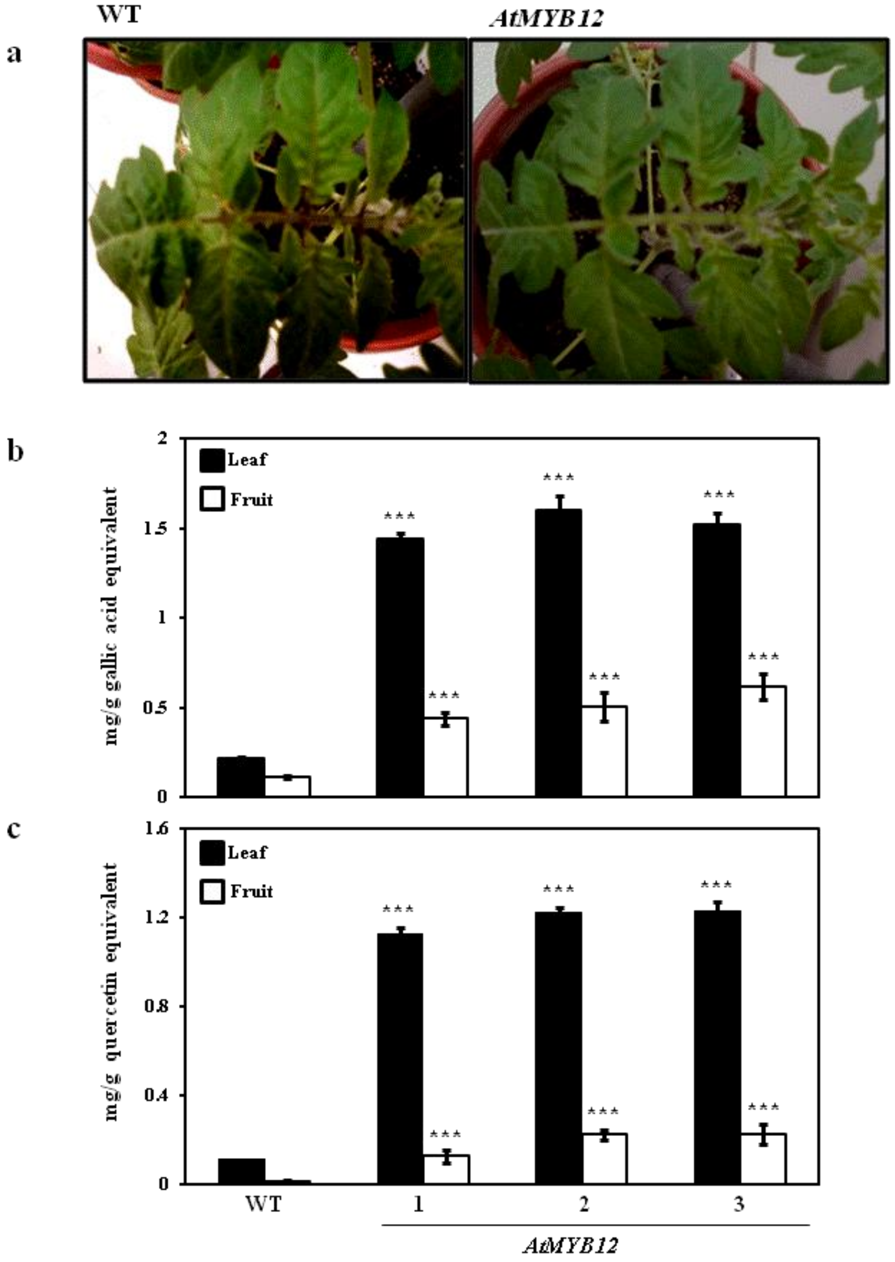

Supplementary Figure 2. Effect of *AtMYB12* expression on anthocyanin, polyphenol and flavonoid content in transgenic tomato lines as compared to WT. (a) Reduction in anthocyanin pigmentaion on stem and petiole in tomato transgenic line in comparison to wild type (WT) leaves. (b) Quantification of total polyphenolic content using methanolic extracts. Quantification has been carried out by gallic acid equivalent. (c) Quantification of total flavonoid content using methanolic extracts by quercetin equivalent. 1, 2 and 3 represent independent tomato transgenic lines expressing *AtMYB12*.

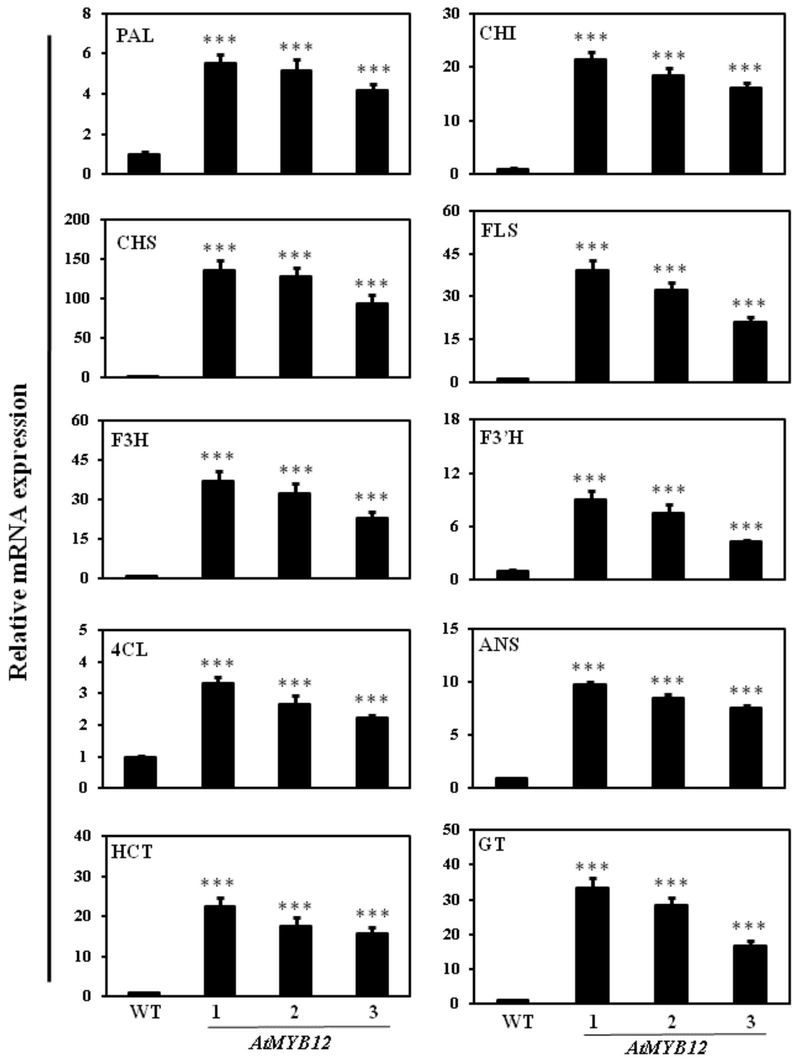

Supplementary Figure 3. Quantitative expression analysis of the structural genes up-regulated in leaves of *AtMYB12*-expressing tomato transgenic lines. Expression of structural genes of phenylpropanoid pathway/flavonoid pathway in leaves of the WT and transgenic tomato lines. 1, 2 and 3 represent transgenic line 1, line 2 and line 3, respectively. PAL, Phenylalanine amonia lyase; CHI, Chalcone isomerase; CHS, Chalcone synthase; FLS, Flavonol synthase; F3H, Flavonone-3-hydroxylase; F3'H, flavonoid 3'-hydroxylase; 4CL, 4-coumaroyl CoA ligase; ANS, Anthocyanidine synthase HCT, Hydroxycinnamoyl transferase; GT, Glucosyltransferase.

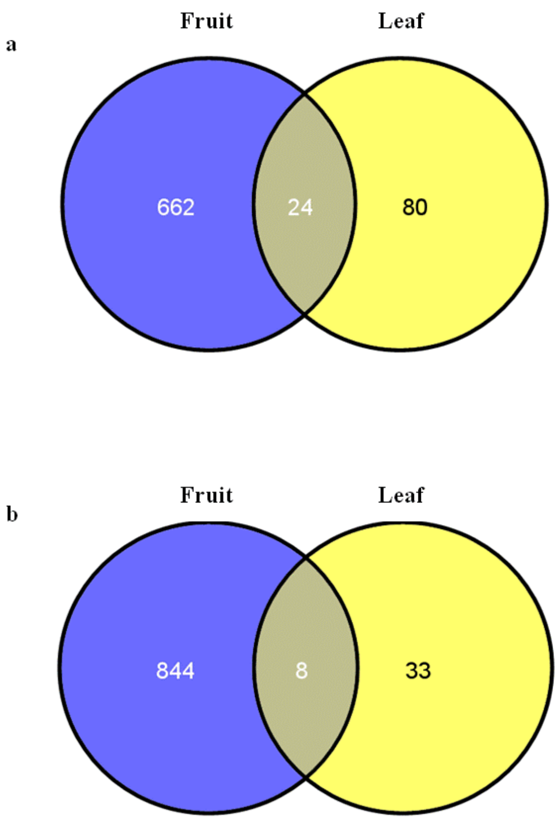

Supplementary Figure 4. Distribution of common and unique genes with modulated expression among leaves and fruits of transgenic and WT tomato plants. (a) up-regulated unigenes and (b) down-regulated unigenes.

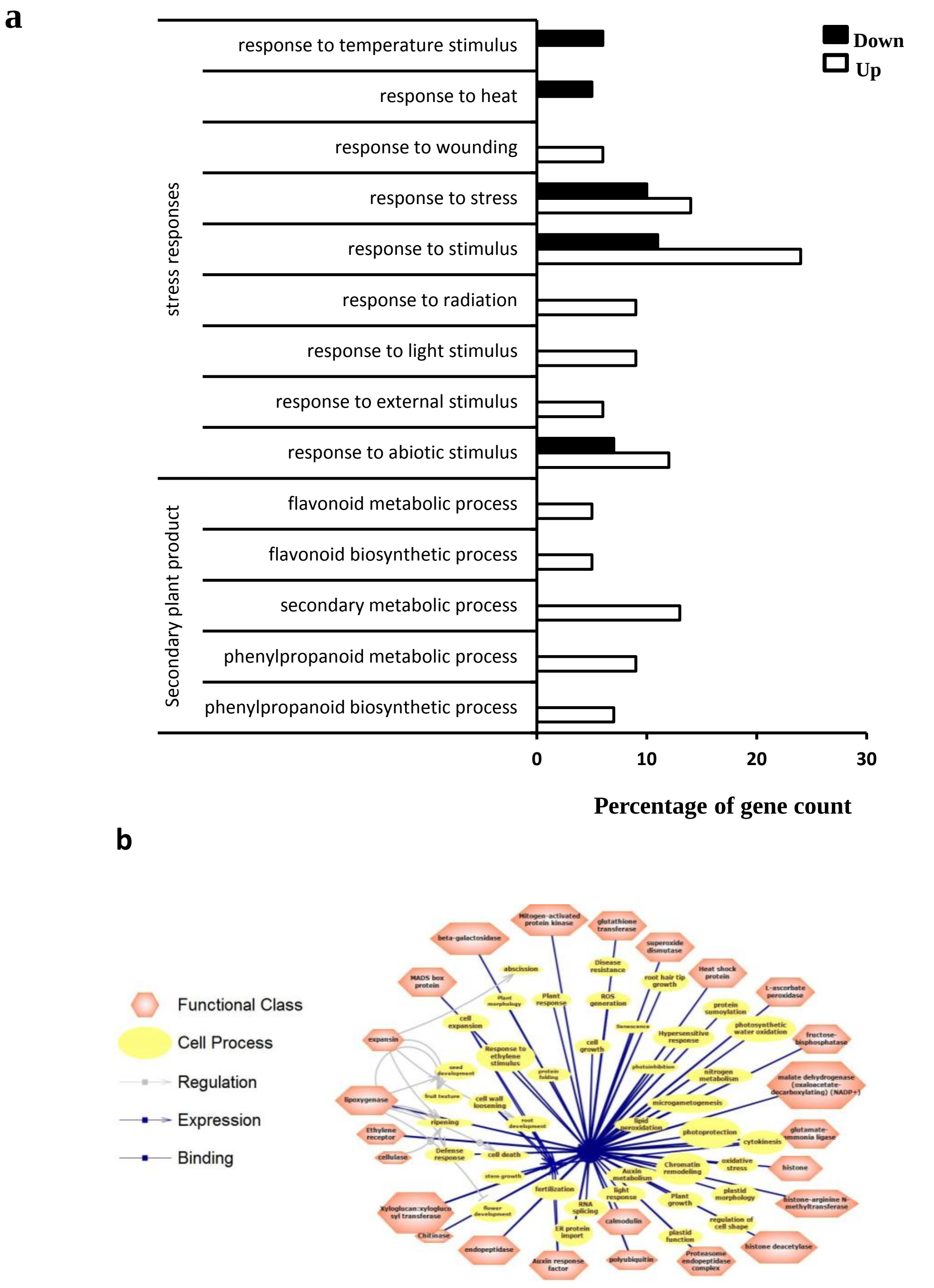

Supplementary Figure 5. Gene ontology of differentially regulated genes and functional network predicted. (a) *Solanum lycopersicum* homologues of the differentially-regulated genes in *AtMYB12*-expressing tomato leaves were identified and grouped with respect to their predicted role in different processes using agriGO (level 4). (b) *Solanum lycopersicum* homologues of the differentially expressed genes in *AtMYB12*-expressing tomato were identified to construct interactive network using Pathway Studio (Ariadne Genomics, USA).

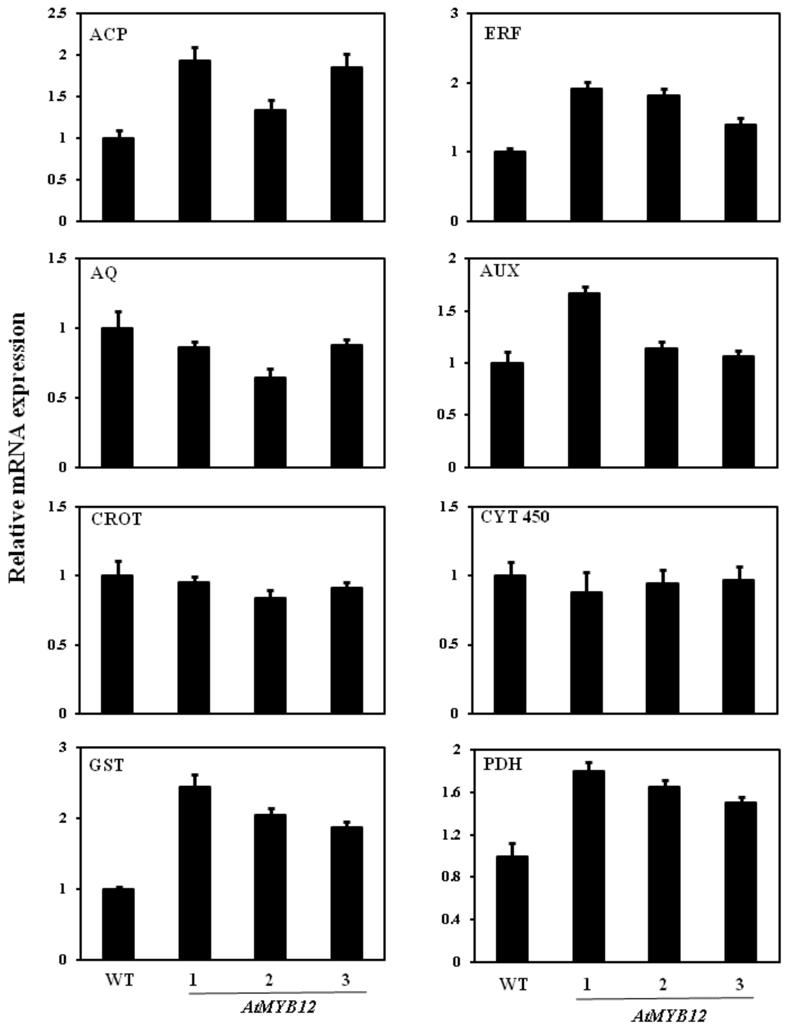

Supplementary Figure 6. Validation of differential expression of genes involved in different processes in leaves of *AtMYB12*-expressing tomato transgenic lines. 1, 2 and 3 represent line 1, line 2 and line 3 respectively.ACP-1-aminocyclopropane-1-carboxylate synthase (Solyc08g081550.2.1); AQ, Aquaporin (Solyc12g044330.1.1); AUX, Auxin efflux carrier family protein (Solyc02g082450.2.1); CROT, Crotenase (Solyc12g011160.1.1); CYT450, Cytochrome P450 (Solyc10g083700.2.1); ERF, Ethylene-responsive transcription factor 4 (Solyc07g053740.1.1); GST, Glutathione S-transferase (Solyc10g084400.1.1); PDH, Prephenate dehydratase (Solyc06g074530.1.1).

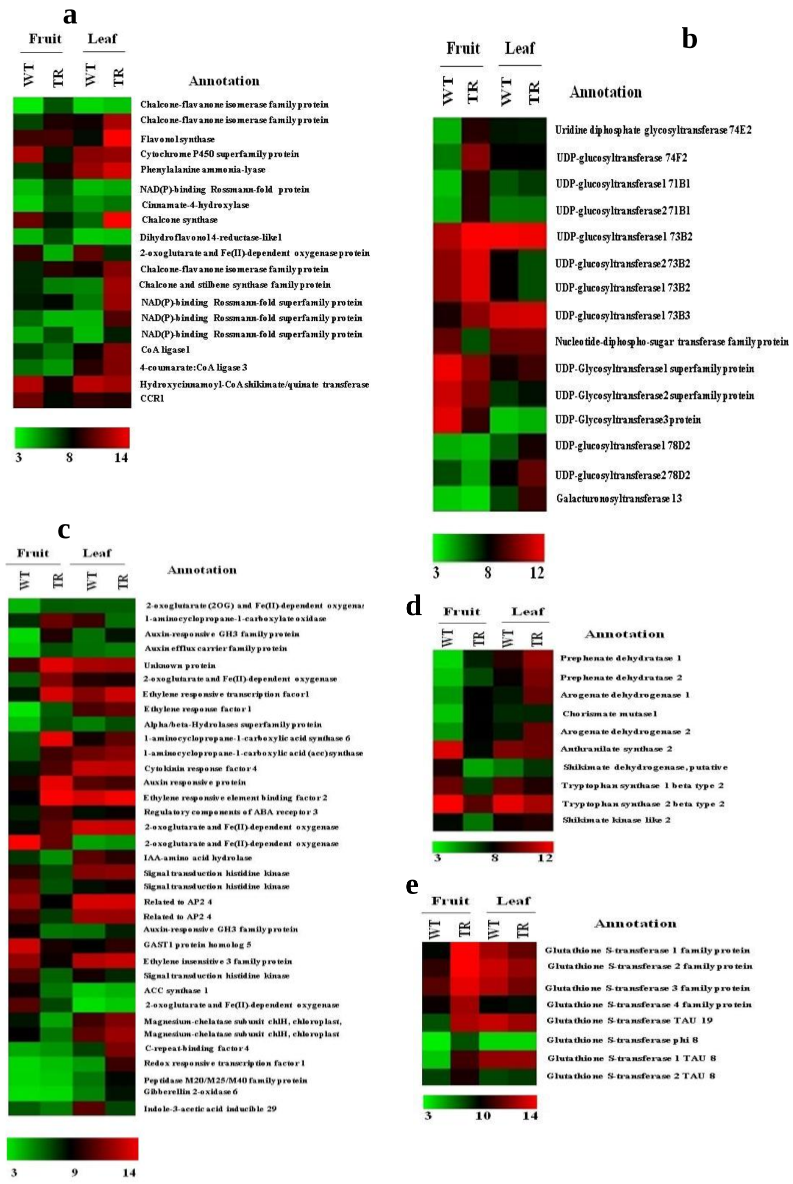

Supplementary Figure 7. Differential expression of genes in leaves and fruits of tomato plants. (a) Differential expression of genes involved in flavonoid biosynthesis. (b) Differential expression of genes encoding glycosyltransferases. (c) Differential expression of genes encoding involved in phytohormone biosynthesis, signalling and response. (d) Differential expression of genes involved in aromatic amino acid biosynthesis. (e) Differential expression of genes involved in glutathione S transferase. The locus ids of various regulatory genes were searched from microarray data. These probe set ids were used for annotation using different databases. Heat maps denoting digital expression by using MeV version 4.3 program. WT and TR represent wild type and transgenic line.

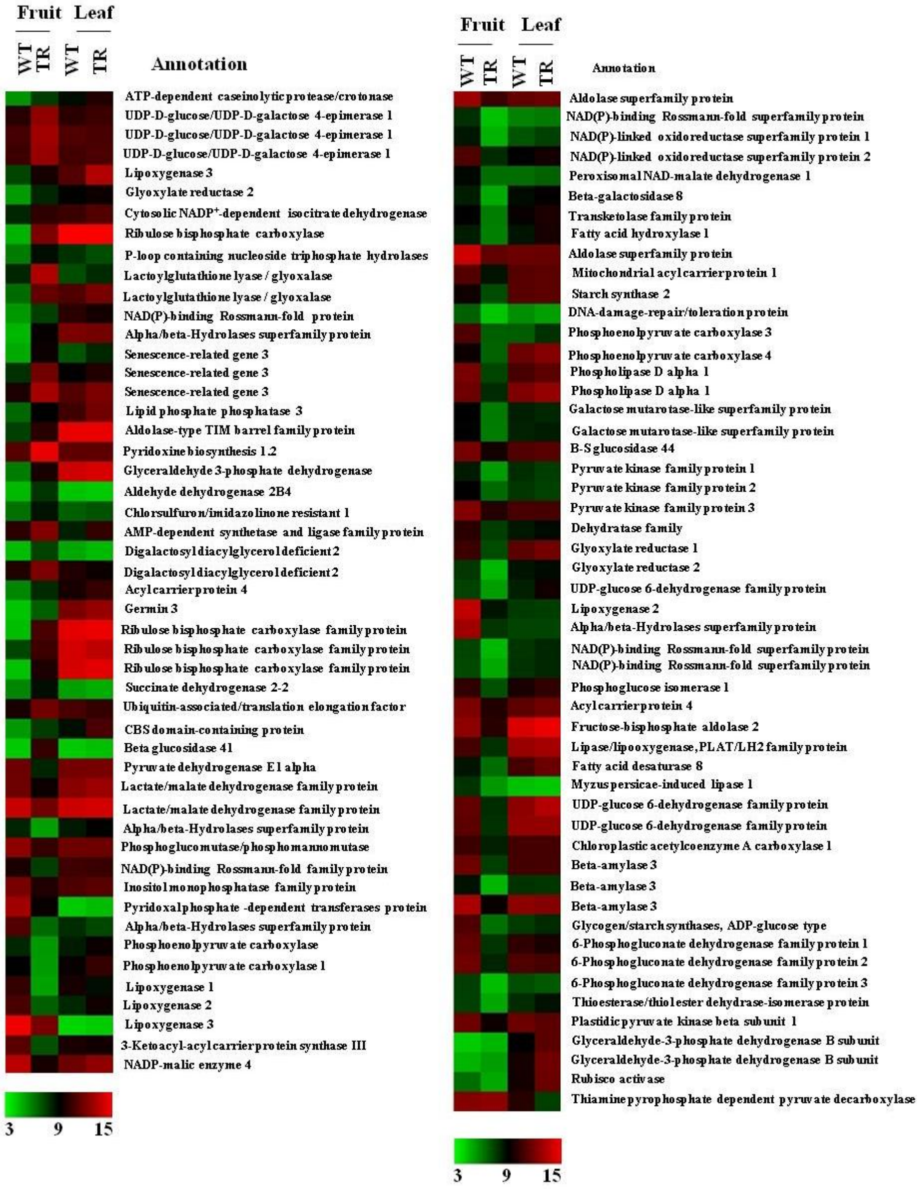

Supplementary Figure 8. Differential expression of genes encoding enzymes involved in carbohydrate, lipid and organic acid metabolism in leaves and fruits of tomato plants. The locus ids of various regulatory genes were searched from microarray data. These probe set ids were used for annotation using different databases. Heat maps denoting digital expression by using MeV version 4.3 program. WT and TR represent wild type and transgenic line.

Table 1: List of up-regulated (≥ two-fold) genes in leaves of *AtMYB12*-expressing tomato lines

S.No	Locus ID	SOLGEN locus id	Annotation in SOLGEN
1	Les.3649.1.S1_at	Solyc05g053550.2.1	Chalcone synthase
2	Les.3650.1.S1_at	Solyc09g091510.2.1	Chalcone synthase
3	Les.3085.1.S1_at	Solyc11g013110.1.1	Anthocyanidin synthase
4	Les.2278.1.S1_at	Solyc02g083860.2.1	Flavanone 3-hydroxylase
5	LesAffx.34276.2.S1_at	Solyc02g089770.2.1	Dihydroflavonol-4-reductase
6	LesAffx.34276.1.S1_at	Solyc02g089770.2.1	Dihydroflavonol-4-reductase
7	LesAffx.70635.1.S1_at	Solyc01g108240.2.1	Ethylene responsive transcription factor 2b
8	LesAffx.58308.1.S1_at	Solyc08g007820.1.1	Ethylene-responsive transcription factor 10
9	LesAffx.68320.1.S1_at	Solyc05g052240.2.1	Chalcone--flavonone isomerase
10	LesAffx.34276.2.A1_at	Solyc02g089770.2.1	Dihydroflavonol-4-reductase
11	LesAffx.47885.1.S1_at	Solyc12g094520.1.1	4-coumarate-coa ligase
12	LesAffx.17107.1.A1_at	Solyc02g088630.2.1	Glycosyltransferase
13	LesAffx.15067.1.S1_at	Solyc05g006220.2.1	IAA-amino acid hydrolase
14	LesAffx.6614.2.S1_at	Solyc07g048060.1.1	Auxin-induced protein-like
15	LesAffx.63776.1.S1_at	Solyc10g083440.1.1	UDP flavonoid 3-O-glucosyltransferase (Fragment)
16	LesAffx.12150.1.A1_at	Solyc03g097170.2.1	Cinnamoyl-CoA reductase-like protein
17	LesAffx.70136.2.S1_at	Solyc04g079050.1.1	UDP-glucosyltransferase
18	Les.5177.1.S1_at	Solyc02g064830.2.1	Indole-3-acetic acid-amido synthetase GH3.8
19	LesAffx.63587.1.S1_at	Solyc04g051360.2.1	Ethylene responsive transcription factor 2b
20	Les.5601.1.S1_at	Solyc02g071380.2.1	1-aminocyclopropane-1-carboxylate oxidase 3
21	Les.5848.1.A1_at	Solyc03g097030.2.1	4-coumarate CoA ligase
22	LesAffx.63544.1.S1_at	Solyc08g080290.2.1	Ethylene-responsive transcription factor 1
23	RPTR-Les-U57609-2_s_at		No Hit
24	Les.561.1.A1_at		No Hit
25	LesAffx.30832.1.S1_at	Solyc08g036640.2.1	Protein TIFY 5A
26	Les.1610.1.S1_at	Solyc02g089620.2.1	Proline dehydrogenase
27	Les.3040.1.S1_at	Solyc01g104110.2.1	Legumin 11S-globulin
28	Les.5233.1.S1_at	Solyc03g123620.2.1	Pectinesterase
29	LesAffx.61398.1.S1_at	Solyc06g060570.2.1	Ovarian cancer-associated gene 2 protein homolog
30	Les.3034.1.S1_at	Solyc11g020960.1.1	Proteinase inhibitor II
31	Les.4618.1.S1_at	Solyc04g025650.2.1	Monooxygenase FAD-binding
32	Les.4803.1.S1_at	Solyc02g089630.2.1	Proline dehydrogenase
33	Les.2294.2.A1_a_at		No Hit
34	Les.5056.1.S1_x_at	Solyc11g071760.1.1	Calmodulin-like protein
35	LesAffx.4617.1.A1_at	Solyc08g079090.2.1	Laccase-22
36	Les.13.1.S1_at	Solyc04g079730.1.1	cytochrome P450"
37	Les.5056.1.S1_a_at	Solyc11g071760.1.1	Calmodulin-like protein
38	Les.4957.1.S1_at	Solyc09g007940.2.1	Adenosine kinase
39	LesAffx.22830.1.S1_at	Solyc02g087210.2.1	Zinc finger AN1 domain-containing stress-associated protein 12
40	Les.3509.1.S1_at	Solyc03g045070.1.1	Ammonium transporter
41	Les.4459.1.S1_s_at		No Hit
42	Les.213.1.S1_at	Solyc06g005470.2.1	Metallothionein-like protein type 2
43	LesAffx.3253.1.S1_at		No Hit
44	Les.1968.1.A1_at		No Hit
45	Les.2813.1.S1_at	Solyc06g074670.2.1	Bifunctional polymyxin resistance arnA protein
46	Les.4530.1.S1_at	Solyc07g055990.2.1	Xyloglucan endotransglucosylase/hydrolase 7
47	LesAffx.70534.1.S1_at	Solyc01g079200.2.1	Gibberellin 2-oxidase
48	Les.975.1.S1_at	Solyc01g010660.2.1	Receptor-like protein kinase At3g21340
49	Les.2328.1.A1_at		No Hit
50	LesAffx.64357.1.S1_at	Solyc03g007770.2.1	C4-dicarboxylate transporter/malic acid transport family protein
51	Les.3594.1.S1_at		No Hit
52	Les.5927.1.S1_at	Solyc04g005480.1.1	Unknown Protein
53	Les.790.1.A1_at		No Hit
54	Les.4611.1.S1_at	Solyc03g008010.2.1	PPPDE peptidase domain-containing protein 1
55	Les.2325.1.S1_at	Solyc01g073640.2.1	Uncharacterized oxidoreductase Mb1385
56	LesAffx.26661.1.S1_at	Solyc08g077630.2.1	ATP binding / serine-threonine kinase
57	Les.3716.1.S1_at	Solyc10g052470.1.1	Myb family transcription factor (Fragment)
58	LesAffx.44987.1.S1_at	Solyc07g061990.2.1	Solanesyl diphosphate synthase
59	Les.124.1.S1_at	Solyc03g026280.2.1	CRT binding factor 2
60	LesAffx.28554.1.S1_at	Solyc04g077860.2.1	Hydrolase alpha/beta fold family protein expressed
61	Les.585.1.A1_at		No Hit
62	Les.4529.1.S1_at	Solyc02g091920.2.1	Xyloglucan endotransglucosylase/hydrolase 2
63	Les.5880.2.S1_at	Solyc03g082660.2.1	Major facilitator superfamily domain containing protein 5
64	Les.4493.3.S1_at	Solyc03g079880.2.1	Protease inhibitor/seed storage/lipid transfer protein family protein
65	LesAffx.64983.1.S1_at	Solyc04g051820.2.1	Os03g0169900 protein (Fragment)
66	LesAffx.58193.1.A1_at	Solyc01g065530.2.1	COBRA-like protein

67	Les.2003.1.A1_at		No Hit
68	Les.4353.1.S1_at	Solyc03g093130.2.1	Xyloglucan endotransglucosylase/hydrolase 9
69	LesAffx.57367.1.S1_at	Solyc05g008370.1.1	Ribose-5-phosphate isomerase
70	Les.3610.1.S1_at	Solyc02g032910.1.1	Glycine rich protein
71	LesAffx.3704.1.S1_at	Solyc01g080410.2.1	Peptide methionine sulfoxide reductase msrB
72	LesAffx.66458.1.S1_at	Solyc09g090600.2.1	Acid phosphatase-like protein
73	Les.4968.1.S1_s_at	Solyc04g008210.1.1	Xyloglucan endotransglucosylase/hydrolase 13
74	Les.3632.1.S1_at	Solyc03g122340.2.1	Lipoxygenase
75	LesAffx.54483.1.S1_at	Solyc06g084030.2.1	Methyltransferase like 7A
76	Les.3171.3.S1_a_at	Solyc03g031860.2.1	Phytoene synthase 1"
77	Les.2332.1.A1_at	Solyc09g005480.2.1	F-box family protein
78	LesAffx.66432.1.S1_at	Solyc02g094180.2.1	Peroxidase 1
79	LesAffx.71654.1.S1_at	Solyc04g074950.2.1	Ovarian cancer-associated gene 2 protein homolog
80	Les.5706.1.S1_at	Solyc04g080550.2.1	Phenylcoumaran benzylic ether reductase
81	Les.5038.1.S1_at	Solyc12g009000.1.1	Os07g0175100 protein (Fragment)
82	LesAffx.44987.3.S1_at	Solyc07g061990.2.1	Solanesyl diphosphate synthase
83	Les.612.1.S1_at	Solyc04g055260.2.1	Carboxyl methyltransferase
84	Les.3492.1.S1_at	Solyc01g091910.2.1	Phospholipase D
85	Les.3242.2.S1_at	Solyc12g094640.1.1	Glyceraldehyde-3-phosphate dehydrogenase B
86	Les.2633.1.A1_at		No Hit
87	LesAffx.62698.2.S1_at	Solyc03g025710.2.1	GCN5-related N-acetyltransferase
88	Les.2813.2.A1_at		No Hit
89	LesAffx.60586.1.S1_at	Solyc05g008870.2.1	Cytidyltransferase-related
90	LesAffx.58075.1.S1_at	Solyc06g050630.2.1	Prephenate dehydrogenase family protein
91	LesAffx.22898.1.S1_at		No Hit
92	LesAffx.3336.1.S1_at	Solyc02g078480.2.1	CBS domain containing protein
93	Les.3537.1.S1_at	Solyc04g008210.1.1	Xyloglucan endotransglucosylase/hydrolase 13
94	Les.612.2.A1_at	Solyc04g055260.2.1	Carboxyl methyltransferase
95	Les.4923.1.S1_at	Solyc10g005080.2.1	Late elongated hypocotyl and circadian clock associated-1-like"
96	LesAffx.1345.1.S1_at	Solyc06g068970.2.1	Conserved transmembrane protein
97	Les.3242.3.S1_at	Solyc12g094640.1.1	Glyceraldehyde-3-phosphate dehydrogenase B
98	Les.218.2.S1_at	Solyc02g080210.2.1	Pectinesterase
99	LesAffx.64062.1.S1_at	Solyc03g123410.1.1	Oxalate oxidase-like germin 171
100	LesAffx.71608.1.S1_at	Solyc02g091180.1.1	cDNA clone J100026I16 full insert sequence
101	Les.429.1.S1_at	Solyc07g056000.2.1	Xyloglucan endotransglucosylase/hydrolase 7
102	LesAffx.41518.1.S1_at	Solyc02g081600.2.1	Zinc transporter protein
103	Les.4868.1.S1_at	Solyc09g011080.2.1	Ribulose-1 5-bisphosphate carboxylase/oxygenase activase 1
104	LesAffx.66384.1.S1_at	Solyc06g072430.1.1	Bcl-2-associated athanogene-like protein

Table 2: List of down-regulated (≥ two-fold) genes in leaves of *AtMYB12*-expressing tomato lines

S.No.	Locus ID	SOLGEN locus id	Annotation in SOLGEN
1	LesAffx.52290.1.S1_at	Solyc03g111550.2.1	GDSL esterase/lipase At3g48460
2	Les.1219.1.A1_at		No Hit
3	Les.2350.3.A1_at		No Hit
4	Les.3436.2.S1_a_at	Solyc06g061230.2.1	Unknown Protein
5	Les.722.1.A1_at		No Hit
6	Les.2494.1.S1_at	Solyc02g067580.2.1	B12D-like protein
7	Les.1719.1.A1_at		No Hit
8	Les.129.1.S1_at	Solyc01g109140.2.1	Cytochrome P450"
9	Les.2350.1.S1_at	Solyc02g083250.2.1	Cellular retinaldehyde-binding/triple function C-terminal
10	Les.4307.1.S1_at	Solyc08g080670.1.1	Osmotin-like protein (Fragment)
11	LesAffx.57163.2.S1_at	Solyc10g076670.1.1	Anthocyanidin synthase
12	Les.840.1.A1_at		No Hit
13	Les.1596.1.A1_at		No Hit
14	LesAffx.44609.1.S1_at	Solyc09g008830.2.1	Os01g0318400 protein (Fragment)
15	Les.1735.1.A1_at		No Hit
16	Les.3408.1.S2_at		No Hit
17	LesAffx.24696.1.S1_at	Solyc08g062960.2.1	Heat stress transcription factor A3
18	Les.269.1.S1_at	Solyc03g082420.2.1	Heat shock protein
19	Les.2494.2.A1_at		No Hit
20	Les.3739.1.S1_at	Solyc11g020330.1.1	class IV heat shock protein
21	LesAffx.49935.1.S1_at	Solyc10g076510.1.1	Pyruvate decarboxylase
22	LesAffx.66253.1.S1_at	Solyc01g005760.2.1	LRR receptor-like serine/threonine-protein kinase, RLP"
23	Les.3726.1.S1_at	Solyc04g072160.2.1	Prostaglandin E synthase 3
24	LesAffx.26180.1.S1_at	Solyc03g043860.2.1	Nudix hydrolase 1
25	Les.195.1.S1_at	Solyc01g095330.2.1	Unknown Protein
26	Les.504.1.A1_at		No Hit
27	LesAffx.10596.1.S1_at	Solyc09g015020.1.1	class I heat shock protein 3
28	LesAffx.71065.1.S1_at	Solyc04g064870.2.1	Pathogenesis-related protein-like protein
29	Les.3397.1.S1_at	Solyc03g098720.2.1	Kunitz trypsin inhibitor
30	Les.5253.1.S1_at	Solyc06g005890.2.1	Unknown Protein
31	Les.4820.1.S1_x_at	Solyc00g071180.2.1	Cysteine proteinase inhibitor
32	Les.2063.1.A1_at		No Hit
33	Les.3677.1.S1_at	Solyc05g014280.2.1	Heat shock protein
34	LesAffx.18025.1.S1_at	Solyc08g006770.2.1	Anthocyanidin synthase (Fragment)
35	LesAffx.70264.1.S1_at	Solyc04g014480.2.1	class I heat shock protein 3
36	Les.5442.1.S1_at	Solyc08g021820.2.1	Auxin responsive protein
37	Les.3397.2.A1_at		No Hit
38	Les.5917.1.S1_at	Solyc07g026650.2.1	1-aminocyclopropane-1-carboxylate oxidase
39	Les.796.1.A1_at		No Hit
40	Les.4522.1.S1_at	Solyc11g066270.1.1	Xyloglucan endotransglucosylase/hydrolase 9
41	Les.3700.1.S1_at	Solyc07g008240.2.1	Non-symbiotic hemoglobin protein

Table 3: List of up-regulated (≥ two-fold) genes in fruits of *AtMYB12*-expressing tomato lines

S.No	Locus ID	SOLGEN locus id	Annotation in SOLGEN
1	Les.3017.2.S1_at	Solyc12g044330.1.1	Aquaporin
2	Les.1841.1.S1_at	Solyc08g081550.2.1	1-aminocyclopropane-1-carboxylate synthase
3	Les.4501.1.S1_at	Solyc09g011590.2.1	Glutathione S-transferase-like protein
4	Les.5177.1.S1_at	Solyc02g064830.2.1	Indole-3-acetic acid-amido synthetase GH3.8
5	LesAffx.15898.1.S1_at	Solyc08g006330.2.1	UDP-glucose salicylic acid glucosyltransferase
6	Les.3573.1.S1_at	Solyc05g052050.1.1	Ethylene responsive transcription factor 1a
7	LesAffx.70524.2.S1_at	Solyc12g014010.1.1	Glucosyltransferase
8	Les.3575.1.S1_at	Solyc02g077370.1.1	Ethylene-responsive transcription factor 2
9	Les.4412.1.A1_at	Solyc11g072110.1.1	1-AMINOCYCLOPROPANE-1-CARBOXYLATE OXIDASE-like protein
10	LesAffx.70524.1.S1_at	Solyc12g014010.1.1	Glucosyltransferase
11	Les.546.1.A1_at	Solyc02g093050.2.1	WRKY transcription factor 26
12	LesAffx.24192.1.S1_at	Solyc03g031450.2.1	BHLH transcription factor-like protein
13	LesAffx.15898.2.S1_at	Solyc08g006410.2.1	UDP-glucose glucosyltransferase
14	Les.1724.2.S1_at	Solyc10g084400.1.1	Glutathione S-transferase
15	LesAffx.58075.1.S1_at	Solyc06g050630.2.1	Prephenate dehydrogenase family protein
16	LesAffx.52437.1.S1_at	Solyc02g082450.2.1	Auxin efflux carrier family protein
17	Les.3415.2.S1_at	Solyc05g041200.2.1	4-hydroxyphenylpyruvate dioxygenase
18	LesAffx.8720.2.S1_at	Solyc10g083700.2.1	Cytochrome P450"
19	LesAffx.38821.1.S1_at	Solyc02g092860.2.1	Cytochrome P450"
20	Les.5571.1.S1_at	Solyc07g053740.1.1	Ethylene-responsive transcription factor 4
21	LesAffx.10955.2.S1_at	Solyc06g074530.1.1	Prephenate dehydratase
22	LesAffx.8720.1.S1_at	Solyc10g083690.2.1	Cytochrome P450"
23	Les.4299.1.S1_at	Solyc08g074630.1.1	Polyphenol oxidase
24	Les.3415.1.S1_at	Solyc05g041200.2.1	4-hydroxyphenylpyruvate dioxygenase
25	Les.131.1.S1_at	Solyc01g086680.2.1	Glutathione S-transferase
26	Les.3777.1.S1_at	Solyc01g107820.2.1	UDP-glucosyltransferase family 1 protein
27	LesAffx.17387.1.S1_at	Solyc09g010000.2.1	1-aminocyclopropane-1-carboxylate oxidase-like protein
28	LesAffx.68320.1.S1_at	Solyc05g052240.2.1	Chalcone--flavonone isomerase
29	Les.3574.1.S1_at	Solyc06g082590.1.1	Ethylene responsive transcription factor 1b
30	LesAffx.69088.1.S1_at	Solyc12g011160.1.1	Enoyl-CoA-hydratase
31	Les.4271.2.S1_at	Solyc09g007920.2.1	Phenylalanine ammonia-lyase
32	LesAffx.66380.1.S1_at	Solyc01g107830.2.1	UDP-glucosyltransferase family 1 protein
33	Les.5884.1.S1_at	Solyc03g098730.1.1	Kunitz trypsin inhibitor
34	Les.3358.3.S1_at		No Hit
35	Les.5052.1.S1_at	Solyc01g006050.2.1	Plant viral-response family protein
36	RPTR-Les-U57609-2_s_at		No Hit
37	Les.3217.3.S1_s_at	Solyc03g034220.2.1	Ribulose bisphosphate carboxylase small chain
38	Les.2876.2.S1_at	Solyc09g009100.2.1	Heat stress transcription factor A3
39	Les.2971.2.A1_at		No Hit
40	Les.5530.1.S1_at	Solyc10g086280.1.1	Heavy metal-associated domain containing protein expressed
41	Les.3021.1.S1_at	Solyc09g089510.2.1	Proteinase inhibitor I
42	Les.3519.1.S1_at	Solyc01g107730.2.1	Cyclin
43	Les.3217.2.S1_at		No Hit
44	Les.314.2.S1_at	Solyc10g055630.1.1	Aquaporin
45	Les.5847.1.S1_at	Solyc08g077910.2.1	Expansin-like protein
46	LesAffx.58041.1.A1_at	Solyc07g063880.2.1	Beta-glucosidase
47	Les.4345.2.A1_a_at	Solyc03g005780.1.1	Chlorophyll a-b binding protein 3C-like
48	Les.3621.1.S1_at	Solyc09g084470.2.1	Proteinase inhibitor I
49	Les.4345.2.A1_x_at	Solyc03g005780.1.1	Chlorophyll a-b binding protein 3C-like
50	Les.2332.1.A1_at	Solyc09g005480.2.1	F-box family protein
51	LesAffx.15004.1.S1_at	Solyc03g118060.2.1	Unknown Protein
52	Les.4701.1.S1_at	Solyc09g097960.2.1	Aldo/keto reductase family protein
53	Les.5597.1.S1_at	Solyc05g056400.2.1	Protein disulfide isomerase
54	Les.4041.1.S1_s_at	Solyc09g091110.2.1	Unknown Protein
55	Les.4345.1.S1_at	Solyc02g071030.1.1	Chlorophyll a/b binding protein
56	Les.4345.3.S1_x_at	Solyc02g071030.1.1	Chlorophyll a/b binding protein
57	LesAffx.59441.1.S1_at	Solyc01g090890.2.1	Xenotropic and polytropic retrovirus receptor
58	Les.4026.1.S1_at	Solyc01g080010.2.1	Xylanase inhibitor (Fragment)
59	Les.3741.1.S1_at	Solyc11g011340.1.1	Alcohol dehydrogenase
60	Les.314.1.A1_at	Solyc10g055630.1.1	Aquaporin
61	Les.2248.1.A1_at		No Hit
62	Les.2971.1.S1_at	Solyc09g083440.2.1	Proteinase inhibitor I
63	Les.3756.1.S1_a_at	Solyc11g021060.1.1	Proteinase inhibitor
64	LesAffx.44496.1.A1_at	Solyc00g171810.2.1	ATP synthase subunit a
65	Les.5056.1.S1_a_at	Solyc11g071760.1.1	Calmodulin-like protein
66	LesAffx.17773.1.S1_at	Solyc09g075890.2.1	Pyrimidine 5&apos-nucleotidase
67	Les.2886.3.S1_at		No Hit

68	Les.4041.1.S1_at		No Hit
69	LesAffx.51975.3.S1_at	Solyc00g019740.1.1	ATPase subunit 8
70	LesAffx.1881.1.S1_at	Solyc02g083790.2.1	Thaumatin-like protein
71	Les.376.1.S1_at	Solyc02g063150.2.1	Ribulose bisphosphate carboxylase small chain
72	LesAffx.22100.1.S1_at	Solyc07g055710.2.1	Heat stress transcription factor A3
73	LesAffx.24696.1.S1_at	Solyc08g062960.2.1	Heat stress transcription factor A3
74	LesAffx.62933.1.S1_at	Solyc02g087430.2.1	Inositol 1 4 5-trisphosphate 5-phosphatase-like protein
75	LesAffx.30145.1.S1_at	Solyc01g005470.2.1	Cell number regulator 10
76	Les.4428.2.S1_at	Solyc02g086820.2.1	Carbonic anhydrase
77	LesAffx.34336.1.S1_at	Solyc09g075950.1.1	Heat shock protein 1
78	LesAffx.69872.1.S1_at	Solyc01g073720.2.1	Unknown Protein
79	Les.4024.1.S1_at	Solyc05g032840.1.1	Unknown Protein
80	Les.2082.1.A1_at		No Hit
81	Les.3974.1.A1_at	Solyc07g007250.2.1	Metallocarboxypeptidase inhibitor
82	Les.3035.1.A1_at		No Hit
83	Les.1665.1.S1_at	Solyc03g116530.2.1	Glyoxalase/bleomycin resistance protein/dioxygenase
84	Les.4043.1.S1_at	Solyc09g091110.2.1	Unknown Protein
85	Les.2001.1.S1_s_at	Solyc10g086570.2.1	Palmitoyltransferase PFA4
86	LesAffx.66384.1.S1_at	Solyc06g072430.1.1	Bcl-2-associated athanogene-like protein
87	Les.1349.1.A1_at		No Hit
88	Les.3358.1.S1_at	Solyc10g054440.1.1	Arginine decarboxylase
89	Les.413.1.S1_s_at	Solyc07g056480.2.1	Glutathione S-transferase-like protein
90	Les.1675.1.S1_at	Solyc03g020060.2.1	Proteinase inhibitor II
91	LesAffx.63979.1.S1_at	Solyc10g051160.1.1	F-box protein PP2-B1
92	LesAffx.10313.1.A1_at	Solyc01g112220.2.1	Serine/threonine protein kinase-like
93	LesAffx.9007.1.S1_at	Solyc01g103590.2.1	Glyoxalase/bleomycin resistance protein/dioxygenase
94	Les.3286.1.S1_at	Solyc07g064600.2.1	Endoribonuclease L-PSP family protein
95	LesAffx.43628.1.S1_at	Solyc03g117590.2.1	Chaperone protein dnaJ
96	Les.3739.1.S1_at	Solyc11g020330.1.1	class IV heat shock protein
97	Les.2985.1.A1_at	Solyc04g040160.2.1	Pheophorbide a oxygenase
98	Les.3539.1.S1_at	Solyc04g009900.2.1	Calcium-dependent protein kinase 2
99	Les.3054.1.S1_at	Solyc12g011450.1.1	Chlorophyll a-b binding protein 13, chloroplastic
100	Les.13.1.S1_at	Solyc04g079730.1.1	cytochrome P450"
101	Les.5056.1.S1_x_at	Solyc11g071760.1.1	Calmodulin-like protein
102	Les.3757.1.S1_at	Solyc03g006490.2.1	Aluminum-induced protein-like
103	LesAffx.1091.1.S1_at	Solyc06g071060.1.1	Short-chain dehydrogenase/reductase family protein
104	LesAffx.49877.1.S1_at	Solyc08g079480.2.1	Unknown Protein
105	LesAffx.70522.1.S1_at	Solyc01g079940.2.1	Xylanase inhibitor (Fragment)
106	Les.4419.1.A1_at		No Hit
107	LesAffx.64902.2.S1_at	Solyc08g006150.2.1	ChaC cation transport regulator-like 1
108	Les.147.1.S1_at	Solyc12g006140.1.1	Chlorophyll a-b binding protein 37, chloroplastic
109	LesAffx.68801.1.S1_at	Solyc12g009610.1.1	TBC1 domain family member 15
110	LesAffx.70414.1.S1_at	Solyc01g067000.2.1	SRC2 homolog (Fragment)
111	Les.5362.1.S1_at	Solyc11g006010.1.1	DNA cross-link repair 1B-like protein
112	Les.2672.2.S1_at		No Hit
113	LesAffx.63739.1.S1_at	Solyc01g109720.2.1	Hydrolase-like
114	Les.1243.1.A1_at		No Hit
115	LesAffx.67427.1.S1_at	Solyc03g096770.1.1	Unknown Protein
116	Les.3358.1.S1_a_at		No Hit
117	Les.1606.1.S1_at	Solyc04g010250.2.1	Lipase-like protein
118	Les.5075.1.S1_at	Solyc07g065500.1.1	Nuclear transcription factor Y subunit B-3
119	Les.3740.1.S1_at	Solyc03g098790.1.1	Kunitz-type protease inhibitor
120	LesAffx.64902.3.S1_at	Solyc08g006150.2.1	ChaC cation transport regulator-like 1
121	LesAffx.58573.1.S1_a_at	Solyc03g116550.2.1	AT1G52630-like protein (Fragment)
122	LesAffx.50270.2.S1_at	Solyc02g082900.2.1	Strictosidine synthase family protein
123	Les.2886.2.S1_at	Solyc02g036370.2.1	MYB transcription factor (Fragment)
124	LesAffx.31298.1.S1_at	Solyc02g093600.2.1	class I heat shock protein
125	Les.2672.1.S1_s_at	Solyc06g062540.2.1	Phosphatase
126	Les.4508.1.S1_s_at	Solyc06g074200.2.1	Sex-linked protein 9 (Fragment)
127	LesAffx.36712.1.S1_at	Solyc08g008280.2.1	WRKY transcription factor-30
128	Les.4508.2.S1_s_at	Solyc06g074200.2.1	Sex-linked protein 9 (Fragment)
129	Les.695.1.A1_at		No Hit
130	LesAffx.69957.1.S1_at		No Hit
131	Les.2667.2.S1_at	Solyc06g066370.2.1	WRKY transcription factor 1
132	Les.1314.1.S1_at		No Hit
133	LesAffx.64205.1.S1_at	Solyc04g007450.2.1	Alpha/beta hydrolase fold protein
134	Les.840.1.A1_at		No Hit
135	Les.2915.3.S1_at	Solyc03g117680.2.1	Transcription regulatory protein SNF5
136	Les.788.1.A1_at		No Hit
137	LesAffx.50270.1.S1_at	Solyc02g082900.2.1	Strictosidine synthase family protein
138	LesAffx.46036.1.S1_at	Solyc06g009040.2.1	Glutathione S-transferase
139	LesAffx.8808.2.S1_at	Solyc02g094400.2.1	Glycerophosphodiester phosphodiesterase gde1
140	Les.4047.1.S1_at	Solyc09g091110.2.1	Unknown Protein
141	Les.2316.2.A1_at		No Hit
142	Les.3358.2.S1_at	Solyc10g054440.1.1	Arginine decarboxylase

143	Les.3775.1.S1_at	Solyc05g056070.2.1	Chlorophyll a-b binding protein 6A, chloroplastic
144	Les.3822.1.S1_at		No Hit
145	Les.5157.1.S1_at	Solyc06g074200.2.1	Sex-linked protein 9 (Fragment)
146	Les.2168.1.S1_at	Solyc08g013670.2.1	Photosystem I reaction center subunit
147	Les.2667.3.S1_at		No Hit
148	Les.1409.3.S1_at		No Hit
149	Les.4413.1.A1_at		No Hit
150	Les.4045.1.A1_at		No Hit
151	Les.4493.3.S1_at	Solyc03g079880.2.1	Protease inhibitor/seed storage/lipid transfer protein family protein
152	LesAffx.37707.1.A1_at		No Hit
153	LesAffx.8700.1.S1_at	Solyc03g116550.2.1	AT1G52630-like protein (Fragment)
154	LesAffx.56634.1.S1_at	Solyc04g082960.1.1	TMV response-related protein
155	LesAffx.67630.1.S1_at	Solyc05g013730.2.1	Auxin-independent growth protein (Fragment)
156	Les.113.1.S1_at	Solyc03g116510.1.1	Unknown Protein
157	LesAffx.65210.1.A1_at	Solyc08g075730.2.1	Anamorsin homolog
158	Les.429.1.S1_at	Solyc07g056000.2.1	Xyloglucan endotransglucosylase/hydrolase 7
159	Les.4045.1.S1_at		No Hit
160	LesAffx.3606.1.S1_at	Solyc02g092930.1.1	MYB transcription factor
161	Les.4488.1.S1_at	Solyc09g008670.2.1	Threonine ammonia-lyase biosynthetic
162	Les.3234.1.A1_at	Solyc10g075160.1.1	Ferredoxin I
163	Les.5691.1.S1_at	Solyc07g048120.2.1	Ubiquitin fusion degradation protein 1
164	LesAffx.70768.1.S1_at	Solyc04g007000.1.1	Ethylene-responsive transcription factor 4
165	LesAffx.31773.1.S1_at	Solyc10g007670.2.1	Unknown Protein
166	Les.3866.1.A1_at		No Hit
167	LesAffx.3572.1.S1_at	Solyc01g106690.2.1	Unknown Protein
168	Les.513.1.S1_at	Solyc10g084320.1.1	Subtilisin-like protease
169	Les.3297.1.S1_at	Solyc03g115900.2.1	Chlorophyll a-b binding protein P4, chloroplastic
170	Les.3392.2.S1_at	Solyc08g079170.2.1	Stress-induced protein sti1-like protein
171	LesAffx.50687.1.S1_at	Solyc06g071070.1.1	Short-chain dehydrogenase/reductase family protein
172	Les.4095.1.S1_at	Solyc06g053840.2.1	Auxin responsive protein
173	Les.2620.1.S1_at		No Hit
174	LesAffx.8808.2.A1_at	Solyc02g094400.2.1	Glycerophosphodiester phosphodiesterase gde1
175	LesAffx.10596.1.S1_at	Solyc09g015020.1.1	class I heat shock protein 3
176	LesAffx.44563.1.S1_at	Solyc02g088460.2.1	Chorismate mutase 1
177	Les.1718.1.A1_at		No Hit
178	LesAffx.1.1.S1_at	Solyc01g091170.2.1	Agmatinase
179	Les.2917.1.S1_at		No Hit
180	Les.3063.1.S1_at	Solyc12g099650.1.1	Photosystem II 5 kDa protein, chloroplastic
181	Les.1446.1.A1_at		No Hit
182	Les.84.1.S1_at	Solyc12g010040.1.1	Leucyl aminopeptidase
183	LesAffx.1233.1.S1_at	Solyc06g048820.1.1	Wound-induced protein 1
184	LesAffx.50533.1.S1_at	Solyc02g080070.2.1	RLK, Receptor like protein, putative resistance protein with an antifungal domain"
185	LesAffx.15544.1.S1_at		No Hit
186	Les.2884.1.S1_at	Solyc01g080280.2.1	Glutamine synthetase
187	LesAffx.33190.1.A1_at	Solyc04g005520.2.1	Aspartate/glutamate/uridylate kinase family protein
188	Les.170.1.S1_at	Solyc03g071690.2.1	Non-symbiotic hemoglobin 2
189	LesAffx.70563.1.S1_at	Solyc12g005340.1.1	GRAS family transcription factor
190	Les.3299.2.A1_s_at		No Hit
191	LesAffx.71532.1.S1_at	Solyc10g052880.1.1	LRR receptor-like serine/threonine-protein kinase, RLP"
192	LesAffx.71088.1.S1_at	Solyc02g091720.1.1	Macrophage erythroblast attacher
193	Les.2173.1.A1_at	Solyc09g084490.2.1	Proteinase inhibitor I
194	Les.4042.1.A1_s_at	Solyc09g091110.2.1	Unknown Protein
195	Les.1811.1.S1_at	Solyc11g068460.1.1	Calpain-2 catalytic subunit
196	Les.4008.1.S1_a_at		No Hit
197	LesAffx.34772.1.S1_at	Solyc05g055870.2.1	F-box protein PP2-B1
198	Les.2886.1.A1_at	Solyc02g036370.2.1	MYB transcription factor (Fragment)
199	Les.5641.1.S1_at	Solyc01g099220.2.1	DNA polymerase
200	LesAffx.51979.1.S1_at	Solyc01g111380.2.1	Actin depolymerizing factor 5
201	Les.4046.1.S1_at	Solyc09g091110.2.1	Unknown Protein
202	Les.5843.1.S1_at	Solyc00g257110.2.1	H-ATPase
203	Les.2888.1.S1_at	Solyc04g009030.2.1	Glyceraldehyde-3-phosphate dehydrogenase
204	Les.2861.1.S1_at	Solyc04g009050.2.1	Os01g0498200 protein (Fragment)
205	Les.2328.1.A1_at		No Hit
206	LesAffx.10955.3.S1_at	Solyc06g074530.1.1	Prephenate dehydratase
207	LesAffx.17157.1.S1_at	Solyc08g066860.2.1	Os02g0658033 protein (Fragment)
208	LesAffx.57999.1.S1_at	Solyc01g005430.2.1	UBX domain protein 6
209	LesAffx.56589.2.S1_at	Solyc05g005280.2.1	Poly polymerase catalytic domain containing protein expressed
210	Les.2478.1.S1_a_at		No Hit
211	Les.3869.1.S1_at	Solyc06g082080.2.1	Integrin-linked kinase-associated serine/threonine phosphatase 2C
212	Les.3071.3.S1_at	Solyc07g017610.2.1	Saccharopine dehydrogenase (NAD(+) L-glutamate-forming)
213	Les.1724.1.S1_at	Solyc10g084400.1.1	Glutathione S-transferase
214	Les.3969.1.S1_at	Solyc08g006320.2.1	WRKY transcription factor 3
215	Les.2001.1.S1_at		No Hit
216	Les.2946.2.S1_at	Solyc01g006400.2.1	Cysteine-rich extensin-like protein-4

217	LesAffx.64902.1.S1_at		No Hit
218	LesAffx.48560.1.S1_at	Solyc05g053290.2.1	Protein phosphatase-2C
219	LesAffx.3572.1.A1_at	Solyc01g106690.2.1	Unknown Protein
220	LesAffx.70885.1.S1_at	Solyc03g082840.2.1	Dof zinc finger protein 2
221	LesAffx.9764.1.S1_at	Solyc06g083440.2.1	Cytochrome b5 reductase
222	Les.4044.1.S1_at	Solyc09g091110.2.1	Unknown Protein
223	LesAffx.17345.1.S1_at	Solyc05g053760.2.1	Chaperone protein dnaJ 20
224	LesAffx.37648.1.S1_at	Solyc04g007520.2.1	Phosphatidylserine synthase 2
225	Les.5622.1.S1_at	Solyc03g120090.1.1	Pyridoxal biosynthesis lyase pdxS
226	Les.213.1.S1_at	Solyc06g005470.2.1	Metallothionein-like protein type 2
227	LesAffx.63782.1.S1_at	Solyc02g067920.2.1	Abhydrolase domain-containing protein FAM108B1
228	Les.13.1.S1_at	Solyc04g079730.1.1	cytochrome P450"
229	Les.5126.1.S1_at	Solyc01g107170.2.1	Zinc finger protein
230	Les.4417.1.A1_at	Solyc11g063520.1.1	ORF16-lacZ fusion protein
231	LesAffx.69877.1.S1_at	Solyc01g103020.2.1	Survival of motor neuron-related-splicing factor 30
232	Les.2478.1.S1_at	Solyc03g005770.1.1	Chlorophyll a-b binding protein 3C-like
233	Les.810.1.S1_at	Solyc02g071610.2.1	GDSL esterase/lipase At5g45670
234	Les.4933.1.S1_at	Solyc06g074620.2.1	Os02g0448600 protein (Fragment)
235	LesAffx.64439.1.S1_at	Solyc11g073060.1.1	Zinc finger family protein
236	Les.3308.3.S1_at		No Hit
237	Les.4483.1.S1_at	Solyc04g009440.2.1	NAC domain protein
238	Les.1138.1.A1_at		No Hit
239	LesAffx.8748.1.A1_at	Solyc03g098010.2.1	Acid phosphatase
240	Les.2329.1.A1_at		No Hit
241	Les.4511.1.S1_at		No Hit
242	Les.5055.1.S1_at	Solyc02g071310.2.1	Plasma membrane associated protein
243	LesAffx.69541.1.S1_at	Solyc07g063560.2.1	Cotton fiber expressed protein 1
244	Les.3071.2.S1_at	Solyc07g017610.2.1	Saccharopine dehydrogenase (NAD(+) L-glutamate-forming)
245	LesAffx.62785.1.S1_at	Solyc01g005840.2.1	Ubiquitin-conjugating enzyme E2 E3
246	LesAffx.37213.1.S1_at	Solyc08g076820.2.1	BHLH transcription factor
247	Les.395.1.S1_at		No Hit
248	Les.585.1.A1_at		No Hit
249	LesAffx.51975.2.S1_at	Solyc00g019730.1.1	Cytochrome c oxidase subunit 3
250	LesAffx.3163.2.S1_at	Solyc08g079700.1.1	Zinc finger A20 and AN1 domain-containing stress-associated protein 7
251	Les.5780.1.A1_at	Solyc02g068910.2.1	Peptidase trypsin-like serine and cysteine proteases (Fragment)
252	Les.587.1.S1_at		No Hit
253	LesAffx.26129.1.S1_at	Solyc10g079600.1.1	Response regulator 6
254	Les.2073.2.S1_at	Solyc09g061310.2.1	PPPDE peptidase domain containing 2a
255	LesAffx.8808.1.S1_at	Solyc02g094400.2.1	Glycerophosphodiester phosphodiesterase gde1
256	LesAffx.27925.1.S1_at	Solyc03g019860.2.1	Unknown Protein
257	Les.3818.1.S1_at	Solyc09g089930.1.1	Ethylene responsive transcription factor 1a
258	Les.1888.1.A1_at		No Hit
259	LesAffx.40272.1.S1_at	Solyc01g104800.2.1	Integral membrane protein
260	LesAffx.39538.2.S1_at	Solyc05g056280.2.1	RNA-binding protein Luc7-like 2
261	LesAffx.11839.1.S1_at	Solyc11g056370.1.1	NADH-quinone oxidoreductase subunit
262	LesAffx.68073.1.S1_at	Solyc02g062640.2.1	Pantetheine-phosphate adenylyltransferase
263	LesAffx.56589.1.S1_at	Solyc05g005290.2.1	Poly polymerase catalytic domain containing protein expressed
264	Les.2316.1.S1_at	Solyc03g005450.2.1	Cellulose synthase
265	Les.5164.1.S1_at	Solyc06g063010.2.1	At1g72390-like protein (Fragment)
266	Les.4101.2.S1_a_at	Solyc10g009110.1.1	Ethylene-responsive transcription factor 4
267	LesAffx.9815.1.A1_at	Solyc04g081530.1.1	Chaperone protein dnaJ 11
268	LesAffx.60242.1.S1_at	Solyc10g054320.1.1	Coiled-coil domain-containing protein 90B mitochondrial
269	LesAffx.11231.1.S1_at	Solyc08g067300.1.1	F-box family protein
270	Les.4317.1.S1_at	Solyc06g007180.2.1	Asparagine synthase (Glutamine-hydrolyzing)
271	LesAffx.62593.1.S1_at	Solyc08g082090.1.1	Avr9/Cf-9 rapidly elicited protein 194
272	LesAffx.53941.1.S1_at	Solyc00g217960.1.1	Ring H2 finger protein
273	Les.3006.2.S1_at	Solyc03g053110.2.1	Rhodanese-like family protein-like protein (Fragment)
274	LesAffx.22830.1.S1_at	Solyc02g087210.2.1	Zinc finger AN1 domain-containing stress-associated protein 12
275	Les.3070.2.A1_at		No Hit
276	LesAffx.44496.1.S1_at	Solyc00g171810.2.1	ATP synthase subunit a
277	LesAffx.10613.1.S1_at	Solyc07g005240.2.1	FAD-dependent oxidoreductase family protein
278	Les.4259.1.S1_at	Solyc10g007690.2.1	Chlorophyll a-b binding protein 8, chloroplastic
279	Les.5917.1.S1_at	Solyc07g026650.2.1	1-aminocyclopropane-1-carboxylate oxidase
280	Les.4046.1.A1_at	Solyc09g091110.2.1	Unknown Protein
281	LesAffx.52960.1.S1_at	Solyc07g005520.1.1	Potassium channel tetramerization domain-containing protein
282	Les.3985.1.S1_at		No Hit
283	Les.1650.1.A1_at		No Hit
284	Les.3769.1.S1_at	Solyc08g008100.2.1	1-aminocyclopropane-1-carboxylate synthase
285	Les.424.1.S1_at	Solyc08g076220.2.1	Phosphoribulokinase/uridine kinase
286	LesAffx.1956.1.S1_at	Solyc02g086970.2.1	Aldehyde dehydrogenase 1
287	LesAffx.71454.1.S1_at	Solyc02g082350.2.1	Lrr, resistance protein fragment"
288	LesAffx.71034.1.S1_at	Solyc01g008190.2.1	Os05g0304100 protein (Fragment)
289	Les.3217.1.S1_s_at	Solyc03g034220.2.1	Ribulose bisphosphate carboxylase small chain
290	Les.435.1.S1_at	Solyc05g050130.2.1	Acidic chitinase
291	LesAffx.3163.1.S1_at	Solyc08g079700.1.1	Zinc finger A20 and AN1 domain-containing stress-associated protein 7

292	Les.4041.1.A1_at		No Hit
293	Les.671.1.A1_at		No Hit
294	Les.233.1.S1_at	Solyc07g047850.2.1	Chlorophyll a-b binding protein 4, chloroplastic
298	Les.230.1.S1_at	Solyc09g011670.2.1	Universal stress protein family protein
296	Les.3796.1.S1_at	Solyc08g005900.2.1	Zinc finger and SCAN domain containing 29 (Predicted)
297	LesAffx.62402.1.S1_at	Solyc07g065180.2.1	Decarboxylase family protein
298	Les.4489.1.S1_s_at	Solyc09g097770.2.1	Cell wall protein
299	Les.3157.1.S1_at	Solyc03g097870.2.1	MtN3-like protein
300	LesAffx.59507.1.S1_at	Solyc07g063620.2.1	Uncharacterized secreted protein
301	LesAffx.68186.1.S1_at	Solyc01g086650.2.1	Siroheme synthase
302	Les.3134.1.S1_at	Solyc06g036290.2.1	Heat shock protein 90 (Fragment)
303	Les.3016.1.S1_at	Solyc12g006140.1.1	Chlorophyll a-b binding protein 37, chloroplastic
304	LesAffx.64582.1.S1_at	Solyc02g090980.1.1	Protein serine/threonine kinase
305	LesAffx.71661.1.S1_s_at		No Hit
306	Les.3218.2.A1_at		No Hit
307	Les.770.1.A1_at		No Hit
308	Les.5050.1.S1_at	Solyc01g096230.2.1	ATPase AAA family protein expressed
309	Les.2970.3.A1_at		No Hit
310	Les.3342.1.S1_at	Solyc06g005080.2.1	Vacuolar protein sorting-associated protein 18
311	Les.4725.1.S1_at	Solyc06g008130.2.1	SET domain protein SUVR2
312	Les.4930.1.A1_at	Solyc04g071610.2.1	Water-stress inducible protein 3 (Fragment)
313	Les.4043.1.A1_at	Solyc09g091110.2.1	Unknown Protein
314	LesAffx.66175.1.S1_at	Solyc01g095070.2.1	Heavy metal-associated domain containing protein expressed
315	LesAffx.27729.1.S1_at	Solyc06g065570.2.1	N-acetyltransferase
316	LesAffx.56637.1.S1_at	Solyc06g072330.2.1	Mitochondrial import inner membrane translocase subunit TIM14
317	LesAffx.29104.1.S1_at	Solyc01g112260.2.1	Unknown Protein
318	Les.3515.1.S1_at	Solyc06g083040.2.1	Serine carboxypeptidase 1
319	LesAffx.71623.1.S1_at	Solyc07g063410.2.1	NAC domain protein IPR003441
320	LesAffx.62593.2.S1_at	Solyc08g082090.1.1	Avr9/Cf-9 rapidly elicited protein 194
321	LesAffx.56221.1.S1_at	Solyc10g084370.1.1	MYB transcription factor (Fragment)
322	Les.218.3.S1_at	Solyc02g080210.2.1	Pectinesterase
323	Les.5152.1.S1_at	Solyc11g072630.1.1	Mitogen-activated protein kinase
324	Les.1899.1.A1_at	Solyc03g111860.1.1	Glyoxal oxidase
325	Les.1624.1.A1_at		No Hit
326	LesAffx.57999.1.A1_at	Solyc01g005430.2.1	UBX domain protein 6
327	LesAffx.34084.1.S1_at	Solyc03g116490.1.1	Os05g0176300 protein (Fragment)
328	Les.2917.2.S1_at		No Hit
329	LesAffx.63659.1.S1_at	Solyc04g071030.1.1	U-box domain-containing protein
330	Les.3091.2.S1_at	Solyc01g088370.2.1	Eukaryotic translation initiation factor 3 subunit B
331	Les.1748.1.A1_at		No Hit
332	Les.2003.1.A1_at		No Hit
333	Les.2699.1.S1_at	Solyc04g074100.2.1	Senescence-associated protein
334	Les.4777.1.S1_at	Solyc01g111570.2.1	Receptor-like kinase
335	LesAffx.14059.1.S1_at	Solyc12g005990.1.1	Dual specificity protein phosphatase 4
336	Les.2851.1.A1_at		No Hit
337	LesAffx.66499.2.A1_at	Solyc10g078930.1.1	Activator of heat shock protein ATPase homolog 1
338	LesAffx.45975.1.S1_at	Solyc03g119980.2.1	Lipase-like protein
339	Les.5755.1.S1_at	Solyc06g069570.2.1	SNAP25 homologous protein SNAP33
340	Les.3713.1.S1_at	Solyc04g082840.2.1	Cell division protein kinase 2
341	LesAffx.30683.2.S1_at	Solyc03g115920.2.1	Zinc finger protein-like protein
342	Les.4819.1.S1_at	Solyc06g076020.2.1	heat shock protein
343	Les.248.1.S1_a_at	Solyc01g097270.2.1	Chitinase (Fragment)
344	Les.1434.1.S1_at	Solyc12g009610.1.1	TBC1 domain family member 15
345	LesAffx.35611.1.S1_at	Solyc04g007120.2.1	UV excision repair protein RAD23
346	LesAffx.56.14.S1_at	Solyc01g094170.2.1	Digalactosyldiacylglycerol synthase 2, chloroplastic
347	LesAffx.33349.1.S1_at	Solyc02g077460.1.1	Octanoyltransferase
348	LesAffx.37212.1.S1_at	Solyc03g098020.2.1	Hydrolase alpha/beta fold family protein
349	LesAffx.64861.1.S1_at	Solyc09g098170.2.1	Ras-related protein Rab-25
350	LesAffx.22812.2.S1_at	Solyc03g117860.2.1	IBR finger domain protein
351	LesAffx.10313.1.S1_at	Solyc01g112220.2.1	Serine/threonine protein kinase-like
352	LesAffx.65596.1.S1_at	Solyc07g005890.2.1	ATP-dependent RNA helicase
353	Les.3099.1.S1_at	Solyc06g082940.2.1	Photosystem I reaction center subunit XI
354	LesAffx.62264.2.S1_at	Solyc03g121720.2.1	2-hydroxy-3-oxopropionate reductase
355	LesAffx.54522.1.S1_at	Solyc04g078420.1.1	MYB transcription factor
356	Les.2307.1.A1_at	Solyc02g093680.2.1	Succinate dehydrogenase iron-sulfur protein
357	LesAffx.17017.1.S1_at	Solyc04g007580.1.1	cDNA clone J100026I16 full insert sequence
358	Les.4422.1.A1_at	Solyc01g058410.1.1	Unknown Protein
359	LesAffx.46142.1.S1_at	Solyc07g066340.2.1	UPF0431 protein C1orf66 homolog
360	Les.1910.1.S1_at		No Hit
361	Les.2026.1.S1_at	Solyc10g008400.1.1	RING finger protein 5
362	Les.5920.1.S1_at	Solyc03g044790.2.1	Alpha-hydroxynitrile lyase
363	LesAffx.23563.1.A1_at	Solyc06g076670.2.1	Nuclear SR-like RNA binding protein
364	Les.4137.1.S1_at	Solyc01g100510.2.1	Knotted-like homeobox protein
365	LesAffx.35363.2.A1_at	Solyc12g056410.1.1	F-box protein PP2-B1
366	LesAffx.62402.1.A1_at	Solyc07g065180.2.1	Decarboxylase family protein

367	Les.3799.1.S1_at	Solyc08g060920.2.1	Xenotropic and polytropic retrovirus receptor
368	Les.2620.2.S1_at	Solyc06g084050.2.1	Photosystem II reaction center W protein
369	Les.5914.1.A1_at		No Hit
370	Les.948.1.A1_at		No Hit
371	LesAffx.5975.1.S1_at	Solyc08g068070.2.1	Globin
372	LesAffx.11597.1.S1_at	Solyc02g061950.2.1	C17orf95 protein (Fragment)
373	Les.2672.1.S1_x_at	Solyc06g062540.2.1	Phosphatase
374	Les.3132.1.S1_at	Solyc07g064920.2.1	SWIb domain-containing protein
375	Les.4345.4.A1_x_at		No Hit
376	Les.1409.2.S1_at	Solyc04g081730.2.1	Unknown Protein
377	Les.4895.1.S1_at	Solyc11g006720.1.1	MYB transcription factor
378	LesAffx.12647.1.S1_at	Solyc07g065250.2.1	Serine/threonine protein kinase
379	LesAffx.56634.1.A1_at	Solyc04g082960.1.1	TMV response-related protein
380	Les.4615.1.S1_at	Solyc07g055050.2.1	ATP synthase I-like protein
381	Les.2026.2.A1_at		No Hit
382	LesAffx.58193.1.A1_at	Solyc01g065530.2.1	COBRA-like protein
383	LesAffx.44043.1.A1_at	Solyc11g006500.1.1	Charged multivesicular body protein 5
384	Les.4791.1.S1_at	Solyc06g005710.2.1	cDNA clone 002-143-C11 full insert sequence
385	LesAffx.1574.7.A1_at	Solyc03g115490.1.1	LYR motif-containing protein 4
386	Les.735.1.S1_at		No Hit
387	Les.5167.1.S1_at	Solyc05g052950.2.1	Regulator of chromosome condensation RCC1 domain-containing protein
388	Les.47.1.S1_at	Solyc02g084630.2.1	MADS-box transcription factor
389	LesAffx.71026.1.S1_at	Solyc03g116030.2.1	E3 ubiquitin-protein ligase MARCH3
390	LesAffx.770.1.S1_at	Solyc07g008440.2.1	Purine permease family protein
391	Les.2544.1.A1_at		No Hit
392	Les.5914.1.S1_at		No Hit
393	Les.3523.1.S1_at	Solyc03g116500.2.1	Polygalacturonase
394	Les.2073.1.S1_at	Solyc09g061310.2.1	PPPDE peptidase domain containing 2a
395	Les.1893.1.A1_at		No Hit
396	LesAffx.40158.1.S1_at	Solyc04g071750.2.1	Tubby-like F-box protein 5
397	Les.5886.2.S1_at		No Hit
398	LesAffx.33924.1.S1_at	Solyc03g112440.1.1	Oleosin
399	Les.3608.1.S1_at	Solyc01g006300.2.1	Peroxidase
400	Les.4728.1.S1_at	Solyc12g036220.1.1	Exonuclease DNA polymerase III epsilon subunit family
401	LesAffx.15878.2.A1_at	Solyc01g007020.2.1	U-box domain-containing protein
402	LesAffx.70013.1.S1_at	Solyc03g097200.2.1	Unknown Protein
403	LesAffx.6125.1.S1_at	Solyc09g075670.1.1	Gibberellin receptor GID1L2
404	Les.5916.1.S1_at	Solyc01g100460.2.1	BZIP transcription factor
405	Les.3006.1.A1_at		No Hit
406	Les.5922.1.S1_at	Solyc06g068820.2.1	Mitochondrial import inner membrane translocase subunit TIM16
407	LesAffx.63609.2.S1_at	Solyc11g007590.1.1	OTU domain-containing protein 4
408	Les.1439.2.A1_at		No Hit
409	LesAffx.3797.1.S1_at	Solyc05g005790.2.1	RING finger protein
410	LesAffx.41316.1.S1_at	Solyc03g117850.2.1	Ribulose bisphosphate carboxylase/oxygenase activase
411	LesAffx.61242.1.S1_at	Solyc02g090850.2.1	Splicing factor arginine/serine-rich 16
412	Les.5051.1.S1_at	Solyc05g008220.2.1	Unknown Protein
413	LesAffx.25627.1.S1_at	Solyc05g013170.2.1	Unknown Protein
414	LesAffx.39500.1.S1_at	Solyc03g098590.2.1	RING finger protein 13
415	Les.4345.4.A1_at		No Hit
416	LesAffx.62975.1.S1_at	Solyc03g112340.1.1	Ring H2 finger protein
417	LesAffx.59769.1.S1_at	Solyc01g107980.2.1	U-box domain-containing protein
418	LesAffx.68007.1.S1_at	Solyc04g064510.2.1	Ras-related protein Rab-18
419	LesAffx.730.3.S1_at	Solyc03g083010.2.1	Hydrolase alpha/beta fold family protein
420	LesAffx.69540.1.S1_at	Solyc01g106560.2.1	AT4g33690/T16L1_180 (Fragment)
421	Les.2127.1.A1_at		No Hit
422	Les.2700.1.S1_at		No Hit
423	Les.4083.1.S1_at	Solyc10g006230.2.1	Chlorophyll a-b binding protein 7, chloroplastic
424	Les.3593.1.S1_at		No Hit
425	Les.283.1.S1_at	Solyc01g086960.2.1	Zinc finger A20 and AN1 domain-containing stress-associated protein 6
426	LesAffx.60645.1.S1_at	Solyc01g108620.2.1	Os11g0614900 protein (Fragment)
427	LesAffx.67440.1.S1_at	Solyc02g093530.2.1	Ras-related protein Rab-2-A
428	Les.3343.2.S1_at	Solyc10g080610.1.1	Kelch-like protein 14
429	Les.5044.1.S1_at	Solyc10g081980.1.1	Harpin-induced protein-like (Fragment)
430	Les.4045.1.A1_s_at	Solyc09g091110.2.1	Unknown Protein
431	Les.3314.1.S1_at	Solyc07g056540.2.1	L-lactate dehydrogenase
432	Les.581.2.S1_at		No Hit
433	LesAffx.38388.1.S1_at	Solyc08g081200.2.1	Short-chain dehydrogenase/reductase family protein
434	Les.2377.2.A1_at		No Hit
435	LesAffx.31873.1.S1_at	Solyc10g085740.1.1	GDSL esterase/lipase At5g03820
436	LesAffx.62772.1.S1_at	Solyc11g039950.1.1	Splicing factor 3B subunit 4
437	Les.3357.1.S1_at	Solyc09g066470.2.1	Cobalt import ATP-binding protein CbiO 2
438	Les.3725.1.S1_at	Solyc02g079220.2.1	Solute carrier family 2, facilitated glucose transporter member 8
439	LesAffx.68312.1.S1_at	Solyc07g008050.2.1	RING finger protein
440	LesAffx.3438.1.A1_at	Solyc05g008810.2.1	Lipid phosphate phosphatase 3
441	Les.945.1.A1_at		No Hit

442	LesAffx.29993.1.S1_at	Solyc06g073230.2.1	tRNA-dihydrouridine synthase 2-like protein
443	Les.1603.1.A1_at		No Hit
444	Les.3119.1.A1_at		No Hit
445	Les.5564.1.S1_at	Solyc08g044280.1.1	Unknown Protein
446	Les.3250.3.S1_at		No Hit
447	LesAffx.31716.2.S1_at	Solyc09g010510.2.1	Short-chain dehydrogenase/reductase family protein
448	LesAffx.3299.1.A1_at	Solyc10g006750.2.1	CONSTANS-like zinc finger protein
449	Les.1200.1.A1_at	Solyc12g099410.1.1	Pectinesterase
450	Les.3551.1.S1_at	Solyc01g104740.2.1	Multiprotein bridging factor 1
451	LesAffx.35363.1.S1_at	Solyc12g056410.1.1	F-box protein PP2-B1
452	Les.2377.1.S1_at	Solyc05g025600.1.1	Chloroplast photosystem II subunit X (Fragment)
453	Les.4923.1.S1_at	Solyc10g005080.2.1	Late elongated hypocotyl and circadian clock associated-1-like"
454	Les.943.1.A1_at		No Hit
455	LesAffx.50511.1.S1_at	Solyc06g065740.2.1	Golgi SNAP receptor complex member 1
456	LesAffx.53784.1.S1_at	Solyc07g041830.2.1	MTD1
457	Les.2569.1.S1_at	Solyc05g007770.2.1	NAC domain transcription factor
458	Les.896.2.S1_a_at		No Hit
459	Les.3657.1.S1_at	Solyc10g086410.2.1	Heat shock protein 70-3
460	Les.731.2.A1_at		No Hit
461	LesAffx.57122.1.S1_at	Solyc07g008940.2.1	Os06g0661900 protein (Fragment)
462	LesAffx.5883.1.S1_at		No Hit
463	Les.5420.1.S1_at	Solyc03g079850.2.1	Guanylyl cyclase
464	LesAffx.68459.2.S1_at	Solyc01g010430.2.1	cDNA clone J023121M11 full insert sequence
465	LesAffx.31317.17.A1_at	Solyc01g008370.2.1	26S proteasome regulatory subunit
466	Les.4373.1.S1_at	Solyc01g108250.2.1	Vacuolar import and degradation protein VID27
467	LesAffx.68092.1.S1_at	Solyc06g076350.2.1	Transcription factor (Fragment)
468	Les.4353.1.S1_at	Solyc03g093130.2.1	Xyloglucan endotransglucosylase/hydrolase 9
469	Les.5021.1.S1_at	Solyc09g092430.2.1	Selenium binding protein
470	LesAffx.5017.3.A1_at	Solyc10g080660.1.1	Eukaryotic translation initiation factor 4E
471	Les.5229.1.S1_at	Solyc06g008620.1.1	Protein tolB
472	LesAffx.10807.1.S1_at	Solyc11g066100.1.1	heat shock protein
473	Les.5930.1.S1_at	Solyc12g045030.1.1	Short-chain dehydrogenase/reductase family protein
474	Les.3677.1.S1_at	Solyc05g014280.2.1	Heat shock protein
475	Les.4641.1.S1_at	Solyc01g010050.2.1	S-adenosylmethionine decarboxylase proenzyme
476	Les.1587.1.A1_at		No Hit
477	Les.269.1.S1_at	Solyc03g082420.2.1	Heat shock protein
478	Les.3267.1.S1_at	Solyc08g080570.2.1	UDP-glucose 4-epimerase
479	LesAffx.65213.2.S1_at	Solyc07g062880.2.1	Yippee zinc-binding-like protein
480	Les.4623.1.S1_at	Solyc01g102530.2.1	SNARE associated Golgi protein
481	LesAffx.68802.1.S1_at	Solyc08g028690.2.1	Tasselseed2-like short-chain dehydrogenase/reductase (Fragment)
482	LesAffx.68945.1.S1_at	Solyc01g096740.2.1	Transporter major facilitator family
483	LesAffx.25974.1.A1_at	Solyc06g073450.2.1	Os11g0282300 protein (Fragment)
484	Les.658.1.S1_at		No Hit
485	Les.3297.2.S1_at	Solyc03g115900.2.1	Chlorophyll a-b binding protein P4, chloroplastic
486	Les.3293.3.S1_at	Solyc12g042060.1.1	ATP-dependent clp protease ATP-binding subunit
487	LesAffx.59375.2.A1_at	Solyc10g086420.1.1	SLT1 protein
488	LesAffx.36079.1.S1_at	Solyc06g073230.2.1	tRNA-dihydrouridine synthase 2-like protein
489	LesAffx.35433.1.S1_at	Solyc04g074940.2.1	Bet1-like protein At4g14600
490	LesAffx.37563.1.S1_at	Solyc05g047530.2.1	Cytochrome P450"
491	Les.2457.1.A1_at		No Hit
492	Les.1979.1.A1_at		No Hit
493	Les.3392.3.S1_at	Solyc08g079170.2.1	Stress-induced protein sti1-like protein
494	LesAffx.58193.1.S1_at	Solyc01g065530.2.1	COBRA-like protein
495	Les.5104.1.S1_at	Solyc01g091160.2.1	Agmatinase
496	LesAffx.44043.1.S1_at	Solyc11g006500.1.1	Charged multivesicular body protein 5
497	Les.896.1.S1_at	Solyc12g096520.1.1	Protein phosphatase 2C
498	Les.1258.1.S1_at	Solyc12g006470.1.1	Aminotransferase-like protein
499	LesAffx.21585.1.S1_at		No Hit
500	Les.1699.1.A1_at		No Hit
501	LesAffx.23349.1.S1_at	Solyc07g041720.1.1	Oxalate oxidase-like germin 171
502	LesAffx.64823.1.S1_at	Solyc01g079530.2.1	E3 ubiquitin-protein ligase MARCH6
503	Les.5850.1.S1_at	Solyc12g013710.1.1	Protochlorophyllide reductase
504	Les.5573.1.S1_at	Solyc05g008440.1.1	Unknown Protein
505	LesAffx.35132.1.S1_at	Solyc01g097910.2.1	Rubredoxin family protein
506	Les.3367.1.S1_at	Solyc03g007190.2.1	SPFH domain / Band 7 family protein
507	Les.3781.1.S1_at	Solyc09g091780.2.1	Cyclin dependent kinase inhibitor
508	LesAffx.13805.1.S1_at	Solyc04g081740.2.1	Prostaglandin E synthase 2-like
509	Les.2915.2.S1_at	Solyc03g117680.2.1	Transcription regulatory protein SNF5
510	LesAffx.4763.3.S1_at	Solyc12g087830.1.1	MADS box transcription factor
511	Les.4818.1.S1_s_at	Solyc01g098090.2.1	WD repeat-containing protein
512	Les.2846.1.S1_at		No Hit
513	LesAffx.50112.1.S1_at	Solyc04g071030.1.1	U-box domain-containing protein
514	Les.4575.1.S1_at	Solyc09g074510.2.1	Tubby-like F-box protein 3
515	LesAffx.54246.1.S1_at	Solyc12g008910.1.1	Density-regulated protein
516	LesAffx.12150.1.S1_at	Solyc03g097170.2.1	Cinnamoyl-CoA reductase-like protein

517	LesAffx.65207.2.A1_at	Solyc04g078600.2.1	Genomic DNA chromosome 5 P1 clone MCA23
518	Les.2212.1.A1_at	Solyc01g087990.2.1	MADS-box transcription factor 3
519	Les.5766.1.S1_at	Solyc06g036070.2.1	SCF ubiquitin ligase skp1 component
520	LesAffx.59375.2.S1_at	Solyc10g086420.1.1	SLT1 protein
521	LesAffx.1959.2.S1_at	Solyc03g113620.2.1	MYB transcription factor
522	LesAffx.10016.1.A1_at	Solyc06g069690.2.1	Pseudo response regulator
523	LesAffx.59756.1.S1_at	Solyc07g005640.2.1	26S proteasome non-ATPase regulatory subunit 9
524	Les.2177.1.A1_at		No Hit
525	LesAffx.44296.1.A1_at	Solyc01g079860.2.1	Carbohydrate kinase YjeF related protein
526	LesAffx.69865.1.S1_at	Solyc01g008970.2.1	Heterogeneous nuclear ribonucleoprotein A3-like protein 2
527	LesAffx.51975.1.A1_at		No Hit
528	Les.1121.1.S1_at	Solyc06g084130.2.1	Transmembrane BAX inhibitor motif-containing protein 4
529	LesAffx.63523.1.S1_at	Solyc08g006470.2.1	Zinc finger family protein
530	Les.2878.1.S1_at		No Hit
531	Les.2982.2.S1_at	Solyc09g075090.1.1	Arginine/serine-rich splicing factor
532	LesAffx.8575.1.S1_at	Solyc12g056090.1.1	CHCH domain containing protein
533	LesAffx.65203.1.S1_at	Solyc02g080140.2.1	Cysteine-rich PDZ-binding protein
534	Les.658.2.A1_at		No Hit
535	LesAffx.25416.1.S1_at	Solyc09g082950.1.1	C2 domain-containing protein-like
536	LesAffx.60297.1.S1_at	Solyc01g008810.2.1	Zinc finger family protein
537	LesAffx.51704.1.S1_at	Solyc02g067230.2.1	Dof zinc finger protein
538	Les.1638.2.A1_at		No Hit
539	LesAffx.62742.1.S1_at	Solyc10g085660.1.1	F-box protein SKIP24
540	Les.32.2.S1_a_at	Solyc04g074180.2.1	Cryptochrome 1a"
541	LesAffx.62589.1.S1_at	Solyc06g054580.2.1	Trans-acting transcriptional protein ICP0
542	Les.4258.3.S1_at	Solyc04g054190.2.1	ABC-1 domain protein
543	Les.4912.1.S1_at	Solyc01g098910.2.1	Mitochondrial carrier protein
544	LesAffx.47666.1.S1_at	Solyc10g050060.1.1	Elicitor-responsive protein 3
545	Les.5440.1.S1_at	Solyc05g052190.2.1	Transcription elongation factor A protein 2
546	Les.1529.1.A1_at		No Hit
547	LesAffx.20752.1.S1_at	Solyc10g009200.2.1	Unknown Protein
548	LesAffx.22483.1.S1_at	Solyc03g007310.2.1	Abscisic acid receptor PYL8
549	Les.4867.1.S1_at	Solyc02g080540.1.1	ATP synthase gamma chain
550	LesAffx.59709.1.S1_at	Solyc12g010340.1.1	Pre-mRNA-splicing factor ini1
551	LesAffx.68515.1.S1_at	Solyc12g006060.1.1	Unknown Protein
552	Les.1297.1.S1_at	Solyc07g049180.2.1	Receptor-like protein kinase At5g59670
553	Les.1724.3.A1_at		No Hit
554	LesAffx.64523.1.S1_at	Solyc05g055300.1.1	YIPF5
555	LesAffx.12268.1.S1_at	Solyc01g096610.2.1	UPF0468 protein C16orf80 homolog
556	LesAffx.35363.2.S1_at		No Hit
557	Les.4281.1.A1_at		No Hit
558	LesAffx.43198.1.S1_at	Solyc02g092460.2.1	Speckle-type POZ protein
559	Les.5053.1.S1_at	Solyc06g071670.1.1	Genomic DNA chromosome 5 TAC clone K15C23
560	Les.2475.1.A1_at		No Hit
561	Les.1338.1.A1_at		No Hit
562	LesAffx.8926.1.S1_at	Solyc01g095700.2.1	Abscisic acid receptor PYL8
563	LesAffx.65020.1.S1_at	Solyc08g074420.2.1	Coiled-coil domain-containing protein 130
564	Les.463.1.S1_at	Solyc07g061940.2.1	Acetolactate synthase
565	Les.3578.1.S1_at	Solyc08g062340.2.1	Class II small heat shock protein Le-HSP17.6
566	LesAffx.44565.1.A1_at	Solyc09g075080.2.1	Phytochrome A-associated F-box protein
567	Les.3343.3.S1_at	Solyc10g080610.1.1	Kelch-like protein 14
568	Les.896.2.S1_at		No Hit
569	Les.5927.1.S1_at	Solyc04g005480.1.1	Unknown Protein
570	LesAffx.48382.1.S1_at	Solyc03g026350.2.1	WD-40 repeat family protein
571	Les.4878.1.S1_at	Solyc02g080660.2.1	Glucan endo-1 3-beta-glucosidase 3
572	LesAffx.60693.1.S1_at	Solyc02g065550.2.1	Coiled-coil domain-containing protein 109A
573	LesAffx.65124.1.S1_at	Solyc01g007430.2.1	Cytochrome b6-f complex subunit 5
574	LesAffx.56.14.A1_at	Solyc01g094170.2.1	Digalactosyldiacylglycerol synthase 2, chloroplastic
575	LesAffx.49119.1.S1_at	Solyc02g068360.2.1	Tafazzin
576	LesAffx.3336.1.S1_at	Solyc02g078480.2.1	CBS domain containing protein
577	Les.4008.1.S1_at	Solyc07g052980.2.1	Xyloglucan endotransglucosylase/hydrolase 5
578	LesAffx.63687.1.S1_at	Solyc02g080410.2.1	class I heat shock protein
579	Les.255.2.S1_at	Solyc04g076960.2.1	Sucrose transporter
580	Les.3632.1.S1_at	Solyc03g122340.2.1	Lipoxygenase
581	LesAffx.8710.1.S1_at	Solyc09g072590.2.1	Actin-depolymerizing factor 6
582	Les.2878.2.A1_at		No Hit
583	Les.2380.1.A1_at		No Hit
584	LesAffx.16710.1.S1_at		No Hit
585	LesAffx.37595.1.S1_at	Solyc08g077020.1.1	Unknown Protein
586	Les.5117.1.A1_at	Solyc10g005800.2.1	CWC15 homolog
587	Les.3357.2.A1_at		No Hit
588	Les.1492.2.A1_at		No Hit
589	Les.3108.1.A1_at		No Hit
590	LesAffx.39793.1.S1_at	Solyc11g013080.1.1	Inner membrane ALBINO3-like protein
591	Les.797.1.S1_at	Solyc09g090730.1.1	Ammonium transporter

592	LesAffx.9367.1.S1_at	Solyc01g058720.2.1	NaCl-inducible Ca2+-binding protein
593	Les.4334.3.A1_at	Solyc03g121300.2.1	Myrosinase-binding protein 2
594	Les.5952.1.S1_at	Solyc03g118410.2.1	Acyl carrier protein
595	Les.2979.1.A1_at		No Hit
596	Les.353.1.A1_at		No Hit
597	LesAffx.50750.1.S1_at	Solyc06g065100.2.1	Myb-like transcription factor 1
598	LesAffx.869.2.S1_at	Solyc11g056400.1.1	Ribosomal protein S3 (Fragment)
599	Les.4663.1.S1_at	Solyc03g121910.1.1	Threonine synthase
600	LesAffx.59407.2.S1_at	Solyc09g011360.2.1	Mitochondrial carrier protein expressed
601	LesAffx.55197.1.S1_at	Solyc05g008400.2.1	Repressor of RNA polymerase III transcription MAF1
602	Les.4287.1.S1_at	Solyc03g083730.1.1	Pectinesterase
603	Les.701.1.A1_at		No Hit
604	Les.1409.1.A1_at		No Hit
605	Les.931.1.A1_at		No Hit
606	Les.3267.2.S1_a_at		No Hit
607	LesAffx.65171.1.S1_at	Solyc06g060300.1.1	Unknown Protein
608	Les.581.1.A1_at		No Hit
609	LesAffx.68979.1.S1_at	Solyc06g082470.2.1	Serine/threonine protein kinase
610	LesAffx.17032.1.S1_at	Solyc04g056580.2.1	Homology to unknown gene
611	LesAffx.48314.2.S1_at		No Hit
612	Les.709.1.A1_at		No Hit
613	LesAffx.57700.1.S1_at	Solyc03g117900.2.1	Cytochrome c oxidase assembly protein
614	Les.368.1.S1_at	Solyc01g095580.2.1	GH3 family protein
615	LesAffx.29037.1.S1_at	Solyc06g008690.1.1	Kelch-like protein 14
616	LesAffx.57312.1.S1_at	Solyc01g097190.2.1	Ankyrin repeat protein-like
617	Les.3549.1.S1_at	Solyc02g082760.2.1	Catalase
618	LesAffx.62593.2.A1_at	Solyc08g082090.1.1	Avr9/Cf-9 rapidly elicited protein 194
619	LesAffx.59407.1.S1_at	Solyc09g011360.2.1	Mitochondrial carrier protein expressed
620	Les.3635.1.S1_at	Solyc08g079870.1.1	Subtilisin-like protease
621	Les.2052.1.S1_at	Solyc02g094170.2.1	Zinc finger protein 511
622	Les.415.1.A1_at		No Hit
623	Les.3267.2.S1_at	Solyc08g080570.2.1	UDP-glucose 4-epimerase
624	Les.1889.1.A1_at		No Hit
625	Les.2811.1.S1_at	Solyc03g007520.2.1	Proline-rich cell wall protein-like
626	LesAffx.64104.1.S1_at	Solyc02g070760.2.1	NAD dependent epimerase/dehydratase family protein
627	LesAffx.22898.1.S1_at		No Hit
628	LesAffx.17138.1.S1_at	Solyc02g090870.1.1	Unknown Protein
629	Les.2306.1.A1_at		No Hit
630	Les.218.1.S1_at	Solyc02g080210.2.1	Pectinesterase
631	Les.1159.1.S1_at	Solyc01g087880.2.1	Unknown Protein
632	Les.3752.1.A1_at	Solyc09g082340.2.1	Vicilin-like protein (Fragment)
633	LesAffx.26661.1.S1_at	Solyc08g077630.2.1	ATP binding / serine-threonine kinase
634	Les.1132.1.A1_at	Solyc01g106820.2.1	Peptidase M50 family
635	LesAffx.26661.1.A1_at	Solyc08g077630.2.1	ATP binding / serine-threonine kinase
636	LesAffx.68108.1.S1_at	Solyc06g050700.2.1	Early response to dehydration 15-like protein (Fragment)
637	LesAffx.61269.1.S1_at	Solyc04g082720.2.1	Small heat shock protein
638	Les.4898.1.S1_at	Solyc01g111140.2.1	Protein FAM50 homolog
639	Les.5425.1.A1_at	Solyc10g080070.1.1	GTP-binding family protein
640	LesAffx.70264.1.S1_at	Solyc04g014480.2.1	class I heat shock protein 3
641	Les.1955.1.A1_at		No Hit
642	LesAffx.65616.1.S1_at	Solyc02g088110.2.1	Polypyrimidine tract-binding protein-like
643	LesAffx.71476.1.S1_at	Solyc09g072560.2.1	Legumin 11S-globulin
644	Les.4649.1.S1_s_at		No Hit
645	Les.2931.1.A1_at		No Hit
646	Les.4492.2.S1_at	Solyc01g105050.2.1	Chlorophyll a-b binding protein, chloroplastic
647	LesAffx.25974.1.S1_at	Solyc06g073450.2.1	Os11g0282300 protein (Fragment)
648	Les.2197.1.A1_at	Solyc10g083150.1.1	Retinoblastoma-binding protein 6 (Fragment)
649	Les.3311.3.S1_at	Solyc01g005560.2.1	Isocitrate dehydrogenase
650	LesAffx.15226.1.A1_at	Solyc03g114370.2.1	Atp-dependent RNA helicase
651	LesAffx.5602.1.S1_at	Solyc12g096690.1.1	Cytosolic Fe-S cluster assembly factor nbp35
652	LesAffx.59375.1.A1_at	Solyc10g086420.1.1	SLT1 protein
653	LesAffx.68112.1.S1_at	Solyc01g067070.2.1	Mitochondrial deoxynucleotide carrier
654	LesAffx.70981.2.S1_at	Solyc07g014730.2.1	Phospholipase A2
655	LesAffx.2889.1.A1_at	Solyc09g007760.2.1	Aquaporin 2
656	LesAffx.34223.1.S1_at	Solyc06g071430.2.1	T17H3.1 protein (Fragment)
657	LesAffx.68000.1.S1_at	Solyc01g099860.2.1	Serine/threonine-protein phosphatase
658	Les.2921.1.S1_at	Solyc12g096500.1.1	CONSTANS-like protein
659	Les.2055.1.S1_at	Solyc02g092990.1.1	SCF E3 ubiquitin ligase complex F-box protein grrA
660	Les.5775.1.A1_at	Solyc09g091500.2.1	U6 snRNA-associated Sm-like protein LSm5
661	LesAffx.68828.1.S1_at	Solyc08g007750.2.1	YTH domain containing 1
662	LesAffx.52943.1.S1_at	Solyc06g074130.2.1	Growth inhibition and differentiation-related protein 88
663	LesAffx.67661.1.S1_at	Solyc02g081140.2.1	UBX domain-containing protein
664	LesAffx.66484.1.S1_at	Solyc11g012790.1.1	SEC14-like protein 1
665	LesAffx.49103.1.A1_at	Solyc03g031650.2.1	Autophagy-related protein 8
666	LesAffx.67643.1.S1_at	Solyc04g078460.2.1	N(4)-(Beta-N-acetylglucosaminyl)-L-asparaginase

667	Les.814.1.A1_at	Solyc09g065150.1.1	Unknown Protein
668	Les.2025.1.A1_at		No Hit
668	LesAffx.68556.1.S1_at	Solyc02g083710.2.1	26S proteasome non-ATPase regulatory subunit 4
669	Les.3383.1.S1_at	Solyc03g025720.2.1	Long-chain-fatty-acid--CoA ligase
670	Les.3108.2.S1_at	Solyc11g011140.1.1	50S ribosomal protein L23
671	LesAffx.67661.2.S1_at	Solyc02g081140.2.1	UBX domain-containing protein
672	LesAffx.53035.1.S1_at	Solyc06g068050.2.1	Nucleic acid binding protein
673	LesAffx.63609.1.S1_at	Solyc11g007590.1.1	OTU domain-containing protein 4
674	Les.1517.1.S1_at	Solyc02g082130.1.1	Unknown Protein
675	LesAffx.65922.1.S1_at		No Hit
676	LesAffx.67231.1.S1_at	Solyc05g009910.2.1	Coiled-coil domain-containing protein 94
677	Les.4279.1.A1_at		No Hit
678	LesAffx.70817.1.S1_at	Solyc03g111830.2.1	Protein-L-isoaspartate O-methyltransferase
679	Les.2073.3.A1_at		No Hit
680	Les.4693.1.S1_at	Solyc09g007010.1.1	Pathogenesis related protein PR-1
681	LesAffx.44140.1.S1_at	Solyc12g099390.1.1	Protein DEHYDRATION-INDUCED 19 homolog 4
682	Les.4996.1.S1_at	Solyc08g076960.1.1	Abscisic acid receptor PYR1
683	LesAffx.66436.1.S1_at	Solyc01g005410.2.1	Calcium binding protein Caleosin
684	Les.5683.1.S1_at	Solyc11g006540.1.1	FAD-dependent pyridine nucleotide-disulphide oxidoreductase
685	LesAffx.1091.2.S1_at	Solyc06g071060.1.1	Short-chain dehydrogenase/reductase family protein
686	LesAffx.17420.1.S1_at	Solyc10g005280.1.1	Pentatricopeptide repeat-containing protein

Table 4: List of down-regulated (≥ two-fold) genes in fruits of *AtMYB12*-expressing tomato lines

S.No.	Locus ID	SOLGEN id	Annotation in SOLGEN
1	LesAffx.69645.1.S1_at	Solyc07g052960.1.1	GRAS family transcription factor
2	LesAffx.58502.1.S1_at	Solyc01g108560.2.1	Acetyl esterase
3	Les.3668.1.S1_at	Solyc08g014000.2.1	Lipoxygenase
4	Les.1501.2.S1_at	Solyc02g080510.1.1	Arabidopsis thaliana genomic DNA chromosome 5 P1
5	Les.3980.1.S1_at	Solyc01g006540.2.1	Lipoxygenase
6	LesAffx.24212.1.S1_at	Solyc10g083630.1.1	Repressor of silencing 2b
7	Les.2839.1.S1_at		No Hit
8	Les.3666.1.S1_at	Solyc09g010210.2.1	Endoglucanase 1
9	Les.641.1.S1_at	Solyc08g075870.2.1	Dehydration-responsive family protein-like
10	Les.1501.1.A1_at	Solyc02g080510.1.1	Arabidopsis thaliana genomic DNA chromosome 5
11	Les.4449.1.S1_s_at	Solyc01g008710.2.1	Mannan endo-1 4-beta-mannosidase
12	LesAffx.408.2.S1_at	Solyc10g083630.1.1	Repressor of silencing 2b
13	Les.3630.1.S1_at	Solyc07g064180.2.1	Pectinesterase
14	LesAffx.18025.1.S1_at	Solyc08g006770.2.1	Anthocyanidin synthase (Fragment)
15	Les.5670.1.S1_at	Solyc02g071100.2.1	Purine permease family protein
16	Les.5198.1.S1_at	Solyc02g076680.2.1	Unknown Protein
17	Les.1020.1.A1_a_at		No Hit
18	Les.4463.1.S1_s_at	Solyc10g080210.1.1	Polygalacturonase A
19	LesAffx.6852.1.S1_at	Solyc02g076710.2.1	Cathepsin B-like cysteine proteinase
20	Les.4890.1.S1_at	Solyc03g083090.2.1	Glycogen synthase
21	Les.4880.1.S1_at	Solyc03g122350.2.1	Cytochrome P450"
22	LesAffx.11941.1.S1_at	Solyc02g077040.2.1	Cathepsin B-like cysteine proteinase 5
23	Les.3481.1.S1_a_at	Solyc08g066800.2.1	Phospholipase D
24	Les.68.2.S1_a_at	Solyc08g082250.2.1	Endoglucanase 1
25	Les.3319.3.S1_at	Solyc02g078400.2.1	Allantoinase
26	LesAffx.48114.1.A1_at	Solyc06g061020.2.1	Baculoviral IAP repeat-containing protein 3
27	LesAffx.68047.1.S1_at	Solyc11g065530.1.1	Lipase (Fragment)
28	Les.3232.1.A1_at		No Hit
29	Les.5348.1.S1_at		No Hit
30	Les.3222.2.S1_at	Solyc01g007740.2.1	Peroxiredoxin
31	Les.1108.1.A1_at		No Hit
32	Les.1847.2.S1_at	Solyc06g005970.2.1	Beta-D-glucosidase
33	Les.1847.1.A1_at		No Hit
34	LesAffx.62570.2.S1_at	Solyc01g091690.2.1	Dehydration-responsive family protein
35	Les.2557.1.S1_at	Solyc03g098600.2.1	Oligosaccharyl transferase STT3 subunit
36	Les.191.1.S1_at	Solyc06g051800.2.1	Expansin
37	Les.3500.1.S1_at	Solyc07g056580.2.1	Ethylene receptor
38	Les.18.1.S1_at	Solyc07g062530.2.1	Phosphoenolpyruvate carboxylase 2
39	Les.1455.1.S1_at	Solyc05g009390.2.1	Lipase-like protein
40	Les.2850.1.S1_at	Solyc07g009230.2.1	Unknown Protein
41	LesAffx.24637.1.S1_at	Solyc02g088270.2.1	Genomic DNA chromosome 5 P1 clone MUL8
42	Les.1020.1.A1_at		No Hit
43	Les.2187.1.A1_at		No Hit
44	Les.3122.1.S1_a_at	Solyc07g064170.2.1	Pectinesterase
45	Les.1401.2.S1_at	Solyc01g067660.2.1	Beta-amylase
46	Les.5251.1.S1_at	Solyc02g081970.2.1	Vacuolar sorting receptor 7
47	Les.4697.1.S1_at	Solyc03g121660.2.1	Zinc finger protein
48	Les.3981.1.S1_at	Solyc10g085230.1.1	UDP-glucosyltransferase
49	Les.2341.1.A1_at		No Hit
50	Les.2630.1.S1_at	Solyc05g012260.2.1	Purple acid phosphatase
51	Les.2490.1.S1_at	Solyc11g007020.1.1	Diphosphomevalonate decarboxylase-like protein
52	LesAffx.54523.1.S1_at	Solyc02g087190.1.1	Peroxidase 65
53	Les.2044.1.A1_at		No Hit
54	Les.4447.1.S1_at	Solyc04g049070.2.1	Alpha glucosidase II
55	Les.3527.1.S1_at	Solyc01g099160.2.1	Lipoxygenase
56	LesAffx.46136.1.S1_at	Solyc03g097470.2.1	3-oxoacyl-
57	Les.4445.1.S1_at	Solyc10g047030.1.1	Beta-xylosidase 4
58	Les.5333.1.S1_at	Solyc02g077850.2.1	LRR receptor-like serine/threonine-protein kinase, RLP"
59	Les.15.1.S1_at	Solyc04g078110.1.1	Subtilisin-like protease
60	Les.3319.1.S1_at	Solyc02g078400.2.1	Allantoinase
61	Les.5657.1.S1_at	Solyc04g080080.2.1	Glycosyltransferase family 77 protein
62	Les.1793.1.A1_at		No Hit
63	Les.3465.1.S1_at	Solyc09g089610.2.1	Ethylene receptor
64	Les.3171.4.A1_at	Solyc03g031860.2.1	Phytoene synthase 1"
65	Les.3817.1.S1_at	Solyc07g065980.2.1	Alkaline alpha galactosidase I
66	Les.1401.3.S1_at	Solyc01g067660.2.1	Beta-amylase
67	LesAffx.3570.4.S1_at	Solyc06g064940.2.1	Phosphatidylinositol transfer protein SFH5
68	Les.690.1.A1_at		No Hit
69	Les.5499.1.S1_at	Solyc04g081900.2.1	Nucleoredoxin 1
70	Les.271.1.S1_at	Solyc10g081510.1.1	5-methyltetrahydropteroyltriglutamate--homocysteine methyltransferase

71	Les.251.1.S1_at	Solyc12g044600.2.1	NADP-dependent malic enzyme, chloroplastic
72	Les.5130.1.S1_at	Solyc11g013330.1.1	F2E2.8
73	Les.4312.2.S1_at	Solyc08g074480.1.1	Cortical cell-delineating protein
74	LesAffx.56596.1.S1_at	Solyc12g013820.1.1	Ubiquitin-conjugating enzyme 23
75	Les.601.1.S1_at	Solyc01g009010.2.1	4-diphosphocytidyl-2-C-methyl-D-erythritol kinase
76	Les.4472.1.S1_at	Solyc07g005760.2.1	Hydroxycinnamoyl CoA shikimate/quinate hydroxycinnamoyltransferase
77	Les.1557.1.A1_at		No Hit
78	LesAffx.26531.1.S1_at	Solyc09g013070.2.1	3-oxo-5-alpha-steroid 4-dehydrogenase family protein expressed
79	Les.1401.1.S1_at	Solyc01g067660.2.1	Beta-amylase
80	Les.5092.1.S1_at	Solyc10g038080.1.1	Shikimate dehydrogenase
81	Les.1020.2.A1_at		No Hit
82	Les.278.1.S1_at	Solyc09g010430.1.1	Unknown Protein
83	Les.1179.1.A1_at		No Hit
84	Les.5211.1.S1_at	Solyc03g120720.2.1	Protein disulfide isomerase L-3b
85	Les.5373.1.S1_at		No Hit
86	Les.5250.1.S1_at	Solyc02g093770.2.1	GPI inositol-deacylase
87	Les.5579.1.S1_at		No Hit
88	LesAffx.65152.1.S1_at	Solyc04g015340.2.1	Serine carboxypeptidase K10B2.2
89	LesAffx.69583.1.S1_at	Solyc01g107640.2.1	Tic21
90	Les.5163.1.S1_at	Solyc01g105540.2.1	2-oxoglutarate/malate translocator
91	Les.3654.1.S1_at	Solyc10g080210.1.1	Polygalacturonase A
92	LesAffx.12576.1.S1_at	Solyc12g099800.1.1	Rhodanese-like domain containing protein
93	LesAffx.68271.1.S1_at	Solyc09g066150.1.1	Cytochrome P450"
94	LesAffx.69216.1.S1_at	Solyc01g110450.2.1	NADP dependent sorbitol 6-phosphate dehydrogenase (Fragment)
95	Les.5254.1.S1_a_at		No Hit
96	LesAffx.23309.1.S1_at	Solyc03g116460.2.1	Aspartate racemase
97	Les.33.1.S1_at	Solyc09g010840.1.1	Myb family transcription factor
98	Les.2909.1.S1_at	Solyc07g055060.2.1	Phosphoenolpyruvate carboxylase 1
99	Les.3495.1.S1_at	Solyc06g068090.2.1	Phospholipase D
100	LesAffx.69673.1.S1_at	Solyc01g099040.2.1	GDSL esterase/lipase At5g03980
101	Les.4603.1.S1_at	Solyc12g056540.1.1	Histone H3
102	LesAffx.65348.1.S1_at	Solyc06g006100.2.1	Anthranilate synthase component I family protein expressed
103	LesAffx.35587.1.S1_at	Solyc08g065610.2.1	Vacuolar processing enzyme-1b
104	Les.3625.1.S1_at	Solyc11g011210.1.1	Gibberellin regulated protein
105	Les.259.2.S1_a_at	Solyc03g123760.2.1	Phytoene desaturase"
106	Les.290.1.S1_at	Solyc08g066240.2.1	Decarboxylase family protein
107	Les.2718.3.S1_at	Solyc12g056120.1.1	6-phosphogluconate dehydrogenase decarboxylating
108	Les.1852.1.A1_at		No Hit
109	Les.716.1.S1_at	Solyc08g079070.2.1	Transaldolase
110	LesAffx.49263.1.S1_at	Solyc03g005330.1.1	CBL-interacting protein kinase 20
111	Les.2900.1.S1_at	Solyc02g081160.2.1	Diphosphate-fructose-6-phosphate 1-phosphotransferase
112	Les.4959.1.S1_at	Solyc10g007110.2.1	Tyrosine aminotransferase
113	Les.140.1.S1_at	Solyc08g082820.2.1	Heat shock protein
114	LesAffx.3502.1.S1_at	Solyc01g107800.2.1	Expressed protein (Fragment)
115	Les.61.1.S1_at	Solyc05g007510.2.1	RNA-dependent RNA polymerase
116	Les.5512.1.S1_at	Solyc02g070770.2.1	NAD-dependent epimerase/dehydratase
117	LesAffx.62071.1.S1_at	Solyc05g056130.2.1	Dolichyl-diphosphooligosaccharide--protein glycosyltransferase subunit 1
118	LesAffx.67116.1.S1_at	Solyc10g079200.1.1	Mitochondrial carrier protein
119	LesAffx.43341.1.S1_at	Solyc09g015770.2.1	WRKY transcription factor 6
120	LesAffx.60043.1.A1_at		No Hit
121	Les.2718.1.S1_at	Solyc12g056120.1.1	6-phosphogluconate dehydrogenase decarboxylating
122	Les.3171.3.S1_a_at	Solyc03g031860.2.1	Phytoene synthase 1"
123	LesAffx.69396.1.S1_at	Solyc09g011040.1.1	Aspartic proteinase nepenthesin I
124	Les.4761.1.S1_at	Solyc12g099160.1.1	Serine carboxypeptidase K10B2.2
125	LesAffx.68493.1.S1_at	Solyc08g066700.2.1	Polyribonucleotide nucleotidyltransferase
126	LesAffx.49321.1.S1_at	Solyc03g096640.2.1	Dehydrogenase/reductase SDR family member 4
127	LesAffx.69308.1.S1_at	Solyc02g082670.2.1	WUSCHEL-related homeobox 14
128	Les.409.2.S1_at	Solyc02g093630.2.1	Uncharacterized membrane protein At3g27390
129	Les.3597.1.S1_at	Solyc01g100030.2.1	Deoxyuridine 5&apos-triphosphate nucleotidohydrolase
130	Les.3651.1.S1_at	Solyc09g008280.1.1	S-adenosylmethionine synthase
131	Les.4750.1.S1_at	Solyc12g009410.1.1	Pyruvate dehydrogenase E1 component alpha subunit
132	Les.2541.2.S1_at	Solyc05g054640.2.1	2-oxoglutarate dehydrogenase E1 component
133	LesAffx.8075.1.S1_at	Solyc04g016520.2.1	UDP-N-acetylglucosamine dolichyl phosphate N-acetylglucosamine-1-phosphate transferase
134	Les.4606.1.S1_at	Solyc01g104060.2.1	Aminomethyltransferase
135	Les.1852.2.S1_at	Solyc02g067080.2.1	UDP-D-glucose dehydrogenase
136	LesAffx.46624.1.S1_at	Solyc01g081390.2.1	Glucose 6 phosphate/phosphate translocator-like protein
137	LesAffx.50082.1.A1_at	Solyc06g076750.2.1	Peptide transporter
138	LesAffx.17339.1.S1_at	Solyc04g081210.2.1	Calmodulin binding protein IQ
139	LesAffx.17164.1.A1_at	Solyc01g006450.2.1	Enoyl reductase
140	Les.4099.1.S1_at	Solyc06g053620.2.1	Calcium-dependent protein kinase 2
141	Les.4978.1.S1_at	Solyc01g079500.2.1	DNA replication licensing factor
142	Les.3195.1.S1_at	Solyc05g005020.2.1	R1 protein alpha-glucan water dikinase
143	Les.63.1.S1_at	Solyc11g072310.1.1	Gibberellin 20-oxidase-3
144	LesAffx.61000.1.S1_at	Solyc09g082980.2.1	Plant-specific domain TIGR01615 family protein

145	Les.4426.2.S1_s_at	Solyc06g076140.2.1	Metallothionein-like protein
146	LesAffx.18587.1.S1_at	Solyc02g069800.1.1	CXE carboxylesterase
147	LesAffx.4854.2.S1_at	Solyc12g013690.1.1	Monooxygenase FAD-binding protein
148	Les.2997.1.S1_at	Solyc02g067080.2.1	UDP-D-glucose dehydrogenase
149	Les.270.1.S1_at	Solyc04g074850.2.1	Multidrug resistance protein mdtK
150	LesAffx.11410.1.S1_at	Solyc05g053210.2.1	CBL-interacting protein kinase 1
151	Les.5063.1.S1_at	Solyc03g116110.2.1	Alpha/beta hydrolase fold protein
152	LesAffx.44604.1.S1_at	Solyc09g098040.2.1	Phosphoglucan water dikinase
153	Les.5176.1.S1_at	Solyc06g054020.2.1	Helicase
154	Les.5495.1.S1_at	Solyc09g005850.2.1	Pectate lyase 1
155	Les.5288.1.S1_at	Solyc12g006180.1.1	Heterogeneous nuclear ribonucleoprotein A3
156	Les.3322.2.S1_at	Solyc04g015750.2.1	Magnesium chelatase H subunit
157	Les.3222.1.A1_at		No Hit
158	Les.4029.1.S1_at	Solyc11g069380.1.1	4-hydroxy-3-methylbut-2-en-1-yl diphosphate synthase
159	Les.110.1.S1_at	Solyc11g069270.1.1	Beta-galactosidase
160	LesAffx.56.6.S1_at	Solyc06g053960.2.1	Heat stress transcription factor A3
161	Les.5572.1.S1_at	Solyc11g012930.1.1	Nodulin family protein
162	Les.137.1.S1_at	Solyc11g017240.1.1	Chorismate mutase 2
163	Les.1511.1.A1_at		No Hit
164	LesAffx.12670.2.S1_at	Solyc06g060370.2.1	Organic anion transporter
165	Les.4707.1.S1_at	Solyc03g111690.2.1	Pectate lyase
166	LesAffx.16172.1.S1_at	Solyc07g064270.2.1	Glucose-6-phosphate/phosphate-translocator
167	Les.3279.2.S1_at	Solyc03g120710.2.1	Harpin-induced protein
168	LesAffx.44399.1.S1_at	Solyc02g077700.1.1	Unknown Protein
169	Les.2818.1.S1_at	Solyc08g060940.1.1	Organic anion transporter
170	Les.3627.1.S1_at	Solyc03g095900.2.1	1-aminocyclopropane-1-carboxylate oxidase-like protein
171	Les.2587.1.A1_at		No Hit
172	Les.1511.2.S1_at	Solyc08g077510.2.1	Poly(U)-specific endoribonuclease-A
173	Les.5517.1.S1_at	Solyc05g006030.2.1	Genomic DNA chromosome 5 P1 clone MQB2
174	Les.1725.1.A1_at		No Hit
175	LesAffx.6338.1.S1_at	Solyc09g082210.2.1	Os10g0422600 protein (Fragment)
176	Les.4949.1.S1_at	Solyc04g080730.2.1	Mitogen-activated protein kinase 9
177	Les.4356.2.S1_at	Solyc01g080460.2.1	Pyruvate phosphate dikinase
178	Les.4584.1.A1_s_at	Solyc09g018790.2.1	Gamma hydroxybutyrate dehydrogenase-like protein
179	LesAffx.62189.1.S1_at	Solyc03g031790.1.1	Magnesium transporter protein 1
180	Les.2852.1.S1_at	Solyc08g080640.1.1	Osmotin-like protein (Fragment)
181	LesAffx.30810.1.S1_at	Solyc09g082570.2.1	Neurogenic locus notch protein-like
182	LesAffx.67585.1.S1_at	Solyc01g056720.2.1	Aquaporin SIP12
183	Les.4543.1.S1_at	Solyc03g079920.2.1	WD-repeat protein
184	Les.2582.1.S1_at	Solyc05g054720.2.1	Tic20-like protein
185	LesAffx.65377.1.S1_at	Solyc01g091640.2.1	Dehydration responsive protein
186	Les.5335.1.S1_at	Solyc12g017450.1.1	Cysteine-type endopeptidase/ ubiquitin thiolesterase
187	Les.4664.1.S1_at	Solyc03g112150.1.1	Elongation factor Tu
188	Les.4753.1.S1_at	Solyc02g093080.2.1	1-aminocyclopropane-1-carboxylate oxidase
189	Les.409.1.S1_at	Solyc02g093630.2.1	Uncharacterized membrane protein At3g27390
190	Les.5254.1.S1_at	Solyc05g046330.2.1	Dolichyl-diphosphooligosaccharide--protein glycosyltransferase subunit 2
191	Les.5637.1.S1_at	Solyc07g054450.2.1	Transcription factor (Fragment)
192	LesAffx.60479.1.S1_at	Solyc07g056050.1.1	Transmembrane 9 superfamily protein member 4
193	Les.2585.1.A1_at		No Hit
194	Les.3318.3.S1_at	Solyc05g053810.2.1	Serine hydroxymethyltransferase
195	Les.2973.1.S1_at	Solyc11g011380.1.1	Glutamine synthetase
196	Les.5617.1.S1_at		No Hit
197	Les.5239.1.S1_at	Solyc01g100360.2.1	Dihydrolipoyl dehydrogenase
198	Les.3338.1.S1_a_at		No Hit
199	Les.5740.1.S1_at	Solyc07g018300.2.1	Single-stranded DNA binding protein p30 subunit
200	Les.2512.2.S1_at	Solyc10g083650.1.1	Peroxiredoxin ahpC/TSA family
201	Les.4960.1.S1_at	Solyc05g005490.2.1	Carbonic anhydrase
202	Les.796.1.A1_at		No Hit
203	Les.495.1.S1_at	Solyc04g076790.2.1	Serine hydroxymethyltransferase
204	LesAffx.60939.1.S1_at	Solyc08g023500.2.1	Metal-dependent hydrolase beta-lactamase family
205	Les.357.2.A1_at		No Hit
206	LesAffx.45315.4.S1_at	Solyc11g013810.1.1	Nitrate reductase
207	Les.380.2.A1_at		No Hit
208	Les.2072.1.A1_at		No Hit
209	Les.5214.1.S1_at	Solyc01g067800.2.1	E3 ubiquitin-protein ligase MARCH1
210	Les.4797.1.S1_at	Solyc04g076090.2.1	Glucose-6-phosphate isomerase 2"
211	Les.3194.1.S1_at	Solyc04g053130.2.1	LHC-related protein
212	Les.602.1.A1_at		No Hit
213	LesAffx.56.1.S1_at	Solyc04g072780.2.1	Genomic DNA chromosome 5 TAC clone K14A3
214	LesAffx.55097.1.S1_at	Solyc05g018300.2.1	Protein kinase A-like kinase
215	Les.4678.1.S1_at	Solyc10g086760.1.1	Tubulin beta chain
216	Les.4217.1.S1_at	Solyc07g041900.2.1	Cathepsin L-like cysteine proteinase
217	Les.1298.1.S1_at	Solyc04g076850.2.1	Auxin responsive protein
218	Les.510.3.S1_at	Solyc01g007150.2.1	Coatomer subunit beta-1
219	LesAffx.59668.1.S1_at	Solyc06g061170.2.1	Solute carrier family 25 member 15

220	Les.3649.1.S1_at	Solyc05g053550.2.1	Chalcone synthase
221	Les.1703.1.S1_at	Solyc02g091490.2.1	Fructokinase 3
222	Les.5283.1.S1_at	Solyc07g005020.2.1	DNA replication licensing factor
223	Les.5549.1.S1_at	Solyc08g075810.2.1	Acyl-CoA synthetase/AMP-acid ligase II
224	Les.4080.1.S1_at	Solyc03g113270.2.1	Homeobox-leucine zipper-like protein
225	Les.3661.1.S1_at	Solyc05g050010.2.1	1-aminocyclopropane-1-carboxylate synthase
226	Les.5326.1.S1_at	Solyc01g106780.2.1	Pyruvate kinase
227	Les.2541.1.A1_at		No Hit
228	Les.1256.1.A1_at		No Hit
229	Les.3663.1.S1_at	Solyc02g093580.2.1	Pectate lyase
230	Les.1234.1.A1_at		No Hit
231	Les.1351.1.A1_at	Solyc08g016050.2.1	Dedicator of cytokinesis family protein
232	Les.4443.1.A1_s_at	Solyc02g089160.2.1	Cytochrome P450"
233	Les.2217.1.A1_at		No Hit
234	Les.1662.1.A1_at		No Hit
235	Les.2534.1.S1_at	Solyc09g018790.2.1	Gamma hydroxybutyrate dehydrogenase-like protein
236	Les.167.1.S1_at	Solyc11g066390.1.1	Superoxide dismutase
237	Les.5687.1.S1_at	Solyc09g008920.2.1	Geranylgeranyl pyrophosphate synthase
238	LesAffx.71065.1.S1_at	Solyc04g064870.2.1	Pathogenesis-related protein-like protein
239	Les.4583.1.S1_at	Solyc07g006220.1.1	UDP-D-glucuronate 4-epimerase 1
240	LesAffx.56426.1.S1_at	Solyc07g062220.2.1	Os06g0207500 protein (Fragment)
241	Les.5885.1.A1_at	Solyc04g054910.2.1	Ethylene-responsive transcription factor 13
242	Les.2435.1.A1_at		No Hit
243	LesAffx.53149.1.S1_at	Solyc06g084300.1.1	Unknown Protein
244	LesAffx.30544.1.S1_at	Solyc03g120550.2.1	Peptide transporter 1
245	Les.3416.1.S1_at		No Hit
246	Les.4739.1.S1_at	Solyc05g012070.2.1	Alpha-1 4-glucan-protein synthase
247	Les.4568.1.S1_at	Solyc09g091820.2.1	AT3g24160/MUJ8_16
248	LesAffx.70341.2.S1_at	Solyc04g082670.2.1	Dolichyl-diphosphooligosaccharide--protein glycosyltransferase subunit 1
249	Les.3279.1.A1_at	Solyc03g120710.2.1	Harpin-induced protein
250	LesAffx.10233.1.S1_at	Solyc07g053830.2.1	Mitochondrial ADP/ATP carrier proteins
251	Les.969.1.A1_at		No Hit
252	Les.2718.2.S1_at	Solyc12g056120.1.1	6-phosphogluconate dehydrogenase decarboxylating
253	Les.5631.1.S1_at	Solyc01g111250.2.1	Phosphatidylinositol-specific phospholipase c
254	Les.1939.1.A1_at		No Hit
255	Les.2944.1.A1_at		No Hit
256	LesAffx.48930.1.S1_at	Solyc01g110450.2.1	NADP dependent sorbitol 6-phosphate dehydrogenase (Fragment)
257	LesAffx.59008.1.S1_at	Solyc04g008590.2.1	Pyruvate dehydrogenase E1 component subunit beta
258	LesAffx.51721.1.S1_at	Solyc01g108530.2.1	Acetyl esterase
259	Les.1802.1.A1_at		No Hit
260	Les.3540.1.S1_at	Solyc01g112280.2.1	Succinyl-diaminopimelate desuccinylase
261	Les.3698.1.S1_at	Solyc01g081380.2.1	Nuclear matrix protein 1
262	LesAffx.35173.1.S1_at	Solyc06g076260.2.1	C20orf24 homolog
263	Les.3126.1.S1_at	Solyc01g109880.2.1	BZIP transcription factor
264	Les.4962.1.S1_at	Solyc07g025510.2.1	Senescence-associated protein 5
265	Les.2668.1.S1_at	Solyc01g107390.2.1	Auxin-responsive GH3 product
266	LesAffx.70270.1.S1_at	Solyc11g066820.1.1	Cellulose synthase-like C6 glycosyltransferase family 2
267	LesAffx.56074.1.S1_at	Solyc09g083410.2.1	Amidase hydantoinase/carbamoylase family protein expressed
268	Les.1746.1.A1_at		No Hit
269	LesAffx.52449.2.S1_at	Solyc03g118650.2.1	Aldose 1-epimerase-like protein
270	LesAffx.68367.1.S1_at	Solyc05g051240.1.1	Aspartic proteinase nepenthesin I
271	Les.849.1.A1_at		No Hit
272	Les.3322.3.S1_at	Solyc04g015750.2.1	Magnesium chelatase H subunit
273	Les.275.2.S1_at	Solyc03g111970.2.1	Cytochrome P450"
274	Les.5064.1.S1_at	Solyc01g094790.2.1	Cysteine synthase
275	LesAffx.24096.1.S1_at	Solyc06g007910.2.1	Gibberellin regulated protein
276	LesAffx.42701.1.S1_at	Solyc01g106000.2.1	Isochorismatase hydrolase
277	Les.2756.2.S1_at	Solyc12g096190.1.1	Tryptophan synthase beta chain
278	LesAffx.44584.1.A1_at	Solyc09g007450.2.1	Dolichyldiphosphatase 1
279	Les.3773.1.S1_at	Solyc05g050120.2.1	Malic enzyme
280	Les.4573.1.S1_at	Solyc03g063600.2.1	Guanylate kinase
281	Les.5823.1.S1_at	Solyc06g076970.2.1	Peptidyl-prolyl cis-trans isomerase
282	Les.20.1.S1_at	Solyc01g097810.2.1	Zeta-carotene desaturase"
283	LesAffx.55883.1.S1_at	Solyc08g006720.2.1	Glutathione peroxidase
284	Les.5029.1.S1_at	Solyc10g051200.1.1	Cytochrome c biogenesis protein family
285	Les.3529.1.S1_at	Solyc01g088170.2.1	Aldehyde oxidase
286	LesAffx.52449.1.S1_at	Solyc03g118650.2.1	Aldose 1-epimerase-like protein
287	Les.3962.1.A1_at	Solyc09g066010.2.1	WRKY transcription factor 25
288	Les.5888.1.S1_at	Solyc06g007340.2.1	Gamma-interferon-inducible lysosomal thiol reductase
289	Les.331.1.S1_at	Solyc01g099190.2.1	Lipoxygenase
290	Les.4594.1.S1_at	Solyc06g006080.2.1	Phosphomethylpyrimidine synthase
291	Les.3482.1.S1_at	Solyc11g066440.1.1	Pheophorbide a oxygenase (Fragment)
292	LesAffx.68876.1.S1_at	Solyc11g008870.1.1	Methylenetetrahydrofolate reductase
293	Les.3645.1.S1_at	Solyc02g089160.2.1	Cytochrome P450"
294	Les.1044.1.A1_at		No Hit

298	Les.4296.1.S1_at	Solyc01g110480.2.1	BZIP transcription factor family protein expressed
296	Les.3338.1.S1_at	Solyc10g083970.1.1	S-adenosylmethionine synthase
297	Les.2372.1.A1_at		No Hit
298	Les.4644.1.S1_at	Solyc11g071270.1.1	Class E vacuolar protein-sorting machinery protein HSE1
299	Les.3052.1.S1_at	Solyc06g071960.2.1	Nucleoside diphosphate kinase
300	LesAffx.53061.1.S1_at	Solyc06g050130.2.1	Alpha-galactosidase-like protein
301	LesAffx.37075.2.S1_at	Solyc03g044200.2.1	Alcohol dehydrogenase
302	Les.504.1.A1_at		No Hit
303	LesAffx.71603.1.S1_at		No Hit
304	Les.4242.1.A1_at		No Hit
305	Les.3122.2.A1_a_at	Solyc07g064170.2.1	Pectinesterase
306	Les.4943.1.S1_at	Solyc10g083480.1.1	F-box family protein
307	Les.2044.2.S1_at	Solyc01g104030.2.1	Inward rectifier potassium channel-like protein (Fragment)
308	LesAffx.44598.1.S1_at	Solyc03g044470.2.1	Red chlorophyll catabolite reductase (Fragment)
309	Les.4242.2.S1_at	Solyc04g054980.2.1	Lipoxygenase homology domain-containing protein 1
310	Les.3979.1.S1_at	Solyc12g044880.1.1	Beta-galactosidase
311	Les.514.1.S1_at	Solyc07g042440.2.1	Alkyl hydroperoxide reductase/Thiol specific antioxidant family protein
312	Les.5410.1.S1_at	Solyc01g102350.2.1	Pectinacetylesterase like protein (Fragment)
313	LesAffx.65901.1.S1_at	Solyc08g075750.2.1	ATP-dependent Clp protease proteolytic subunit
314	Les.409.3.S1_at	Solyc02g093630.2.1	Uncharacterized membrane protein At3g27390
315	Les.2668.2.A1_at		No Hit
316	LesAffx.5957.1.S1_at	Solyc05g009370.2.1	50S ribosomal protein L15
317	Les.3319.2.S1_at	Solyc02g078400.2.1	Allantoinase
318	Les.3090.1.S1_at	Solyc11g010230.1.1	Histone H3
319	Les.4979.1.S1_at	Solyc06g068440.2.1	Cinnamoyl-CoA reductase
320	Les.5087.1.S1_at	Solyc01g058730.2.1	Os03g0310500 protein (Fragment)
321	Les.3323.1.S1_at	Solyc02g067180.2.1	Cystathionine gamma synthase
322	Les.2948.1.A1_at		No Hit
323	Les.5264.1.S1_at	Solyc02g092670.1.1	Subtilisin-like protease
324	Les.3933.1.S1_at	Solyc09g092380.2.1	Adenosylhomocysteinase
325	Les.276.1.S1_at	Solyc12g017240.1.1	Xyloglucan endotransglucosylase/hydrolase 7
326	Les.4605.1.S1_at	Solyc06g030470.2.1	Auxin-regulated protein
327	Les.91.1.S1_at	Solyc07g056570.1.1	9-cis-epoxycarotenoid dioxygenase"
328	Les.34.1.S1_at	Solyc01g096190.2.1	Calcium-transporting ATPase
329	Les.3545.1.S1_at	Solyc06g036260.2.1	Beta-carotene hydroxylase 1"
330	LesAffx.69647.2.S1_at	Solyc12g089220.1.1	Wound responsive protein (Fragment)
331	Les.1653.1.A1_at		No Hit
332	Les.5390.1.S1_at	Solyc01g106680.2.1	Unknown Protein
333	Les.1456.1.A1_at		No Hit
334	LesAffx.71261.1.S1_at	Solyc00g009130.2.1	Dehydrogenase/reductase SDR family member 12
335	Les.5885.3.S1_at	Solyc04g054910.2.1	Ethylene-responsive transcription factor 13
336	LesAffx.49296.2.S1_at	Solyc11g012790.1.1	SEC14-like protein 1
337	Les.1389.1.S1_at	Solyc10g075090.1.1	Non-specific lipid-transfer protein
338	Les.845.1.A1_at		No Hit
339	Les.769.1.A1_at		No Hit
340	Les.312.1.A1_at		No Hit
341	Les.3052.3.A1_at		No Hit
342	LesAffx.65505.1.S1_at	Solyc03g118270.1.1	GDP-mannose 4 6-dehydratase
343	Les.109.1.S1_at	Solyc02g084720.2.1	Beta-galactosidase
344	Les.5482.1.S1_at	Solyc06g053300.2.1	Polyadenylate-binding protein 2
345	Les.5282.1.S1_at	Solyc12g095810.1.1	Solute carrier family 15 member 4
346	Les.3340.1.S1_at	Solyc03g113800.2.1	Betaine aldehyde dehydrogenase
347	Les.272.1.A1_at	Solyc05g018700.2.1	Protein disulfide isomerase
348	Les.4630.1.S1_at	Solyc02g070440.2.1	Katanin p60 ATPase-containing subunit A
349	LesAffx.34294.1.S1_at	Solyc07g062180.2.1	Predicted membrane protein
350	LesAffx.45864.1.S1_at	Solyc08g077500.2.1	Vacuolar protein sorting-associated protein
351	Les.3437.1.S1_at	Solyc01g105710.2.1	Peptidyl-prolyl cis-trans isomerase
352	Les.1691.1.S1_at	Solyc06g009400.2.1	Nitrogen regulatory protein P-II
353	LesAffx.4854.1.S1_at	Solyc12g013690.1.1	Monooxygenase FAD-binding protein
354	Les.5221.1.S1_at	Solyc06g053980.2.1	Chlorophyllase 2
355	LesAffx.46272.1.S1_at	Solyc04g056610.2.1	Genomic DNA chromosome 5 P1 clone MDF20
356	Les.1219.1.A1_at		No Hit
357	Les.3388.2.S1_at	Solyc10g083400.1.1	Cytochrome P450"
358	LesAffx.30544.1.A1_at	Solyc03g120550.2.1	Peptide transporter 1
359	LesAffx.69164.1.S1_at	Solyc01g080910.2.1	Unknown Protein
360	Les.5476.1.S1_at	Solyc07g006520.2.1	Preprotein translocase secY subunit
361	Les.3468.1.S1_at	Solyc01g097500.2.1	Serine/threonine kinase
362	LesAffx.31185.1.S1_at	Solyc01g010480.2.1	Potassium voltage-gated channel subfamily H member 8
363	LesAffx.30767.1.S1_at	Solyc08g082870.2.1	Dehydrogenase/reductase SDR family member 13
364	Les.4696.1.S1_at	Solyc01g028830.2.1	ATP binding / serine-threonine kinase
365	Les.1781.1.S1_at	Solyc12g006320.1.1	ATP-dependent RNA helicase
366	LesAffx.3099.1.S1_at	Solyc07g064160.2.1	Thiazole biosynthetic enzyme
367	Les.5701.1.S1_at	Solyc09g061320.2.1	Solute carrier family 35 member F4
368	Les.5780.1.S1_at	Solyc02g068910.2.1	Peptidase trypsin-like serine and cysteine proteases (Fragment)
369	Les.3216.2.S1_at	Solyc03g121270.2.1	IAA-amino acid hydrolase

370	Les.3671.1.S1_at	Solyc03g113400.2.1	H-ATPase
371	Les.3472.1.S1_at	Solyc06g073720.1.1	Ethylene insensitive 3 class transcription factor
372	Les.3776.1.S1_at	Solyc06g071100.2.1	H-ATPase
373	LesAffx.22051.1.S1_at	Solyc04g054480.2.1	C2 domain-containing protein-like
374	LesAffx.58283.3.S1_at	Solyc01g112250.2.1	Calcium-dependent protein kinase 3
375	Les.2756.1.A1_at	Solyc12g096190.1.1	Tryptophan synthase beta chain
376	LesAffx.70148.1.S1_at	Solyc05g018520.2.1	NADH-cytochrome b5 reductase-like protein
377	LesAffx.39916.1.S1_at	Solyc08g081570.2.1	2-C-methyl-D-erythritol 2 4-cyclodiphosphate synthase
378	Les.2564.2.A1_at		No Hit
379	Les.2110.1.A1_at	Solyc11g020670.1.1	TCP family transcription factor
380	Les.19.1.S1_at	Solyc12g014250.1.1	Phosphoenolpyruvate carboxylase 1
381	Les.2909.2.S1_at	Solyc07g055060.2.1	Phosphoenolpyruvate carboxylase 1
382	Les.2627.1.S1_x_at		No Hit
383	Les.3318.1.A1_at		No Hit
384	Les.5089.1.S1_at	Solyc09g016930.1.1	Photosystem II CP43 chlorophyll apoprotein
385	Les.5255.1.S1_at	Solyc11g005080.1.1	Protein tolB
386	LesAffx.44021.1.S1_at	Solyc01g109410.2.1	Dolichyl-diphosphooligosaccharide--protein glycosyltransferase subunit
387	LesAffx.64234.2.S1_at	Solyc10g081440.1.1	NADH cytochrome b5 reductase
388	Les.3641.1.S1_at	Solyc10g076610.1.1	Lysyl-tRNA synthetase
389	LesAffx.9824.1.S1_at	Solyc01g105660.2.1	1-aminocyclopropane-1-carboxylate oxidase
390	Les.4342.1.S1_at	Solyc08g006890.2.1	Tubulin alpha-3 chain
391	Les.51.1.S1_at	Solyc05g007950.2.1	Ribonuclease T2
392	Les.1413.1.A1_at		No Hit
393	LesAffx.19138.1.S1_at	Solyc09g074110.2.1	AGAP009276-PA (Fragment)
394	Les.4666.1.S1_at	Solyc03g025550.2.1	Signal peptide peptidase family protein
395	LesAffx.65474.2.S1_at	Solyc01g107040.2.1	Ribosomal RNA large subunit methyltransferase N
396	Les.5839.1.S1_at	Solyc02g078140.2.1	Protein kinase
397	Les.5802.1.S1_at	Solyc02g082400.2.1	Lysine-specific demethylase 3A
398	Les.4069.1.A1_at		No Hit
399	Les.3883.1.A1_at		No Hit
400	Les.1158.2.A1_at		No Hit
401	Les.1250.2.S1_at	Solyc04g071560.2.1	Thioredoxin y
402	Les.4276.1.S1_at	Solyc11g069430.1.1	Aquaporin 1
403	Les.5371.1.S1_at	Solyc04g007770.2.1	Major latex-like protein
404	LesAffx.70188.1.S1_at	Solyc08g076450.2.1	3-beta hydroxysteroid dehydrogenase/isomerase family protein
405	Les.2627.1.S1_at		No Hit
406	Les.397.1.S1_at	Solyc06g068220.2.1	Hydrolase alpha/beta fold family protein
407	Les.73.1.S1_at	Solyc09g090070.1.1	Inorganic phosphate transporter
408	Les.4921.1.S1_at	Solyc02g076820.2.1	Light-dependent short hypocotyls 1
409	LesAffx.39098.1.S1_at	Solyc10g055740.1.1	Lysine/histidine transporter
410	Les.5124.1.S1_at	Solyc03g120850.2.1	chaperonin
411	LesAffx.69648.2.S1_at	Solyc02g084870.2.1	Mitogen-activated protein kinase 7
412	LesAffx.18872.1.S1_at	Solyc07g005610.2.1	Endonuclease/exonuclease/phosphatase family
413	Les.510.1.A1_at		No Hit
414	Les.2945.1.A1_at		No Hit
415	LesAffx.64583.1.S1_at	Solyc04g007530.2.1	Multidrug resistance protein mdtK
416	LesAffx.21441.2.S1_at	Solyc01g081570.2.1	Carboxyl-terminal peptidase
417	Les.937.1.S1_at		No Hit
418	Les.5090.1.S1_at	Solyc05g050800.2.1	Phosphoglycerate mutase family protein
419	Les.388.1.S1_at	Solyc01g005210.2.1	Alpha alpha-trehalose-phosphate synthase (UDP-forming)
420	Les.2895.1.S1_at	Solyc08g022210.2.1	Methylthioribose-1-phosphate isomerase
421	LesAffx.56104.1.S1_at	Solyc12g006230.1.1	RING-H2 finger protein
422	Les.4740.1.S1_at	Solyc01g008780.2.1	Phospholipase A22
423	Les.2702.1.S1_a_at	Solyc03g083910.2.1	Acid beta-fructofuranosidase
424	LesAffx.40884.1.S1_at	Solyc04g082310.2.1	Seed maturation protein PM23 (Fragment)
425	LesAffx.22051.3.S1_at	Solyc04g054480.2.1	C2 domain-containing protein-like
426	Les.2951.1.S1_at	Solyc11g065990.1.1	Actin
427	Les.5485.1.S1_at	Solyc07g006280.2.1	Senescence-associated protein
428	Les.2820.2.S1_at	Solyc03g065340.2.1	Phosphorylase
429	Les.4439.1.S1_at	Solyc01g079110.2.1	Histone H3
430	Les.1100.1.A1_at		No Hit
431	Les.2938.3.A1_at		No Hit
432	Les.542.1.A1_at		No Hit
433	Les.4986.1.S1_at	Solyc02g085100.2.1	Aldose 1-epimerase family protein
434	LesAffx.22051.5.S1_at		No Hit
435	Les.45.1.S1_at	Solyc08g080650.1.1	Osmotin-like protein (Fragment)
436	LesAffx.61184.1.S1_at	Solyc09g082970.2.1	Pyruvate kinase
437	Les.4005.1.S1_at	Solyc12g055800.1.1	V-type ATP synthase alpha chain
438	Les.5678.1.S1_at	Solyc10g079970.1.1	Transmembrane protein 19
439	Les.1478.1.S1_at	Solyc05g005480.2.1	Oxidoreductase zinc-binding dehydrogenase
440	Les.2929.1.S1_at	Solyc07g017900.2.1	Aldose 1-epimerase family protein
441	Les.5598.1.S1_at	Solyc10g083570.1.1	Fructose-bisphosphate aldolase
442	Les.367.1.A1_at		No Hit
443	Les.1250.3.A1_at		No Hit
444	LesAffx.67281.1.S1_at	Solyc01g111920.2.1	F-box family protein

445	Les.4446.1.S1_at	Solyc06g073730.1.1	Ethylene insensitive 3 class transcription factor
446	Les.5787.1.S1_at	Solyc08g045850.2.1	Pentatricopeptide repeat-containing protein
447	Les.5067.1.S1_at	Solyc03g119530.2.1	LOB domain protein 42
448	Les.4884.1.S1_at	Solyc11g073200.1.1	Legumin 11S-globulin
449	LesAffx.69888.1.S1_at	Solyc01g090900.2.1	Unknown Protein
450	Les.1392.1.A1_at		No Hit
451	LesAffx.64518.1.S1_at	Solyc06g073920.2.1	YABBY-like transcription factor CRABS CLAW-like protein
452	Les.3270.1.S1_at	Solyc08g077240.2.1	Dehydration-responsive family protein
453	Les.407.1.S1_at	Solyc09g005550.2.1	Calcium dependent protein kinase 13
454	LesAffx.2960.1.S1_at	Solyc01g111360.2.1	Peptidyl-prolyl cis-trans isomerase
455	Les.250.1.S1_at	Solyc04g080960.2.1	Cysteine proteinase cathepsin F
456	Les.5866.1.S1_at	Solyc03g063110.2.1	Stearoyl-acyl carrier protein desaturase
457	Les.2373.1.S1_at	Solyc01g088570.2.1	Acyl carrier protein
458	Les.1250.1.S1_at	Solyc04g071560.2.1	Thioredoxin y
459	Les.5060.1.S1_at	Solyc08g081490.2.1	Mitogen-activated protein kinase
460	LesAffx.52932.1.S1_at	Solyc08g076840.2.1	Polyadenylate-binding protein 2
461	Les.5844.1.S1_at	Solyc12g043020.1.1	Dihydroxy-acid dehydratase
462	Les.5699.1.S1_at	Solyc10g055760.1.1	NAC domain protein IPR003441
463	LesAffx.68388.1.S1_at	Solyc04g015340.2.1	Serine carboxypeptidase K10B2.2
464	Les.783.1.S1_at	Solyc10g009210.2.1	Calmodulin-binding protein (Fragment)
465	LesAffx.61379.1.S1_at	Solyc06g072800.2.1	ADP-ribosylation factor
466	Les.4690.1.S1_at	Solyc08g048240.2.1	FolD bifunctional protein
467	LesAffx.55870.1.S1_at	Solyc12g008640.1.1	Gamma-glutamyltransferase-like protein
468	Les.3478.1.S1_at	Solyc07g049690.2.1	Cytochrome P450"
469	LesAffx.51781.1.S1_at	Solyc09g090640.2.1	Unknown Protein
470	Les.2418.1.S1_at		No Hit
471	Les.1977.1.S1_at	Solyc01g091950.2.1	1-acylglycerophosphocholine O-acyltransferase 1
472	Les.2491.2.S1_at	Solyc01g011040.2.1	LRR receptor-like serine/threonine-protein kinase, RLP"
473	Les.4847.1.S1_at	Solyc04g014570.2.1	Transmembrane 9 superfamily protein member 1
474	Les.3643.1.S1_at	Solyc04g081190.2.1	BZIP transcription factor family protein expressed
475	Les.4735.1.S1_at	Solyc08g007790.2.1	Hydroxymethylglutaryl-CoA synthase
476	LesAffx.51348.1.S1_at	Solyc08g074940.2.1	Aspartic proteinase nepenthesin-2
477	Les.2530.1.A1_at		No Hit
478	Les.1212.1.S1_at	Solyc02g030170.2.1	FAD linked oxidase domain protein
479	LesAffx.63813.1.S1_at	Solyc08g075770.2.1	Unknown Protein
480	LesAffx.62832.1.S1_at	Solyc05g007100.2.1	DNA-binding protein p24
481	Les.5808.1.A1_at		No Hit
482	Les.5664.1.S1_at	Solyc03g083560.1.1	Expressed protein (Fragment)
483	Les.1241.1.S1_at	Solyc02g094470.2.1	Mitochondrial phosphate carrier protein
484	Les.4060.1.S1_at	Solyc01g009310.2.1	Sterol reductase
485	Les.5760.1.S1_at	Solyc10g008880.2.1	One zinc finger protein
486	Les.29.1.S1_at	Solyc05g005710.2.1	Spermidine synthase
487	LesAffx.61378.1.S1_at	Solyc06g049070.2.1	Solute carrier family 35 member E3
488	Les.2123.1.A1_at		No Hit
489	Les.3084.1.S1_at	Solyc08g081310.2.1	Os10g0358600 protein (Fragment)
490	Les.3511.1.S1_at	Solyc07g066210.2.1	H(+)/Cl(-) exchange transporter ClcA
491	LesAffx.61848.1.S1_at	Solyc04g079640.2.1	Cytochrome P450"
492	Les.1491.1.S1_at	Solyc08g043180.2.1	Unknown Protein
493	Les.2409.1.S1_at	Solyc05g007490.2.1	Dehydration-responsive family protein
494	Les.2496.2.S1_at	Solyc09g075430.2.1	Ribosomal protein L19
495	Les.2550.1.A1_at		No Hit
496	LesAffx.10650.1.S1_at	Solyc06g072840.2.1	Seed specific protein Bn15D1B
497	LesAffx.67955.1.S1_at	Solyc11g012440.1.1	U-box domain-containing protein 4
498	Les.3813.1.S2_at	Solyc05g010420.1.1	S-adenosylmethionine decarboxylase proenzyme
499	LesAffx.40547.1.S1_at	Solyc03g044720.1.1	Pinoresinol-lariciresinol reductase
500	LesAffx.65474.1.S1_at	Solyc01g107040.2.1	Ribosomal RNA large subunit methyltransferase N
501	Les.3396.1.A1_at		No Hit
502	LesAffx.51189.1.S1_at	Solyc07g062610.2.1	4-methyl-5(B-hydroxyethyl)-thiazole monophosphate biosynthesis enzyme
503	Les.3060.1.S1_at	Solyc02g084440.2.1	Fructose-bisphosphate aldolase
504	Les.1238.1.S1_at	Solyc03g120310.2.1	Glycosyl transferase group 1 family protein
505	Les.3052.2.S1_at	Solyc06g071960.2.1	Nucleoside diphosphate kinase
506	LesAffx.62837.1.S1_at	Solyc02g063490.2.1	Malate dehydrogenase
507	LesAffx.70419.1.S1_at	Solyc09g042750.2.1	Acyl-CoA thioesterase 9
508	Les.1279.1.A1_at		No Hit
509	LesAffx.49065.1.S1_at	Solyc11g071510.1.1	Glycosyl hydrolase (Fragment)
510	Les.2944.3.S1_at	Solyc10g080940.1.1	Tubulin beta chain
511	Les.3308.2.S1_at	Solyc06g034370.1.1	Pectinesterase
512	Les.923.1.S1_at	Solyc07g064610.2.1	Calcium-dependent protein kinase 2
513	Les.2891.1.S1_at		No Hit
514	Les.761.1.A1_at		No Hit
515	Les.2611.2.A1_at		No Hit
516	Les.2868.1.S1_at		No Hit
517	Les.2924.1.S1_at	Solyc10g008740.2.1	Magnesium chelatase ATPase subunit I
518	Les.5369.1.S1_at	Solyc06g054490.2.1	Prolyl 4-hydroxylase alpha subunit-like protein
519	Les.5432.1.S1_at	Solyc09g005590.2.1	SelT-like protein

520	Les.94.1.S1_at	Solyc11g030600.2.1	DNA (Cytosine-5)-methyltransferase
521	Les.4034.1.S1_at		No Hit
522	Les.4987.1.S1_at	Solyc06g075360.2.1	Senescence-associated protein
523	Les.1374.1.A1_at		No Hit
524	LesAffx.4193.1.S1_at	Solyc06g008010.2.1	Mediator of RNA polymerase II transcription subunit 18
525	LesAffx.20507.1.S1_at	Solyc12g005060.1.1	ATP synthase gamma chain
526	Les.4737.1.S1_at	Solyc02g081030.2.1	Uncharacterized membrane protein C2G11.09
527	Les.1307.1.A1_at		No Hit
528	LesAffx.65026.2.S1_at	Solyc10g078400.1.1	Os03g0366700 protein (Fragment)
529	Les.1248.2.S1_a_at	Solyc03g096730.2.1	GDP-D-mannose pyrophosphorylase 1"
530	Les.5213.1.S1_at	Solyc08g014470.2.1	Amidohydrolase family protein
531	Les.2230.1.A1_at		No Hit
532	Les.510.2.S1_at	Solyc01g007150.2.1	Coatomer subunit beta-1
533	Les.3565.1.S1_a_at	Solyc01g059870.2.1	Phytochrome B1"
534	Les.3149.3.S1_at	Solyc10g086010.1.1	60S ribosomal protein L4/L1
535	LesAffx.57287.2.S1_at	Solyc01g099700.2.1	Os07g0673000 protein (Fragment)
536	Les.3263.2.S1_at		No Hit
537	LesAffx.3562.1.S1_at	Solyc08g077350.2.1	HAD-superfamily hydrolase subfamily IA variant 3
538	LesAffx.67937.1.S1_at	Solyc08g067840.2.1	PsbP domain-containing protein 5, chloroplastic
539	Les.1719.1.A1_at		No Hit
540	Les.4756.1.S1_at	Solyc07g063430.2.1	Mpv17 protein
541	LesAffx.62202.2.S1_at	Solyc03g063240.2.1	Pyridoxamine 5&apos-phosphate oxidase-related FMN-binding protein
542	Les.3068.1.S1_at	Solyc06g069170.2.1	Unknown Protein
543	LesAffx.24371.1.S1_at	Solyc03g005020.2.1	Lipase
544	LesAffx.69566.1.S1_at	Solyc01g097800.2.1	Galactose-6-phosphate isomerase subunit lacB
545	Les.3149.1.A1_at		No Hit
546	Les.1925.2.S1_at	Solyc05g054760.2.1	Dehydroascorbate reductase (Fragment)
547	Les.3541.1.S1_at	Solyc09g059970.2.1	Small ubiquitin-related modifier 2
548	LesAffx.26378.1.S1_at	Solyc08g077050.2.1	Ferredoxin family protein
549	LesAffx.37855.1.S1_at	Solyc07g064500.2.1	Purple acid phosphatase
550	Les.1947.1.A1_at	Solyc11g013840.1.1	Exostosin-like glycosyltransferase
551	LesAffx.69151.1.S1_at	Solyc10g081260.1.1	Multidrug resistance protein mdtK
552	Les.2434.1.S1_at	Solyc10g081530.1.1	V-type proton ATPase subunit d 1
553	LesAffx.28567.1.S1_at	Solyc06g073520.2.1	Solute carrier family 35 member F1
554	LesAffx.38907.2.S1_at	Solyc09g015830.1.1	Pto-like, Serine/threonine kinase protein, resistance protein"
555	Les.5682.1.S1_at	Solyc02g078570.2.1	Epoxide hydrolase 3
556	Les.1288.1.S1_at	Solyc01g096660.2.1	Genomic DNA chromosome 5 P1 clone MOJ9
557	LesAffx.63515.1.S1_at	Solyc06g049070.2.1	Solute carrier family 35 member E3
558	Les.4090.1.S1_at	Solyc11g071790.1.1	Unknown Protein
559	Les.2403.1.S1_at	Solyc10g085880.1.1	UDP-glucosyltransferase family 1 protein
560	Les.4315.1.S1_at	Solyc08g081400.2.1	BEL1-like homeodomain protein 2
561	Les.5870.1.S1_at	Solyc09g090190.2.1	Dehydration-responsive protein-like
562	LesAffx.46003.1.S1_at	Solyc12g049370.1.1	Nucleoside diphosphate kinase
563	LesAffx.70446.1.S1_at	Solyc02g086550.2.1	Os06g0661900 protein (Fragment)
564	Les.1291.1.A1_at		No Hit
565	Les.5308.1.S1_at	Solyc12g098990.1.1	Pentatricopeptide repeat-containing protein
566	LesAffx.69647.1.S1_at	Solyc12g089220.1.1	Wound responsive protein (Fragment)
567	Les.5455.1.S1_at	Solyc06g007530.2.1	B3 domain-containing protein Os05g0481400
568	Les.3216.1.S1_at	Solyc03g121270.2.1	IAA-amino acid hydrolase
569	Les.2343.1.A1_at		No Hit
570	Les.5880.1.S1_at	Solyc03g082660.2.1	Major facilitator superfamily domain containing protein 5
571	Les.5551.1.S1_at	Solyc11g020300.1.1	Translocon Tic40
572	Les.5238.1.S1_at	Solyc06g065500.2.1	Pentatricopeptide repeat-containing protein
573	LesAffx.7053.1.A1_at	Solyc09g091240.2.1	Predicted membrane protein (Fragment)
574	Les.1598.1.S1_at	Solyc07g055940.1.1	Homology to unknown gene (Fragment)
575	LesAffx.61402.1.S1_at	Solyc07g021750.1.1	Cytidine deaminase
576	Les.665.1.A1_at		No Hit
577	Les.5235.1.S1_at	Solyc12g056110.1.1	V-type proton ATPase subunit E
578	LesAffx.66165.1.S1_at	Solyc03g098460.2.1	Retinol dehydrogenase 12
579	LesAffx.42008.1.S1_at	Solyc06g073620.2.1	Molybdopterin synthase catalytic subunit
580	LesAffx.27440.1.S1_at	Solyc12g021280.1.1	Protein kinase
581	Les.3456.1.S1_at	Solyc11g007830.1.1	Dual-specificity protein-like phosphatase 3
582	Les.5287.1.S1_at	Solyc02g093150.2.1	AP2-like ethylene-responsive transcription factor At1g16060
583	Les.1330.1.S1_at	Solyc08g062920.2.1	Elongation factor EF-2
584	LesAffx.2303.1.S1_at	Solyc09g082730.2.1	Aldo/keto reductase family protein
585	Les.5358.1.S1_at	Solyc01g111520.2.1	Synaptotagmin
586	LesAffx.64234.1.S1_at	Solyc10g081440.1.1	NADH cytochrome b5 reductase
587	LesAffx.54321.1.S1_at	Solyc07g044830.2.1	Membrane-associated progesterone receptor component 1
588	LesAffx.52290.1.S1_at	Solyc03g111550.2.1	GDSL esterase/lipase At3g48460
589	LesAffx.187.1.S1_at	Solyc12g098710.1.1	15-cis-zeta-carotene isomerase"
590	LesAffx.30182.1.S1_at	Solyc02g082030.2.1	ABC-type transport system-like
591	Les.5515.1.S1_at	Solyc04g050920.2.1	Os02g0504100 protein (Fragment)
592	Les.5605.1.S1_at		No Hit
593	Les.2299.1.A1_at	Solyc03g112010.2.1	Cytochrome P450"
594	Les.5215.1.S1_at	Solyc09g074320.2.1	Serine/threonine-protein phosphatase

595	Les.3692.1.S1_at		No Hit
596	Les.2257.1.A1_at		No Hit
597	Les.2495.1.S1_at	Solyc04g074500.2.1	Serine-threonine protein kinase
598	LesAffx.10095.1.A1_at	Solyc05g009530.2.1	Dihydrolipoyllysine-residue acetyltransferase component of pyruvate dehydrogenase complex
599	LesAffx.71292.1.S1_at	Solyc11g011200.1.1	Post-GPI attachment to proteins factor 3
600	Les.5253.1.S1_at	Solyc06g005890.2.1	Unknown Protein
601	Les.3378.3.S1_at	Solyc09g090140.2.1	Malate dehydrogenase
602	Les.3159.2.S1_at	Solyc08g081320.2.1	AP-1 complex subunit beta-1
603	LesAffx.3110.1.S1_at	Solyc02g070050.2.1	Fatty acid hydroxylase family protein expressed
604	LesAffx.65394.1.S1_at	Solyc07g063070.1.1	DNA-binding WRKY VQ
605	Les.1977.3.S1_at	Solyc01g091950.2.1	1-acylglycerophosphocholine O-acyltransferase 1
606	Les.3544.1.S1_at	Solyc03g007960.2.1	Beta-carotene hydroxylase 2"
607	Les.5711.1.S1_at	Solyc01g110110.2.1	Cysteine proteinase cathepsin F
608	Les.5871.2.S1_at	Solyc02g085350.2.1	Succinate dehydrogenase flavoprotein subunit
609	Les.2373.2.A1_at		No Hit
610	Les.2564.1.S1_at		No Hit
611	Les.2722.1.S1_at	Solyc07g061780.2.1	Ubiquitin carboxyl-terminal hydrolase family protein
612	Les.1563.1.S1_at		No Hit
613	Les.3517.2.S1_a_at	Solyc07g062680.1.1	Transcription factor CYCLOIDEA (Fragment)
614	Les.474.3.S1_at	Solyc08g081250.2.1	Aminopeptidase N
615	LesAffx.65156.1.S1_at	Solyc06g068900.2.1	Nudix hydrolase 4
616	Les.1659.1.A1_at		No Hit
617	Les.1594.1.S1_at	Solyc08g077230.2.1	Two-component response regulator ARR11
618	Les.3813.1.S1_at	Solyc01g010040.2.1	S-adenosylmethionine decarboxylase uORF
619	Les.3723.1.S1_at	Solyc11g072690.1.1	2-dehydro-3-deoxyphosphooctonate aldolase
620	Les.2492.1.S1_at	Solyc07g007510.2.1	Adenylate cyclase
621	Les.3101.1.S1_at	Solyc06g005940.2.1	Protein disulfide isomerase
622	Les.4564.1.S1_at	Solyc07g064970.2.1	Microtubule-associated protein MAP65-1a
623	Les.3483.1.S1_at	Solyc10g081120.1.1	Alpha-L-arabinofuranosidase
624	Les.2491.3.S1_at	Solyc01g011040.2.1	LRR receptor-like serine/threonine-protein kinase, RLP"
625	LesAffx.6688.1.S1_at	Solyc09g092500.1.1	UDP-glucosyltransferase family 1 protein
626	LesAffx.6024.1.S1_at	Solyc01g088660.2.1	Os04g0585900 protein (Fragment)
627	LesAffx.22955.1.S1_at	Solyc10g006010.2.1	Calcium-activated outward-rectifying potassium channel 1
628	Les.1994.3.S1_at	Solyc05g005800.2.1	Threonyl-tRNA synthetase
629	Les.4384.1.S1_at	Solyc09g064370.2.1	Alcohol dehydrogenase
630	Les.4992.1.S1_at	Solyc10g081720.1.1	Fasciclin-like arabinogalactan protein 14
631	Les.5726.1.S1_at	Solyc08g081910.2.1	V-type proton ATPase subunit E
632	Les.5327.1.S1_at	Solyc11g011020.1.1	Receptor like kinase, RLK"
633	LesAffx.68447.1.S1_at	Solyc08g068590.2.1	PAP fibrillin family protein
634	Les.3473.1.S1_at	Solyc03g119080.2.1	Beta-glucosidase
635	LesAffx.66676.1.S1_at	Solyc03g097750.2.1	Translocon-associated protein subunit beta
636	LesAffx.6491.1.S1_at	Solyc10g006050.2.1	Heterogeneous nuclear ribonucleoprotein A3
637	Les.4515.2.S1_at		No Hit
638	Les.4562.1.S1_at	Solyc03g007810.2.1	Pyruvate kinase
639	Les.2144.1.A1_at		No Hit
640	LesAffx.67863.1.S1_at	Solyc11g008590.1.1	UDP-galactose transporter-like protein
641	Les.3350.1.S1_at	Solyc02g088000.2.1	Glycogen synthase
642	Les.1984.1.A1_at		No Hit
643	Les.3419.1.S1_at	Solyc01g111760.2.1	V-type ATP synthase beta chain
644	Les.1083.1.S1_at	Solyc05g053310.2.1	Stress responsive A/B barrel domain family protein
645	Les.5768.1.S1_at	Solyc04g009940.2.1	Heat shock protein binding protein
646	LesAffx.62202.1.S1_at	Solyc03g063240.2.1	Pyridoxamine 5&apos-phosphate oxidase-related FMN-binding protein
647	LesAffx.39665.1.S1_at	Solyc12g056850.1.1	Co-chaperone protein DnaJ
648	LesAffx.63621.1.S1_at	Solyc03g120490.2.1	Genomic DNA chromosome 3 P1 clone MYA6
649	Les.5943.1.S1_at	Solyc12g006970.1.1	Unknown Protein
650	Les.4852.1.S1_at	Solyc08g080680.2.1	Elicitor-responsive protein 3
651	Les.3092.1.S1_at	Solyc01g087260.2.1	Carotenoid cleavage dioxygenase 1B"
652	LesAffx.57437.1.S1_at	Solyc02g065280.2.1	Methyl jasmonate esterase
653	Les.1205.3.S1_at	Solyc10g008680.1.1	Malonyl CoA anthocyanin 3-O-glucoside-6&apos&apos-O-malonyltransferase
654	Les.1781.1.S1_a_at		No Hit
655	Les.3724.1.S1_at	Solyc07g006970.2.1	Solute carrier family 2, facilitated glucose transporter member 5
656	Les.4356.3.S1_at	Solyc01g080460.2.1	Pyruvate phosphate dikinase
657	Les.1829.1.S1_at	Solyc05g008600.2.1	Fructose-bisphosphate aldolase
658	Les.4017.1.S1_at	Solyc07g005550.2.1	Unknown Protein
659	Les.4224.1.A1_at		No Hit
660	Les.5608.1.S1_at	Solyc09g055940.2.1	Bile acid sodium symporter family protein
661	Les.1732.1.S1_at		No Hit
662	Les.3813.1.S1_x_at	Solyc01g010040.2.1	S-adenosylmethionine decarboxylase uORF
663	Les.5329.1.S1_at	Solyc08g081180.2.1	Signal peptide peptidase
664	LesAffx.6163.2.S1_at	Solyc06g036330.1.1	Ornithine cyclodeaminase protein
665	Les.5562.1.S1_at	Solyc03g005230.2.1	Menaquinone biosynthesis methyltransferase ubiE
666	Les.5472.1.S1_at		No Hit
667	LesAffx.65505.2.S1_at	Solyc03g118270.1.1	GDP-mannose 4 6-dehydratase

668	Les.1573.1.A1_at		No Hit
668	Les.2895.2.S1_at	Solyc08g022210.2.1	Methylthioribose-1-phosphate isomerase
669	LesAffx.13365.1.S1_at	Solyc11g010180.1.1	Mitochondrial/chloroplast ribosomal protein L54/L37 (ISS)
670	Les.5545.1.S1_at		No Hit
671	Les.5498.1.S1_at	Solyc06g068740.2.1	Dehydrogenase/reductase SDR family member 7B
672	Les.774.1.A1_at		No Hit
673	Les.471.1.S1_at	Solyc02g068280.2.1	Subtilisin-like serine protease
674	LesAffx.61214.1.S1_at	Solyc03g058920.2.1	Porin/voltage-dependent anion-selective channel protein
675	LesAffx.64600.1.S1_at	Solyc01g105130.2.1	SKIP interacting protein 15
676	LesAffx.44354.1.S1_at	Solyc03g093880.2.1	UPF0406 protein C16orf57 homolog
677	Les.1509.1.S1_at	Solyc09g082970.2.1	Pyruvate kinase
678	Les.1013.1.A1_at		No Hit
679	LesAffx.40726.1.S1_at	Solyc02g063240.2.1	Sterol C-5 desaturase
680	Les.5398.1.S1_at	Solyc04g076430.2.1	Geranylgeranyl transferase type-2 subunit alpha
681	Les.3318.2.S1_at	Solyc05g053810.2.1	Serine hydroxymethyltransferase
682	LesAffx.69782.1.S1_at		No Hit
683	LesAffx.51714.1.S1_at	Solyc05g056490.2.1	3&apos(2&apos) 5&apos-bisphosphate nucleotidase
684	Les.4261.1.S1_at	Solyc05g056290.2.1	Acetyl-CoA carboxylase biotin carboxyl carrier protein
685	Les.275.3.S1_at	Solyc03g111970.2.1	Cytochrome P450"
686	Les.2276.1.S1_at	Solyc08g081920.2.1	Beta-1 3-galactosyltransferase 6
**687**	Les.5350.1.S1_at	Solyc10g008630.2.1	NADH dehydrogenase like protein
688	Les.4330.1.S1_s_at		No Hit
689	Les.2727.1.S1_at	Solyc08g077770.2.1	Calcineurin B-like calcium binding protein
690	Les.2738.1.S1_at	Solyc07g063240.2.1	Genomic DNA chromosome 3 P1 clone MJK13
691	Les.5303.1.S1_at	Solyc07g052830.2.1	Storekeeper protein
692	Les.4540.1.S1_at	Solyc12g009960.1.1	Eukaryotic translation initiation factor 4
693	LesAffx.37075.1.S1_at	Solyc03g044200.2.1	Alcohol dehydrogenase
694	Les.4450.1.S1_at	Solyc05g012020.2.1	MADS-box transcription factor MADS-MC
695	LesAffx.805.1.S1_at	Solyc01g079610.2.1	DNAJ chaperone
696	LesAffx.42553.2.A1_at	Solyc02g089220.2.1	Regulator of chromosome condensation (RCC1)-like protein
697	Les.1925.1.A1_at	Solyc05g054760.2.1	Dehydroascorbate reductase (Fragment)
698	Les.2858.1.S1_at	Solyc06g074120.2.1	BEL1-like homeodomain protein 1
699	LesAffx.28505.1.S1_at	Solyc09g082720.2.1	Aldo/keto reductase family protein
700	LesAffx.3389.1.S1_at	Solyc10g076350.1.1	Macrophage migration inhibitory factor family protein
701	LesAffx.69215.1.S1_at	Solyc11g056680.1.1	LRR receptor-like serine/threonine-protein kinase, RLP"
702	Les.5228.1.A1_at	Solyc10g081470.1.1	Peptidase M50 family protein
703	Les.730.1.S1_at		No Hit
704	Les.4831.1.S1_at	Solyc03g121580.2.1	Histone-binding protein RBBP7
705	Les.1073.1.S1_at	Solyc10g086190.1.1	Adenosine kinase
706	LesAffx.71442.2.S1_at	Solyc08g082170.2.1	Glycoside hydrolase family 28 protein/polygalacturonase family protein
707	Les.5765.1.S1_at	Solyc04g081360.2.1	tRNA (Adenine-N(1)-)-methyltransferase non-catalytic subunit trm6
708	Les.4787.1.S1_at	Solyc11g069360.1.1	Sua5/YciO/YrdC/YwlC family protein
709	Les.4645.1.S1_at	Solyc10g080710.1.1	Asparaginyl-tRNA synthetase 2
710	LesAffx.67648.1.S1_at	Solyc12g088420.1.1	Mpv17 protein
711	Les.3618.1.S1_at	Solyc11g022590.1.1	Kunitz trypsin inhibitor 4 (Fragment)
712	Les.3976.1.S1_at	Solyc06g076360.2.1	Outer envelope membrane protein
713	Les.4843.1.S1_at	Solyc01g079310.2.1	Unknown Protein
714	LesAffx.1345.1.S1_at	Solyc06g068970.2.1	Conserved transmembrane protein
715	LesAffx.22051.2.S1_at	Solyc12g008630.1.1	Mitochondrial processing peptidase alpha subunit
716	Les.5588.1.S1_at	Solyc06g071910.2.1	3-oxoacyl-reductase
717	Les.4461.1.S1_s_at	Solyc06g069430.2.1	MADS box transcription factor
718	Les.2403.2.S1_at	Solyc10g085870.1.1	UDP-glucosyltransferase family 1 protein
719	LesAffx.70810.1.S1_at	Solyc01g102820.2.1	2-C-methyl-D-erythritol 4-phosphate cytidylyltransferase
720	Les.1759.1.A1_at		No Hit
721	Les.1914.1.S1_at	Solyc08g075340.2.1	Glycosyltransferase-like protein
722	Les.1495.1.S1_at	Solyc01g009070.2.1	MYB transcription factor
723	Les.2722.2.S1_at	Solyc07g061780.2.1	Ubiquitin carboxyl-terminal hydrolase family protein
724	Les.1158.1.S1_at		No Hit
725	LesAffx.2072.1.S1_at	Solyc09g009390.2.1	Monodehydroascorbate reductase (NADH)-like protein
726	LesAffx.58351.1.S1_at	Solyc01g100760.2.1	Susceptibility homeodomain transcription factor (Fragment)
727	Les.266.1.S1_a_at		No Hit
728	Les.179.1.S1_at	Solyc01g110440.2.1	Arginine decarboxylase
729	Les.3292.1.A1_at		No Hit
730	Les.1.1.S1_at	Solyc03g121540.2.1	Beta-galactosidase
731	Les.3510.1.S1_at	Solyc01g067890.2.1	1-deoxy-D-xylulose 5-phosphate synthase 1
732	Les.4829.1.S1_at	Solyc12g006380.1.1	1-aminocyclopropane-1-carboxylate oxidase-like protein
733	Les.369.1.S1_at	Solyc02g088100.2.1	Expansin
734	Les.3334.1.S1_at		No Hit
735	Les.2049.1.A1_at		No Hit
736	LesAffx.24384.1.S1_at	Solyc05g012560.1.1	RING zinc finger protein-like
737	Les.4589.1.S1_at	Solyc03g034370.1.1	Unknown Protein
738	Les.5871.1.S1_at	Solyc02g085350.2.1	Succinate dehydrogenase flavoprotein subunit
739	Les.1990.1.S1_at	Solyc04g009710.1.1	Ferredoxin
740	LesAffx.65029.1.S1_at	Solyc03g116740.2.1	Genomic DNA chromosome 3 P1 clone MSJ11
741	LesAffx.58082.1.S1_at	Solyc01g104580.2.1	Harpin-induced protein 1 containing protein expressed

742	Les.184.1.S1_at	Solyc01g101240.2.1	Aspartic proteinase
743	Les.5122.1.S1_at	Solyc08g076410.2.1	Shikimate kinase-like protein
744	LesAffx.62577.1.S1_at	Solyc07g065930.2.1	BTB/POZ domain-containing protein
745	Les.5456.1.S1_at	Solyc08g005070.2.1	ADP-ribosylation factor GTPase-activating protein 3
746	Les.4062.1.S1_at	Solyc01g097570.2.1	Unknown Protein
747	Les.3759.1.S1_at	Solyc01g095150.2.1	Late embryogenesis abundant protein (Fragment)
748	Les.5204.1.S1_at	Solyc01g022740.2.1	Kelch-like protein 21
749	LesAffx.1064.1.S1_at	Solyc11g006550.1.1	Uricase
750	Les.5200.1.S1_at	Solyc08g066570.1.1	Regulator of chromosome condensation RCC1 domain-containing protein
751	Les.4711.1.S1_at	Solyc03g114720.2.1	Transcription factor BIM2
752	LesAffx.64590.1.S1_at	Solyc05g008530.2.1	Sec-independent protein translocase TatC
753	Les.2608.1.S1_at	Solyc11g008900.1.1	Zinc finger CCCH domain-containing protein 66
754	Les.3263.1.A1_a_at		No Hit
755	Les.305.2.S1_at	Solyc06g073540.2.1	Argonaute 4-like protein
756	Les.2868.2.S1_at	Solyc06g051810.2.1	X1 (Fragment)
757	Les.5577.1.S1_at	Solyc11g011910.1.1	Transmembrane 9 superfamily protein member 1
758	Les.5757.1.S1_at	Solyc11g066370.1.1	DNA ligase
759	Les.1852.3.S1_at	Solyc02g088690.2.1	UDP-glucose 6-dehydrogenase
760	Les.3310.3.S1_at	Solyc03g082980.2.1	Nucleic acid binding protein
761	LesAffx.49298.1.S1_at	Solyc09g010970.2.1	Carbonic anhydrase
762	Les.4679.1.S1_at	Solyc01g008340.2.1	50S ribosomal protein L19
763	Les.2118.1.S1_at	Solyc09g065110.2.1	Unknown Protein
764	LesAffx.50639.1.S1_at	Solyc11g007130.1.1	Major facilitator superfamily MFS_1
765	Les.4773.1.S1_at	Solyc04g078750.2.1	Harpin-induced protein
766	Les.4073.1.S1_at	Solyc06g060110.2.1	Amino acid permease
767	Les.4703.1.S1_at	Solyc10g080940.1.1	Tubulin beta chain
768	LesAffx.70949.1.S1_at	Solyc01g006940.2.1	Poly(U)-binding-splicing factor PUF60
769	Les.2085.1.A1_at		No Hit
770	Les.2775.2.S1_at	Solyc09g082650.2.1	Acireductone dioxygenase
771	Les.2480.2.S1_at	Solyc06g054510.2.1	Acyl carrier protein
772	LesAffx.62411.1.S1_at	Solyc01g109930.2.1	Inositol monophosphatase family protein
773	LesAffx.58097.1.S1_at	Solyc07g042590.2.1	ATP binding / serine-threonine kinase
774	Les.1784.1.S1_at	Solyc01g098500.2.1	Glucose transporter 8
775	LesAffx.53474.1.S1_at	Solyc01g107670.2.1	LRR receptor-like serine/threonine-protein kinase, RLP"
776	LesAffx.69888.2.S1_at	Solyc01g090900.2.1	Unknown Protein
777	LesAffx.5043.1.S1_at	Solyc01g006900.2.1	Phosphatidylglycerol/phosphatidylinositol transfer protein
778	Les.2715.1.S1_at	Solyc04g045340.2.1	Phosphoglucomutase
779	Les.487.1.A1_at		No Hit
780	Les.2930.2.S1_at	Solyc01g006430.2.1	Omega-6 fatty acid desaturase
781	LesAffx.22688.1.S1_at	Solyc08g068880.1.1	Os10g0479800 protein (Fragment)
782	Les.4386.1.S1_at		No Hit
783	Les.3180.3.A1_at		No Hit
784	LesAffx.52241.1.A1_at	Solyc06g083110.1.1	Conserved hypothetical membrane protein
785	Les.2896.1.S1_at	Solyc02g014150.2.1	Photosystem II stability/assembly factor Ycf48-like protein
786	Les.178.1.S1_at	Solyc07g065090.1.1	Polygalacturonase inhibitor protein
787	LesAffx.70741.1.S1_at	Solyc06g064570.2.1	SNARE associated Golgi protein
788	Les.3967.1.S1_at	Solyc06g051400.2.1	Omega-3 fatty acid desaturase
789	Les.1898.2.A1_at		No Hit
790	Les.5659.1.S1_at	Solyc09g031940.1.1	Chloroplast lumen common protein family-like protein
791	Les.5360.1.S1_at	Solyc06g083890.2.1	Pre-mRNA-splicing factor SLU7-A
792	Les.474.1.S1_at	Solyc08g081250.2.1	Aminopeptidase N
793	Les.5382.1.S1_at	Solyc03g121440.2.1	Receptor-like protein kinase At3g21340
794	Les.5357.1.S1_at	Solyc03g025220.2.1	Multidrug resistance protein mdtK
795	Les.569.1.A1_at		No Hit
796	Les.2948.2.S1_at	Solyc01g111240.2.1	Translocase of chloroplast 90, chloroplastic
797	Les.3195.2.S1_at	Solyc05g005020.2.1	R1 protein alpha-glucan water dikinase
798	Les.2583.1.S1_at	Solyc12g056790.1.1	NAC domain protein IPR003441
799	Les.4675.1.S1_at	Solyc12g056530.1.1	Fructose-1 6-bisphosphatase class 1
800	Les.2722.3.A1_at		No Hit
801	Les.165.2.S1_at	Solyc09g082780.2.1	Asparagine synthetase B
802	Les.5818.1.S1_at	Solyc07g017240.1.1	Unknown Protein
803	Les.2944.1.A1_s_at		No Hit
804	Les.1431.1.A1_at		No Hit
805	LesAffx.32840.1.S1_at	Solyc06g084570.2.1	Methyltransferase
806	Les.4277.1.A1_at	Solyc01g111830.2.1	24-sterol C-methyltransferase
807	Les.81.1.S1_at	Solyc02g086870.2.1	Farnesyltransferase beta subunit
808	Les.4521.1.S1_at	Solyc08g005770.2.1	Alcohol acetyltransferase
809	Les.5299.1.S1_at	Solyc02g037510.2.1	Amino acid transporter
810	Les.5022.1.S1_at	Solyc08g065260.2.1	Ycf36 protein
811	LesAffx.11856.1.S1_at	Solyc01g091580.2.1	Signal recognition particle-docking protein FtsY
812	Les.2324.1.S1_at		No Hit
813	Les.380.1.S1_at	Solyc02g021640.2.1	Phototropic-responsive NPH3 family protein
814	Les.4405.1.A1_at		No Hit
815	LesAffx.67147.1.S1_at	Solyc05g055760.2.1	Isopentenyl-diphosphate delta-isomerase
816	LesAffx.68296.1.S1_at	Solyc09g009500.2.1	Hydrolase alpha/beta fold family protein

817	Les.1181.1.A1_at		No Hit
818	Les.2334.1.A1_at		No Hit
819	Les.4811.1.S1_at	Solyc07g049730.2.1	TO62-3 (Fragment)
820	LesAffx.71304.1.S1_at	Solyc01g111150.2.1	Translocon-associated protein alpha subunit
821	Les.5666.1.S1_at	Solyc01g101090.2.1	TBC1 domain family member CG11727
822	Les.2821.2.S1_at	Solyc02g077100.2.1	Lipase-like protein
823	LesAffx.68547.1.S1_at	Solyc06g008760.1.1	Glutaredoxin
824	Les.4828.1.S1_at	Solyc01g104880.2.1	Phosphatidylserine decarboxylase proenzyme 2
825	Les.2421.1.A1_at		No Hit
826	Les.3264.3.S1_at	Solyc04g079270.2.1	Sorting and assembly machinery component 50 homolog
827	LesAffx.16474.1.S1_at	Solyc03g026010.1.1	Transmembrane protein 161B
828	Les.1898.1.S1_at	Solyc04g080810.2.1	Ubiquitin-conjugating enzyme E2 W
829	Les.2745.1.S1_at	Solyc04g016360.2.1	S-formylglutathione hydrolase
830	LesAffx.5781.1.S1_at	Solyc06g082980.2.1	3-beta-hydroxysteroid-Delta8 Delta7-isomerase
831	Les.452.1.S1_at	Solyc03g114950.2.1	Lipid a export ATP-binding/permease protein msba
832	Les.5734.1.S1_at	Solyc12g098440.1.1	RPM1 interacting protein 4 transcript 2
833	Les.3378.1.S1_at	Solyc09g090140.2.1	Malate dehydrogenase
834	Les.1484.1.A1_at		No Hit
835	Les.2032.1.A1_at		No Hit
836	LesAffx.70335.1.S1_at	Solyc06g060690.2.1	Receptor-like protein kinase At5g59670
837	LesAffx.66137.1.S1_at	Solyc02g085080.2.1	Transmembrane protein 53
838	Les.3263.3.S1_at	Solyc01g009990.2.1	Peptidyl-prolyl cis-trans isomerase
839	Les.4285.1.S1_s_at	Solyc10g080710.1.1	Asparaginyl-tRNA synthetase 2
840	Les.2855.1.S1_at	Solyc12g009020.1.1	Protein kinase
841	Les.305.3.S1_at	Solyc06g073540.2.1	Argonaute 4-like protein
842	LesAffx.164.1.A1_at	Solyc11g068550.1.1	Os12g0119800 protein (Fragment)
843	LesAffx.68199.1.S1_at	Solyc06g069440.2.1	Zinc finger CCCH domain-containing protein 55
844	LesAffx.69182.2.S1_at	Solyc07g049200.2.1	Unknown Protein
845	LesAffx.70371.1.S1_at	Solyc04g007500.1.1	RING finger protein
846	Les.2559.1.A1_at		No Hit
847	Les.4590.1.S1_at		No Hit
848	Les.2491.1.A1_at		No Hit
849	Les.2820.1.S1_at	Solyc03g065340.2.1	Phosphorylase
850	LesAffx.69677.1.S1_at	Solyc10g011870.2.1	Reticulon family protein
851	Les.3266.2.S1_at	Solyc08g076970.2.1	Acetylornithine deacetylase or succinyl-diaminopimelate desuccinylase

Supplementary Table 5: List of the primers used in the present study

Oligo name	Sequence (5' to 3')	Purpose
AtMYB12	ATGATATGATACGACGTGGAATAG	For *AtMYB12* cDNA isolation
AtMYB12	GTCAGTGGAATAAACACATATAAC	For *AtMYB12* cDNA isolation
AtMYBXba1	TATAACCGCTCTAGAAAATGGGAAGAG*	For cloning in plant expression vector
AtMYBSac1	CGGATCAGAGCTCAATATCATCATCATGAC*	For cloning in plant expression vector
M13F	TGTAAAACGACGGCCAG	Confirmation of inserts and sequencing
M13R	CAGGAAACAGCTATGACC	Confirmation of inserts and sequencing
SlPAL For	AACCTATCTCGTGGCTCTTT	For Real time PCR (Tomato)
SlPAL Rev	TCTTTTTCGCTGAATCTTGC	For Real time PCR (Tomato)
Sl 4CL For	ACACACAAAGGCTTAGTCACGA	For Real time PCR (Tomato)
Sl4CL Rev	AACAGAGGCAACACACACATCA	For Real time PCR (Tomato)
SlCHS Far	TGGTCACCGTGGAGGAGTATC	For Real time PCR (Tomato)
SlCHS Rev	GATCGTAGCTGGACCCTCTGC	For Real time PCR (Tomato)
SlCHI Far	GTTTTTCACAAACCAACAGTTCTGAT	For Real time PCR (Tomato)
SlCHI Rev	GAAGCAGTGCTCGATTCCATAAT	For Real time PCR (Tomato)
SlF3H Far	CACACCGATCCAGGAACCAT	For Real time PCR (Tomato)
SlF3H Rev	GCCCACCAACTTGGTCTTGTA	For Real time PCR (Tomato)
SlF3'H Far	GCACCACGAATGCACTTGC	For Real time PCR (Tomato)
SlF3'H Rev	CGTTAGTACCGTCGGCGAAT	For Real time PCR (Tomato)
SlFLS Far	GAGCATGAAGTTGGGCCAAT	For Real time PCR (Tomato)
SlFLS Rev	TGGTGGGTTGGCCTCATTAA	For Real time PCR (Tomato)
SlANS Far	GAACTAGCACTTGGCGTCGAA	For Real time PCR (Tomato)
SlANS Rev	TTGCAAGCCAGGCACCATA	For Real time PCR (Tomato)
SlGT Far	CGAACGACGAAACACTGTTGA	For Real time PCR (Tomato)
SlGT Rev	TGCAGCATAGATGGCATTGG	For Real time PCR (Tomato)
SlHCT Far	AGGTGAAAAACTCAACGATGGT	For Real time PCR (Tomato)
SlHCT Rev	ACACTAGGCGTGTGGAAATTAG	For Real time PCR (Tomato)
SlASR1 Far	CCTGTTCCACCACAAGGACAA	For Real time PCR (Tomato)
SlASR1 Rev	GTGCCAAGTTTACCGATTTGC	For Real time PCR (Tomato)
SlAQ Far	GCTGCTAATATCTCCGG	For Real time PCR (Tomato)
SlAQ Rev	GCACCCAAAAGTTGAGC	For Real time PCR (Tomato)
SlACP Far	CAAGGACACACTCCGTGACA	For Real time PCR (Tomato)
SlACP Rev	GGCTGATCAAACACCGTAGC	For Real time PCR (Tomato)
SlGST Far	TGGCAAGCCCATTTGTGAGT	For Real time PCR (Tomato)
SlGST Rev	AACGCGCTAAAGCTCGATCA	For Real time PCR (Tomato)
SlERF1A Far	TGTGGGAGCAGTACCGTGGA	For Real time PCR (Tomato)
SlERF1A Rev	CGCGTGAGATCCAGCTCCAG	For Real time PCR (Tomato)

SlACP Far	CAAGGACACACTCCGTGACA	For Real time PCR (Tomato)
SlACP Rev	GGCTGATCAAACACCGTAGC	For Real time PCR (Tomato)
SlAux Far	TTCCTGCTGATGCCCGCAAA	For Real time PCR (Tomato)
SlAux Rev	CGCAAACATCAAAGCCGGTGT	For Real time PCR (Tomato)
SlCyt450 Far	TTCGCGGATCGACCAATCGT	For Real time PCR (Tomato)
SlCyt450 Rev	TGCCTACAGCCAGAGACCCT	For Real time PCR (Tomato)
SlPDH Far	GATCAGCCGGTTCGTCCCAG	For Real time PCR (Tomato)
SlPDH Rev	TGCCAGTCAGCTCTTGACGC	For Real time PCR (Tomato)
SlCrot Far	CTCAATGGGCGGAGGTGCAT	For Real time PCR (Tomato)
SlCrot Rev	AACCGCAGTCCGTGTGGAAG	For Real time PCR (Tomato)

*Underlined sequences represent recognition sites for the enzymes, used for cloning in plant expression vector
